# Low incubation temperature during early development negatively affects survival and related innate immune processes in zebrafish larvae exposed to lipopolysaccharide

**DOI:** 10.1038/s41598-018-22288-8

**Published:** 2018-03-07

**Authors:** Qirui Zhang, Martina Kopp, Igor Babiak, Jorge M. O. Fernandes

**Affiliations:** Faculty of Biosciences and Aquaculture, Nord University, 8049 Bodø, Norway

## Abstract

In many fish species, the immune system is significantly constrained by water temperature. In spite of its critical importance in protecting the host against pathogens, little is known about the influence of embryonic incubation temperature on the innate immunity of fish larvae. Zebrafish (*Danio rerio*) embryos were incubated at 24, 28 or 32 °C until first feeding. Larvae originating from each of these three temperature regimes were further distributed into three challenge temperatures and exposed to lipopolysaccharide (LPS) in a full factorial design (3 incubation × 3 challenge temperatures). At 24 h post LPS challenge, mortality of larvae incubated at 24 °C was 1.2 to 2.6-fold higher than those kept at 28 or 32 °C, regardless of the challenge temperature. LPS challenge at 24 °C stimulated similar immune-related processes but at different levels in larvae incubated at 24 or 32 °C, concomitantly with the down-regulation of some *chemokine* and *lysozyme* transcripts in the former group. Larvae incubated at 24 °C and LPS-challenged at 32 °C exhibited a limited immune response with up-regulation of hypoxia and oxidative stress processes. *Annexin A2a*, *S100 calcium binding protein A10b* and *lymphocyte antigen-6*, *epidermis* were identified as promising candidates for LPS recognition and signal transduction.

## Introduction

In teleosts, the innate immune system is extremely important for host defence. The integumental physical barrier, which consists of skin, gill, gut and associated mucus are effective in preventing pathogens from adhering to the surface of fish^1,2^. Moreover, the mucus contains various antimicrobial substances, such as mucins, lysozymes, proteases, apolipoproteins, natural antibodies, and matrix metallopeptidase. Also, a variety of antimicrobial peptides are present, including cathelicidins, piscidins, defensins, hepcidins, and pardaxins, which not only function in pathogen cell lysis but also have roles in phagocytic chemotaxis, mast cell degranulation, and phagocytosis^3^. In most cases, the above surface barriers and associated factors are sufficient to defend the host against pathogens. If this first line of defence is breached, pathogens will encounter additional humoral immune mediators. Some mucosal antimicrobial components, such as lysozymes, proteases, complement components, also have functions in fish blood. For instance, lectins not only opsonise pathogens and prevent them from adhering to mucosal surfaces, but also activate the complement system in blood^4^. The complement system is crucial in targeting and lysing pathogens. For instance, complement component 3b (C3b) opsonises pathogens and presents them to phagocytic leukocytes, while C5b, C6, C7, C8, and C9 are able to form the membrane attack complex and lyse pathogens^5^. In addition to humoral regulators, cellular components also have key roles in the host defence. Once triggered by pathogens, leukocytes proliferate rapidly within a short time and are led by chemoattractants to infected sites. Cytokines, including interleukins (ILs), tumour necrosis factors (TNFs), chemokines, and interferons (IFNs), are also released by phagocytes; these are essential for regulation of pro- and anti-inflammatory responses^6^.

Pathogen-associated molecular patterns (PAMPs) are recognized by pattern recognition receptors (PRRs) on the surface of leukocytes and other cells involved in the innate immune response. Toll-like receptors (TLRs) are one of the most important PRR families conserved among vertebrates. In vertebrates, they are phylogenetically grouped into six major families and each TLR family recognizes distinct PAMP ligand types^7^. To date, 17 TLRs have been identified in teleosts, and some paralogues have been subjected to extensive duplications^8^. Following signal transduction, the nuclear factor-κB (NF-κB) is activated and released from its inhibitor protein IκB through phosphorylation and then transferred into the nucleus where it binds to DNA to activate the transcription of pro-inflammatory cytokine genes, such as *tnfα*, *il1β*, *il6*, and *ifnγ*^9^.

Teleosts lack some immune organs that are important in mammalian host defence. For instance, the bone marrow, which is the main immune organ of mammals for production of haematopoietic stem cells is absent in teleosts; its function is replaced by the pronephros and thymus^10^. Also, teleosts lack lymph nodes and germinal centres, which results in poor antibody affinity maturation and a limited immunoglobulin (Ig) repertoire^10^. Compared to the innate immune system, the adaptive immune system in fish takes longer to become fully functional^10,11^. For instance, in zebrafish (*Danio rerio*) the thymus is morphologically mature at 3 weeks post-fertilization (wpf), T cells are detectable at 4–6 wpf, and secreted Igs are measurable at 4 wpf^12^. The adaptive immune system also requires additional time to respond. For example, in Japanese eel (*Anguilla japonica*) at least two weeks are needed for the antibody production^13^. Therefore, teleosts rely heavily on the innate immune system, particularly during their early ontogeny.

As most fish are ectotherms, their body temperature changes following ambient thermal fluctuations. This is extremely challenging for the innate immune system during early stages of ontogeny in spite of their high developmental plasticity^14^. It has been demonstrated that the early thermal environment has a profound influence on various phenotypes in adults, such as muscle growth^15^, swimming performance^16^, reproduction^17^, thermal tolerance^18^, and sex determination^19^. However, little is known about the influence of environmental temperature on the developmental plasticity of the innate immune system. A few studies have focused on how temperature affects the post-larval stages of fish, but have not examined embryogenesis^20,21^. The only exception is a recent study in sea bream (*Sparus aurata*, L.) reporting the persistent thermal effect of embryonic development on the plasticity of the hypothalamus-pituitary-interrenal axis and immune function in adult fish^22^.

In this study, we investigated the thermal plasticity of innate immunity in zebrafish during early development. Lipopolysaccharide (LPS) is an endotoxin from Gram-negative bacteria with well characterized immunostimulatory and inflammatory properties in fish^23^. We used LPS to mimic a bacterial challenge and the mRNA transcriptome was analysed to evaluate the global innate immune response at early larval stages.

## Results

### Lipopolysaccharide challenge

**Figure 1:**
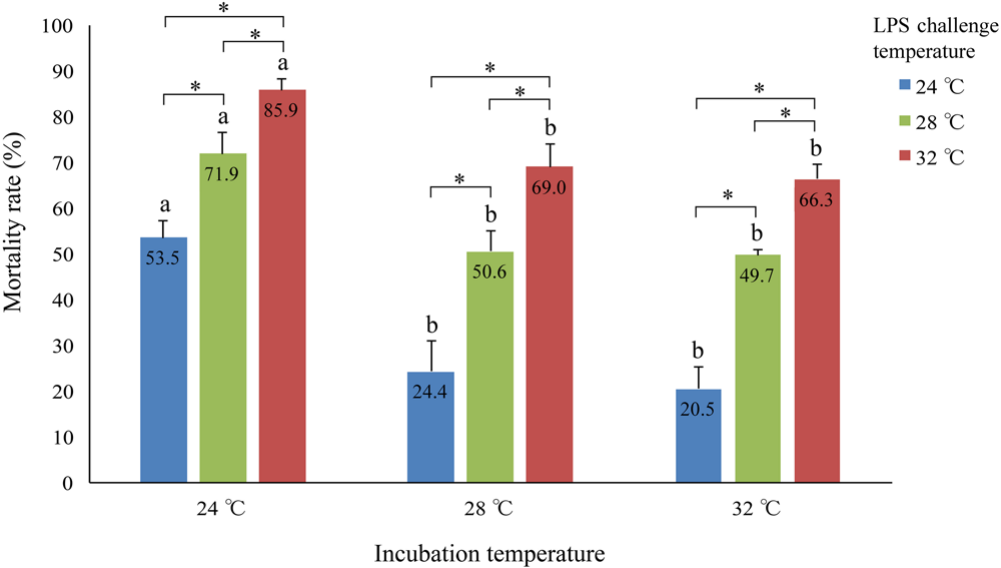
Mortality of larvae after LPS challenge. Mortality rates are represented as mean ± s.d. of triplicates. Significance was analysed using two-way ANOVA. Asterisks indicate the significant (*p*-value < 0.05) difference within the same incubation temperature group, while letters (“**a**”) and (“**b**”) indicate significant (*p*-value < 0.05) differences within the same LPS challenge temperature.

Mortality rates in control groups ranged from 0 to 3% (Supplementary Table S1). In LPS treatment groups, mortality rates of larvae originating from the 24 °C incubation temperature were significantly higher than those from 28 °C and 32 °C incubation temperature groups, regardless of the subsequent challenge temperatures applied (Fig. 1). For instance, at the challenge temperature of 24 °C, the mortality rate of larvae from the incubation temperature of 24 °C was 53.5%, compared to 24.4% and 20.5% in larvae from the incubation temperatures of 28 °C and 32 °C, respectively. No significant difference in mortality was observed between 32 °C and 28 °C incubation groups regardless of subsequent challenge temperatures. For larvae originating from the same incubation temperature, challenge temperatures of 24 °C and 32 °C resulted in the lowest and highest mortality rates, respectively (Fig. 1). In particular, after incubation at 24 °C, the mortality rates of larvae were 53.5% and 85.9% at the challenge temperature of 24 °C and 32 °C, respectively.

### RNA sequencing and mapping

Over 376 million raw reads were obtained by RNA-seq, of which 84.1% had a quality score Q ≥ 30 (Table 1). After adapter and quality trimming, 361,234,698 clean reads were retained. Finally, 267,108,269 reads were successfully mapped to zebrafish transcriptome and genome, and 248,189,243 (92.9%) of them were uniquely mapped, including 122,554,742 read pairs (Supplementary Table S2).

**Table 1:** Summary of library read statistics. In total, 18 libraries, including three LPS treatment replicates and three control replicates from each of three temperature groups (Incubation 24 °C × Challenge 24 °C, Incubation 24 °C × Challenge 32 °C, Incubation 32 °C × Challenge 24 °C), were paired-end sequenced on a NextSeq 500 (Illumina).

	Minimum	Maximum	Total
Raw reads	11,339,354	48,009,280	376,254,382
Trimmed reads	10,897,668	46,119,584	361,234,698
≥Q30 reads	9,344,199	39,781,550	311,105,664
Mapped reads	8,183,262	34,199,926	267,108,269

**Table 2:** Representative genes between LPS-treated and control groups. List of selected DEGs with a fold change greater than 1.5, determined by DESeq2 (adjusted *p*-value < 0.05, Benjamin-Hochberg method).

Gene Name	Description	Fold Change	Padj
Incubation 24 °C × Challenge 24 °C			
*mmp13a*	matrix metallopeptidase 13a	8.5	<0.001
*il1β*	interleukin 1, beta	5.3	<0.001
*fosl1a*	FOS-like antigen 1a	3.6	<0.001
*lye*	lymphocyte antigen-6, epidermis	3.4	<0.001
*anxa2a*	annexin A2a	2.9	<0.001
*cxcl8a*	chemokine (C-X-C motif) ligand 8a	2.2	<0.001
*irf6*	interferon regulatory factor 6	2.0	<0.001
*cxcl8b*.*1*	chemokine (C-X-C motif) ligand 8b, duplicate 1	2.0	<0.001
*tnfrsf11b*	tumor necrosis factor receptor superfamily, member 11b	1.8	0.004
*il6st*	interleukin 6 signal transducer	1.6	0.002
*muc5*.*2*	mucin 5.2	−5.5	<0.001
*muc5*.*1*	mucin 5.1	−3.6	<0.001
*lyz*	lysozyme	−2.3	<0.001
*ccl20a*.*3*	chemokine (C-C motif) ligand 20a, duplicate 3	−2.1	<0.001
*ccl20b*	chemokine (C-C motif) ligand 20b	−1.9	0.002
*caspbl*	caspase b, like	−1.8	0.007
*ctss2*.*2*	cathepsin S, ortholog 2, tandem duplicate 2	−1.7	0.008
*mpeg1*.*2*	macrophage expressed 1, tandem duplicate 2	−1.7	0.034
*apoa4b*.*1*	apolipoprotein A-IV b, tandem duplicate 1	−1.7	0.020
*lta4h*	leukotriene A4 hydrolase	−1.5	0.030
Incubation 32 °C × Challenge 24 °C			
*mmp9*	matrix metallopeptidase 9	5.8	<0.001
*mmp13a*	matrix metallopeptidase 13a	4.5	<0.001
*ptgs2b*	prostaglandin-endoperoxide synthase 2b	3.0	<0.001
*fosl1a*	FOS-like antigen 1a	2.5	<0.001
*lye*	lymphocyte antigen-6, epidermis	2.4	<0.001
*cxcl8b*.*1*	chemokine (C-X-C motif) ligand 8b, duplicate 1	2.3	<0.001
*anxa2a*	annexin A2a	2.2	<0.001
*il1β*	interleukin 1, beta	2.1	0.002
*s100a10b*	S100 calcium binding protein A10b	1.9	0.002
*cebpβ*	CCAAT/enhancer binding protein (C/EBP), beta	1.8	0.050
*muc5*.*2*	mucin 5.2	−3.7	<0.001
*muc5*.*1*	mucin 5.1,oligomeric mucus/gel-forming	−2.3	<0.001
Incubation 24 °C × Challenge 32 °C			
*irg1l*	immunoresponsive gene 1, like	6.3	<0.001
*mmp9*	matrix metallopeptidase 9	4.8	<0.001
*timp2b*	TIMP metallopeptidase inhibitor 2b	4.4	<0.001
*igfbp1a*	insulin-like growth factor binding protein 1a	3.2	<0.001
*lect2l*	leukocyte cell-derived chemotaxin 2 like	2.7	0.001
*lye*	lymphocyte antigen-6, epidermis	2.6	<0.001
*tnfrsf11b*	tumor necrosis factor receptor superfamily, member 11b	2.5	0.006
*mgst3b*	microsomal glutathione S-transferase 3b	2.5	<0.001
*gsto2*	glutathione S-transferase omega 2	2.3	<0.001
*prg4b*	proteoglycan 4b	2.2	0.015
*irf6*	interferon regulatory factor 6	2.1	0.003
*gpx1b*	glutathione peroxidase	2.1	0.029
*mb*	myoglobin	2.1	0.009
*junba*	jun B proto-oncogene a	2.1	0.003
*itgav*	integrin, alpha V	1.9	0.010
*hspd1*	heat shock 60 protein 1	1.7	0.006
*hsp90b1*	heat shock protein 90, beta (grp94), member 1	1.6	0.020
*muc5*.*2*	mucin 5.2	−3.5	<0.001
*mpeg1*.*2*	macrophage expressed 1, tandem duplicate 2	−2.0	0.008
*hmgb1b*	high mobility group box 1b	−1.5	0.030

**Table 3:** Representative Gene Ontology processes regulated by LPS exposure. GO processes were enriched from DEGs by the clusterProfiler package (adjusted *p*-value < 0.05, Benjamin-Hochberg method). Enrichment values are defined as the ratio between GeneRatio and BgRatio. GeneRatio is the ratio of the number of genes that are annotated to a particular biological process over the size of the list of genes of interest. BgRatio is the ratio of the number of genes annotated to the biological term in the background distribution over the total number of genes in the background distribution.

Process	Gene-Ratio	BgRatio	Enrich-ment	Padj	Genes
Incubation 24 °C × Challenge 24 °C Up-regulated					
response to bacterium	7/84	119/14763	10.3	<0.001	*tnfrsf1α*/*cebpβ*/*mmp9*/*junba*/ *cxcl18b*/*il1β*/*junbb*
response to external biotic stimulus	8/84	186/14763	7.6	<0.001	*nfkbiαa*/*tnfrsf1a*/*cebpβ*/*mmp9*/*junba*/*cxcl18b*/*il1β*/*junbb*
positive regulation of response to external stimulus	3/84	21/14763	25.1	<0.001	*mmp9*/*cxcl18b*/*il6st*
myeloid leukocyte activation	3/84	24/14763	22.0	<0.001	*cxcl18b*/*il1β*/*cxcl8b*.*1*
leukocyte chemotaxis	5/84	49/14763	16.9	<0.001	*mmp13a*/*cxcl18b*/*cxcl8b*.*1*/ *il1β*/*cxcl8a*
defense response	7/84	281/14763	4.4	0.001	*ptgs2b*/*tnfrsf1α*/*mmp9*/*il1β*/ *cxcl18b*/*cxcl8b*.*1*/*cxcl8a*
response to wounding	9/84	200/14763	7.9	<0.001	*f3b*/*mmp9*/*sdc4*/*hbegfa*/*il6st*/ *f2rl1*.*2*/*cxcl8b*.*1*/*junbb*/*cxcl8a*
Incubation 24 °C × Challenge 24 °C Down-regulated					
response to xenobiotic stimulus	4/91	49/14763	13.2	<0.001	*foxq1a*/*si:ch211-117m20*.*5*/ *im:7150988*/*cyp1a*
defense response	7/91	281/14763	4.0	0.001	*lta4h*/*mpeg1*.*2*/*lyz*/*elf3*/*caspbl*/*ccl20b*/*ccl20a*.*3*
Incubation 32 °C × Challenge 24 °C Up-regulated					
response to bacterium	3/14	119/14763	26.6	<0.001	*cebpβ*/*mmp9*/*il1β*
myeloid leukocyte activation	2/14	107/14763	87.9	<0.001	*il1β*/*cxcl8b*.*1*
leukocyte chemotaxis	3/14	52/14763	60.8	0.001	*mmp13a*/*il1β*/*cxcl8b*.*1*
defense response	4/14	281/14763	15.0	<0.001	*ptgs2b*/*mmp9*/*il1β*/*cxcl8b*.*1*
response to wounding	3/14	200/14763	15.8	0.001	*mmp9*/*hbegfa*/*cxcl8b*.*1*
regeneration	2/14	137/14763	15.4	0.007	*mmp9*/*hbegfa*
Incubation 24 °C × Challenge 32 °C Up-regulated					
response to bacterium	5/129	119/14763	4.8	0.001	*lect2l*/*mmp9*/*hadhαa*/*irg1l*/ *junba*
response to external biotic stimulus	7/129	186/14763	4.3	0.004	*lect2l*/*cyp51*/*mmp9*/*hadhαa*/ *junba*/*irg1l*/*hspa5*
regeneration	7/129	137/14763	5.9	<0.001	*apoa1a*/*mvp*/*apoeb*/*mmp9*/ *hspd1*/*agr1*/*hbegfa*
response to hypoxia	4/129	45/14763	10.2	0.001	*hsp90b1*/*mb*/*igfbp1a*/*hspa5*
response to oxygen levels	4/129	46/14763	10.0	0.001	*hsp90b1*/*mb*/*igfbp1a*/*hspa5*

### Differentially expressed genes

**Figure 2:**
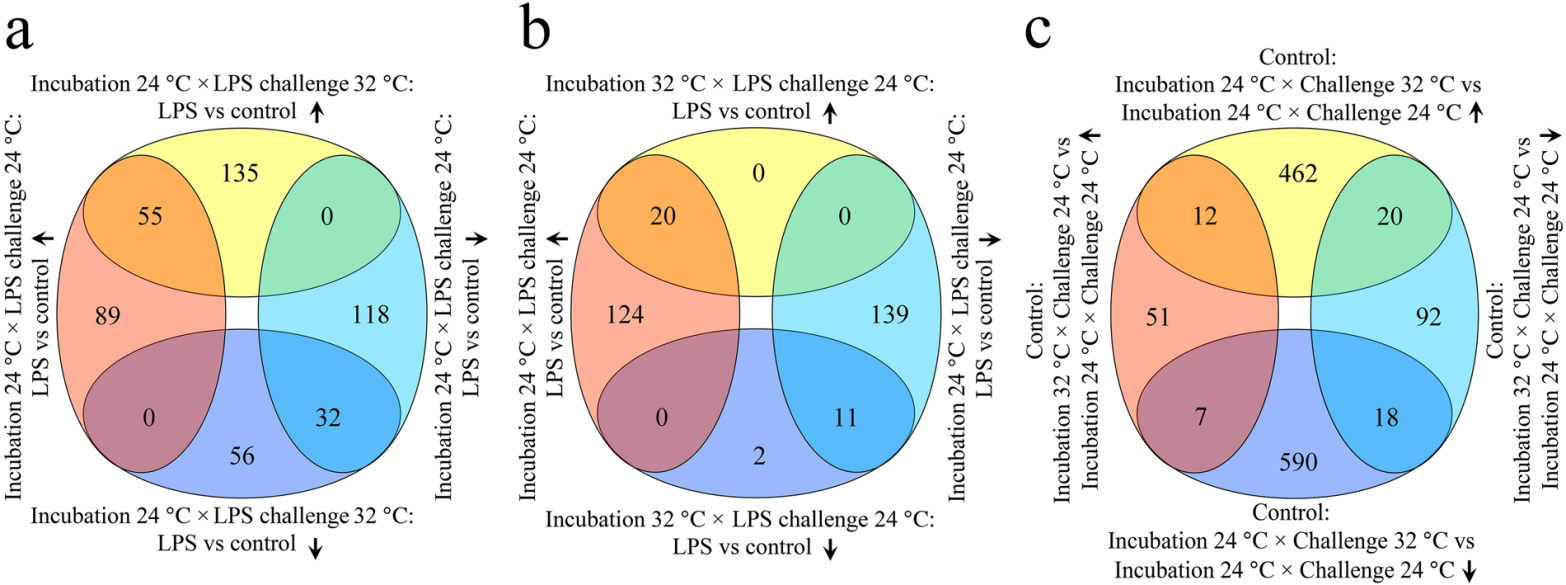
Venn diagram of differentially expressed genes. Comparison of DEGs (LPS-treated versus control) between larvae originating from the same incubation temperature of 24 °C but challenged with LPS at 24 °C or 32 °C (**a**), and between larvae originating from the 24 °C and 32 °C incubation temperatures, and challenged with LPS at the same temperature of 24 °C (**b**). DEGs with incubation and challenge temperatures in control larvae are also shown (**c**). Upward and downward arrows indicate up- and down-regulation, respectively.

A total of 605 differentially expressed genes (DEGs) (adjusted *p*-value < 0.05, fold change ≥ 1.5) were found in LPS-challenged larvae compared to their respective controls (Fig. 2, Supplementary Table S3). These included i) 294 DEGs (144 up-/150 down-regulated) in larvae incubated and challenged with LPS at 24 °C; ii) 33 DEGs (20 up-/13 down-regulated) in larvae incubated at 32 °C and challenged with LPS at 24 °C; and iii) 278 DEGs (190 up-/88 down-regulated) in larvae incubated at 24 °C and challenged with LPS at 32 °C. The comparison between LPS challenge temperatures revealed 207 DEGs (89 up-/118 down-regulated) specific to larvae challenged with LPS at 24 °C, 191 DEGs (135 up-/56 down-regulated) only in larvae challenged with LPS at 32 °C, and 87 DEGs (55 up-/32 down-regulated) shared by both groups (Fig. 2a). At the challenge temperature of 24 °C, there were 263 unique DEGs (124 up-/139 down-regulated) in larvae from the incubation temperature of 24 °C, 2 unique down-regulated DEGs in larvae incubated at 32 °C, and 31 common DEGs (20 up-/11 down-regulated) in both incubation temperature groups (Fig. 2b). A comparison between incubation and challenge temperatures identified 143 DEGs (51 up-/92 down-regulated) exclusively in control larvae incubated at 32 °C and challenged at 24 °C compared to larvae kept at constant 24 °C. A total of 1052 DEGs (462 up-/590 down-regulated) were only found in control larvae incubated at 24 °C and challenged with 32 °C compared to larvae maintained at 24 °C throughout experiment (Fig. 2c; Supplementary Table S6, S7).

**Figure 3:**
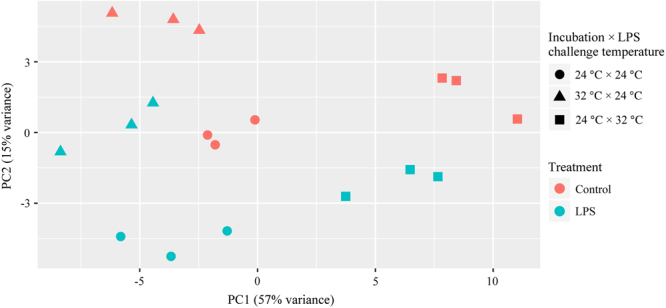
Principle component analyses of differentially expressed genes. PCA was performed on DEGs (LPS-treated versus control, adjusted *p*-value < 0.05, |fold change| ≥ 1.5) from all temperature groups. The first (PC1) and second principal components (PC2) are shown on horizontal and vertical axis, respectively.

**Figure 4:**
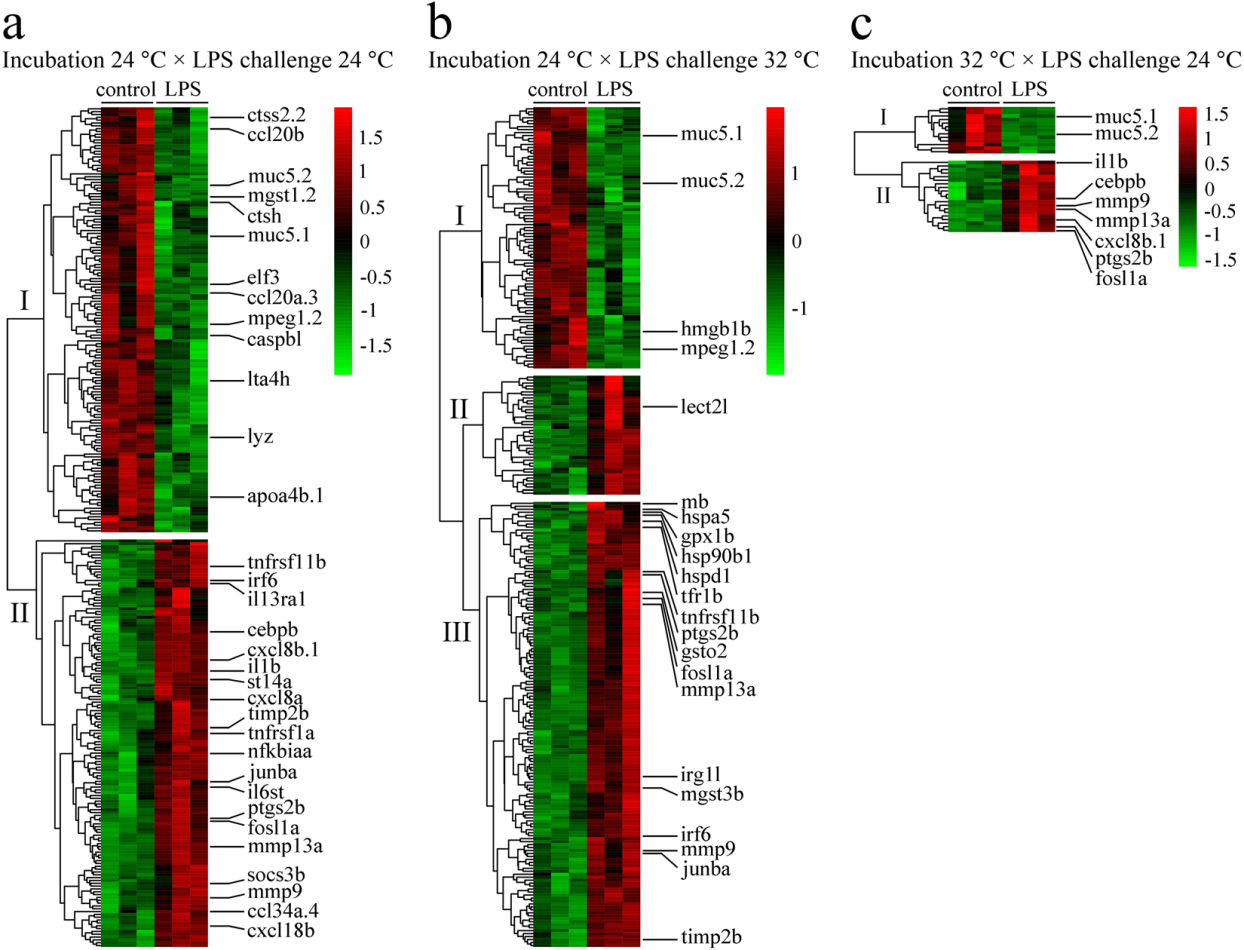
Hierarchical clustering and heat map of differentially expressed genes. Display based on DEGs (LPS-treated versus control, adjusted *p*-value < 0.05, |fold change| ≥ 1.5) for Incubation 24 °C × LPS Challenge 24 °C (**a**), Incubation 24 °C × LPS Challenge 32 °C (**b**) and Incubation 32 °C × LPS Challenge 24 °C (**c**). Log_2_ transformed gene fold change is indicated by the colour scale. Hierarchical clustering groups are shown on the vertical axis. Representative genes in each temperature group are indicated.

The principal component analysis (PCA) indicated that the first principal component (PC1) explained 57% of the total variance, while PC2 explained 15% of the total variance (Fig. 3). Moreover, a higher variance of DEGs between LPS-treated larvae and control in each temperature group was observed in PC2. Hierarchical clustering and heat maps displayed different gene expression patterns in each temperature group (Fig. 4). In larvae incubated and challenged with LPS at 24 °C, two clusters were generated, and both contained some key immune-related genes (Fig. 4a). In larvae incubated at 24 °C and challenged with LPS at 32 °C, three clusters were determined, and most immune-related genes were classified into cluster III (Fig. 4b). In larvae incubated at 32 °C and challenged with LPS at 24 °C, two clusters were identified, with the transcript levels of several key immune-related genes being up-regulated in cluster II (Fig. 4c).

### Immune processes regulated in response to LPS

**Figure 5:**
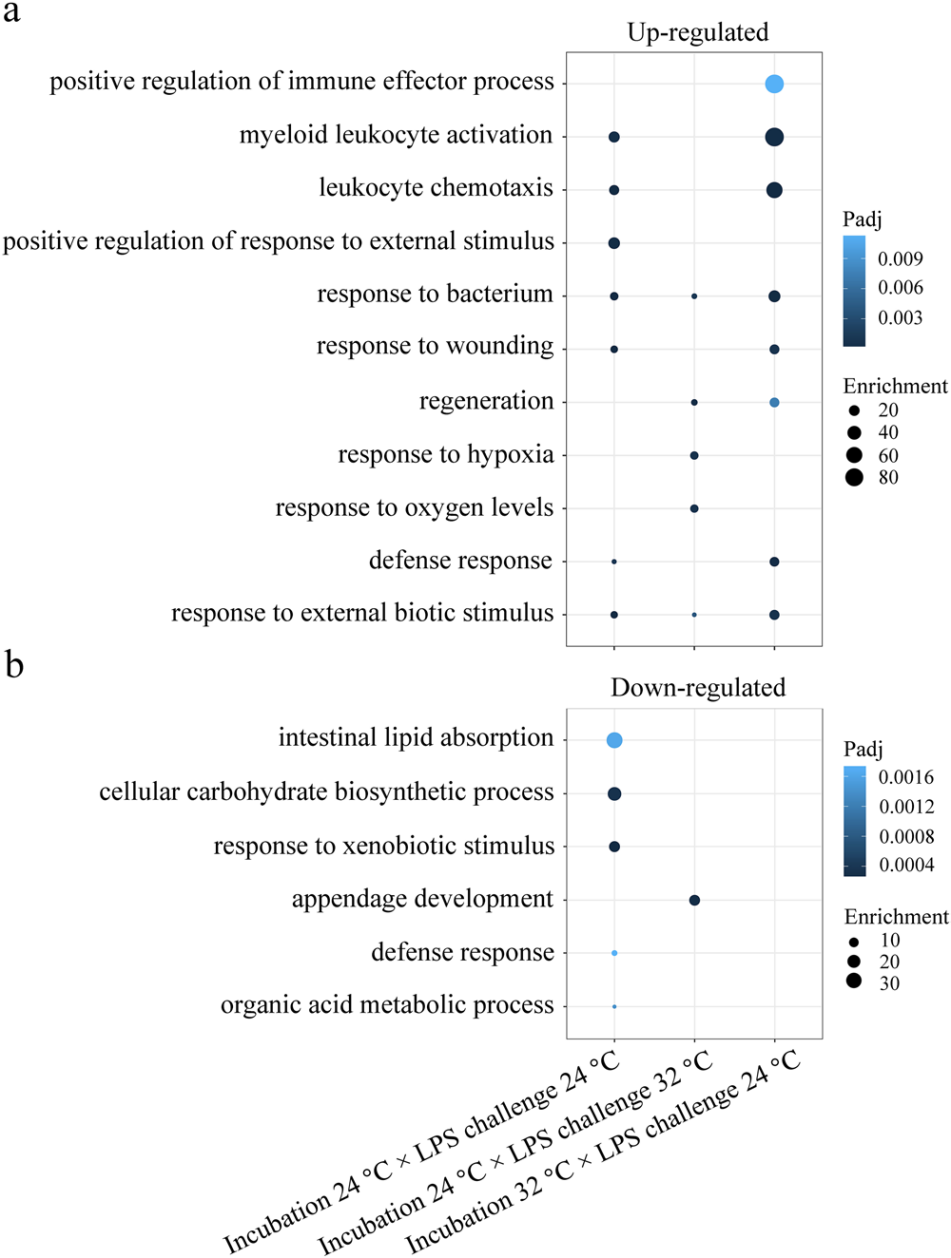
Representative GO processes of genes differentially regulated with LPS challenge. Up- (**a**) and down-regulated (**b**) GO processes in the different Incubation × LPS Challenge temperature groups are shown as dots, with size representing enrichment values (GeneRatio/BgRatio) and colour density reflecting their adjusted *p*-value. Significance was set at adjusted *p*-value < 0.05 (Benjamin-Hochberg method).

In larvae incubated and challenged with LPS at 24 °C, a number of immune processes were enriched by up-regulated DEGs, including “response to bacterium”, “myeloid leukocyte activation”, “leukocyte chemotaxis”, “defence response”, and “response to wounding” (Fig. 5a, Table 3). In contrast, the two immune processes “response to xenobiotic stimulus” and “defence response” were enriched within the down-regulated DEGs (Fig. 5b, Table 3). In larvae incubated at 32 °C and exposed to LPS at 24 °C, similar immune processes as above were enriched at even higher values by up-regulated DEGs, including two additional processes, “regeneration”, and “positive regulation of immune effector process” (Fig. 5a). No immune process was enriched by down-regulated DEGs. In larvae incubated at 24 °C and exposed to LPS at 32 °C, only three immune-related processes were stimulated compared to control, namely “response to bacterium”, “response to external biotic stimulus”, and “regeneration” (Fig. 5a, Table 3). In the same larvae group, two oxygen deficiency processes, “response to hypoxia” and “response to oxygen levels”, were enriched (Fig. 5a, Table 3). The full Gene Ontology (GO) processes are listed in Supplementary Table S4.

### KEGG pathway enrichment following LPS challenge

**Figure 6:**
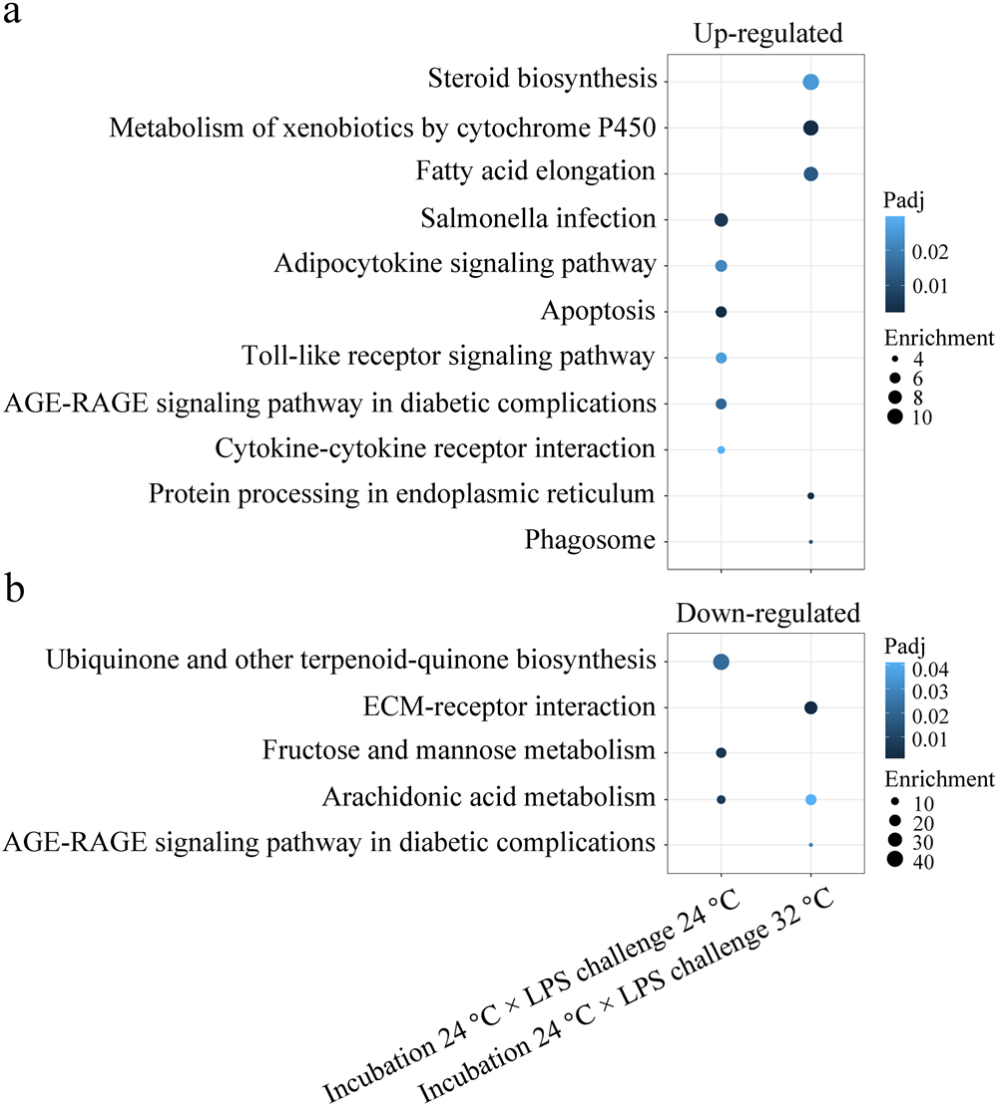
Selected KEGG pathways. KEGG pathways of up- (**a**) and down-regulated (**b**) DEGs were generated independently. Size is proportional to the enrichment value (GeneRatio/BgRatio), whereas colour density represents the adjusted *p*-value. Significance was set at adjusted *p*-value < 0.05 (Benjamin-Hochberg method).

In larvae incubated and exposed to LPS at 24 °C, pathways such as “*Salmonella* infection”, “adipocytokine signalling”, “TLR signalling”, “cytokine-cytokine receptor interaction”, and “apoptosis” were enriched by up-regulated DEGs (Fig. 6a), while “arachidonic acid metabolism” and “fructose and mannose metabolism” were enriched by down-regulated DEGs (Fig. 6b). In larvae incubated at 24 °C and challenged with LPS at 32 °C, pathways including “steroid biosynthesis”, “metabolism of xenobiotics by cytochrome P450”, “fatty acid elongation”, “protein processing in endoplasmic reticulum”, and “phagosome” were enriched by up-regulated DEGs (Fig. 6a), while “ECM-receptor interaction”, and “arachidonic acid metabolism” were enriched by down-regulated DEGs (Fig. 6b). No pathways were enriched by DEGs in larvae incubated at 32 °C and challenged with LPS at 24 °C. The full Kyoto encyclopaedia of genes and genomes (KEGG) pathways are listed in Supplementary Table S5.

### Representative immune genes involved in response to LPS

Expression of several immune-related genes was significantly regulated in LPS-treated larvae compared to control in each temperature group. In larvae incubated and challenged with LPS at 24 °C, several immune-related transcripts were up-regulated with fold-changes between 1.6 and 5.3, including cytokine *il1β* and its receptors *cxcl8a*, *cxcl8b*, *tumour necrosis factor receptor superfamily*, *member 11b* (*tnfrsf11b*), *interleukin 13 receptor*, *alpha 1* (*il13rα1*), and *interleukin 6 signal transducer* (*il6st*), pro-inflammatory mediator genes *nuclear factor of kappa light polypeptide gene enhancer in B-cells inhibitor*, *alpha a* (*nfκbiαa*), *suppressor of cytokine signaling 3b* (*socs3b*), *suppression of tumorigenicity 14 (colon carcinoma) a* (*st14a*), *fosl1a*, and *jun B proto-oncogene a* (*junba*), and chemokines *chemokine (C-C motif) ligand 34a*, *duplicate 4* (*ccl34a*.*4*) and *cxcl18b* (Table 2). Some key immune transcripts were down-regulated between 1.7 and 2.3 fold, including the antibacterial transcripts *lysozyme* (*lyz*), *macrophage expressed 1*, *tandem duplicate 2* (*mpeg1*.*2*), *cathepsin H* (*ctsh*), *cathepsin S*, *ortholog 2*, *tandem duplicate 2* (*ctss2*.*2*), and *apolipoprotein A-IV b*, *tandem duplicate 1* (*apoa4b*.*1*), pro-inflammatory transcripts *E74-like factor 3* (*elf3*), *leukotriene A4 hydrolase* (*lta4h*), and *caspase b*, *like* (*caspbl*), chemokine transcripts *ccl20a*.*3* and *ccl20b* (Table 2). In larvae incubated at 32 °C and challenged with LPS at 24 °C, transcript levels of some immune-related genes were up-regulated, including *il1β* (2.1-fold), *cxcl8b*.*1* (2.3-fold), and *CCAAT/enhancer binding protein (C/EBP)*, *beta* (*cebpβ*) (1.8-fold) (Table 2). In larvae incubated at 24 °C and challenged with LPS at 32 °C, immune transcripts such as *immunoresponsive gene 1*, *like* (*irg1l*), *TIMP metallopeptidase inhibitor 2b* (*timp2b*), *leukocyte cell-derived chemotaxin 2 like* (*lect2l*), *tnfrsf11b*, *interferon regulatory factor 6* (*irf6*), and *junba* were up-regulated between 2.1- and 6.3-fold. The expression of some heat shock protein (HSP) genes such as *heat shock 60 protein 1* (*hspd1*), *hspa5*, *hsp90b1*, and antioxidant genes such as *glutathione peroxidase 1b* (*gpx1b*), *glutathione S-transferase omega 2* (*gsto2*), *microsomal glutathione S-transferase 3b* (*mgst3b*) was enhanced 1.6–2.5 fold (Table 2). In addition, transcripts such as *matrix metallopeptidase 9* (*mmp9*), *mmp13a*, *ptgs2b*, *fosl1a*, *heparin-binding EGF-like growth factor a* (*hbegfa*), *insulin-like growth factor binding protein 1a* (*igfbp1a*), *lye*, and *anxa2a* were up-regulated, whereas *mucin 5*.*1*, *oligomeric mucus/gel-forming* (*muc5*.*1*), and *muc5*.*2* were down-regulated in all three temperature groups (Fig. 4, Table 2).

## Discussion

### Thermal developmental plasticity of innate immunity

Animals display thermal plasticity during their embryonic development, which tends to improve their performance at that particular temperature compared to that of animals exposed to other thermal conditions^16,18^. In the present study, we have shown that the survival of LPS-challenged larvae was affected by their embryonic incubation temperature. At this ontogeny stage, the adaptive immune system of zebrafish has not yet become competent, and they rely only on innate immunity for protection against pathogens^12^. The higher mortality rate of larvae originating from 24 °C embryonic incubation temperature, compared to that of larvae originating from 28 °C or 32 °C incubation temperatures, regardless of subsequent challenge temperatures, suggests that the innate immune response was negatively affected by the low incubation temperature (24 °C). In contrast, incubation at a high temperature (32 °C) had a negligible effect on the subsequent ability of first-feeding larvae to cope with LPS challenge. Low temperatures have been demonstrated to negatively influence the innate immune parameters, such as lysozyme activity^20^, respiratory burst activity^21^, opsonisation capacity^21^, blood leucocyte profiles^24^ and complement activity^21^ in adult fish. However, this cannot be generalised to all teleosts, since enhanced innate immune parameters, including blood leucocyte percentages^20^, phagocytic kidney macrophage proportion^24^, and complement activity^24^ have been observed in fish kept in low temperatures. This could be due to different properties of innate immune parameters or distinct sensitivities of different fish species to their environmental temperature, as described in Atlantic halibut strains^20^. It should be stressed that the above studies of thermal acclimation in fish were carried out at months or years post fertilization, when both the innate and adaptive immune systems were fully developed and functional. Therefore, they could not fully reflect the developmental plasticity of innate immunity during early ontogeny. Our study, focusing on the early life of zebrafish, found a negative effect of a low incubation temperature (24 °C) on the innate immune response of larvae to LPS challenge compared to 28 °C or 32 °C.

### Effect of incubation temperature on the innate immune response to LPS

**Figure 7:**
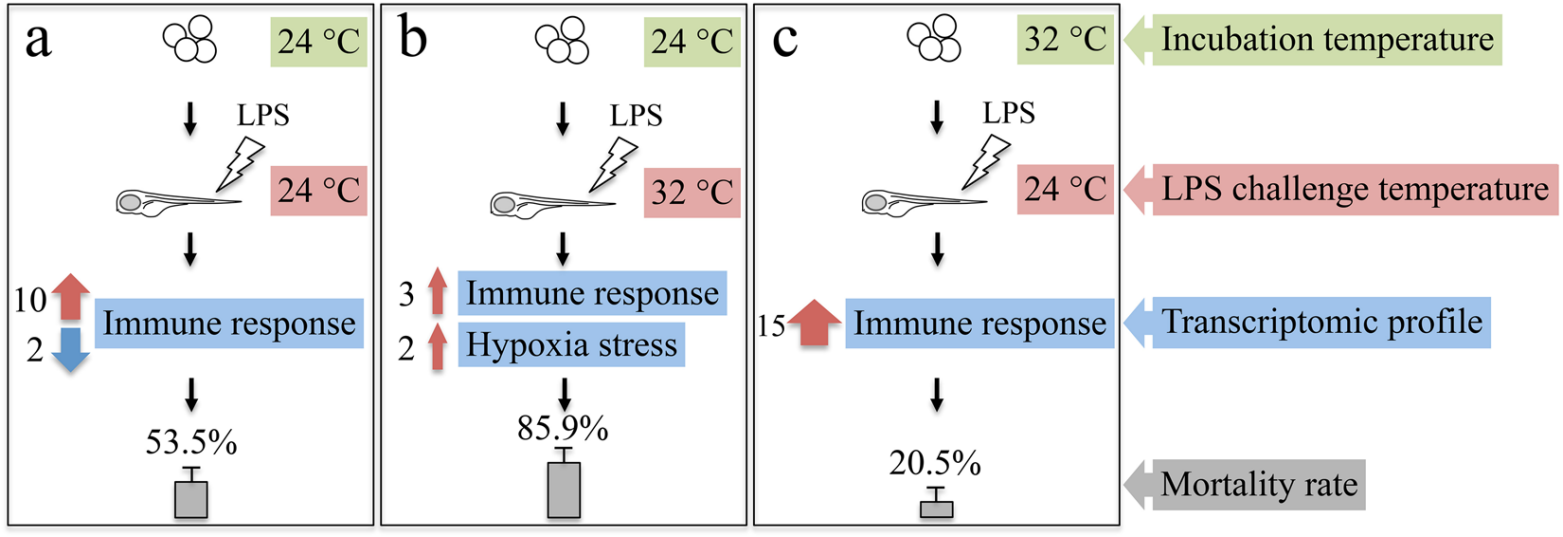
Diagram summarising the effect of incubation (24 °C, 32 °C) and challenge temperatures (24 °C, 32 °C) on the innate immune response of zebrafish larvae to LPS. Larvae incubated and challenged with LPS at 24 °C showed both up- (red arrow) and down-regulated (blue arrow) immune transcripts and processes following LPS challenge (**a**); larvae incubated at 24 °C followed by LPS exposure at 32 °C, displayed a weak immune response at the transcriptome level but additional hypoxia and stress transcripts were stimulated (**b**); an incubation temperature of 32 °C and subsequent LPS challenge at 24 °C elicited a strong immune response in larvae (**c**). The respective mortality rates are also indicated for each temperature group. The width of the arrows reflects the different numbers of immune- and hypoxia-related GO processes that are affected by LPS challenge in each temperature group. Only incubation temperatures 24 °C and 32 °C are shown, since the 28 °C group was not used for transcriptomic analyses.

In larvae incubated and exposed to LPS at 24 °C, compared to their control in the same temperature group, the pro-inflammatory response was stimulated, as suggested by the up-regulation of expression of some pro-inflammatory genes (*il1β*, *cxcl8a*, *ptgs2b*, *cebpβ*, *fosl1a*) and processes (“response to bacterium”, “myeloid leukocyte activation”, “leukocyte chemotaxis”, “defence response”, “response to wounding”). The up-regulation of the inflammatory negative mediator transcripts *nfkbiαa*^25^ and *socs3b*^26^, and the down-regulation of the pro-IL-1β processing transcript *caspbl*^27^, implies that the anti-inflammatory response could also be elicited. The anti-inflammatory response is a protective mechanism to quench excessive inflammatory signals, and to avoid pathophysiological consequences, such as sepsis^28^. Moreover, the down-regulation of antimicrobial transcripts (*lyz*, *mpeg1*.*2*, *apoa4b*.*1*, *ctsh*, *ctss2*.*2*) and immune-related processes (“response to xenobiotic stimulus”, “defence response”) indicates a decreased effectiveness of the innate immune response to LPS. Cationic lysozymes bind to negatively charged LPS at a stoichiometry lysozyme:LPS molar ratio of 1:3, resulting in the LPS structure transition from non-lamellar cube to the multilamella with reduced endotoxicity^29^. A significant drop of 2.3-fold in *lyz* expression can thereby weaken this neutralization effect. Apolipoproteins, a main group of high-density lipoproteins, neutralize LPS activity either by opsonizing its endotoxic lipid A domain or via blocking LPS-binding protein^30^. A drop (1.7-fold) in transcript levels of *apoa4b*.*1* suggests a decrease in the host capacity to neutralise endotoxic LPS. In addition, CXCL8a, CXCL8b.2, CXCL18b, and CCL34a.1 have been reported to have higher expression levels in susceptible channel catfish (*Ictalurus punctatus*) than in resistant fish when challenged with *Edwardsiella ictaluri*^31,32^. The up-regulation of *cxcl8a*, *cxcl8b*.*1*, *cxcl18b*, *ccl34a*.*4* with a fold-change between 1.7 and 2.2 may have contributed to an increased sensitivity of the larvae to LPS challenge. Both up- and down-regulated immune transcripts and processes in larvae incubated and exposed to LPS at 24 °C resulted in an intermediate mortality rate of 53.5% compared to other temperature groups (Fig. 7a).

In larvae incubated at 32 °C and exposed to LPS at 24 °C, pro-inflammatory transcripts (*il1β*, *cxcl8b*.*1*, *ptgs2b*, *cebpβ*, *fosl1a*) and processes (“response to bacterium”, “myeloid leukocyte activation”, “leukocyte chemotaxis”, “defense response”, “response to wounding”) were up-regulated in comparison to the respective controls. The regulation trends of these immune transcripts and processes were similar to those in larvae incubated and challenged with LPS at 24 °C (Table 2, Fig. 5a), and displayed even higher enrichment values of GO processes than the latter group, implying a much stronger innate immune response to LPS. This could contribute to improve the resistance of larvae to LPS in this temperature group (incubation 32 °C × challenge 24 °C) compared to their counterparts (incubation 24 °C × challenge 24 °C). Similarly, enhanced innate immune competence was observed in fish reared at high temperatures, as manifested in serum lysozyme activity^20^, complement activity^21^, respiratory burst^21^, neutrophil proportion^33^, and IFNγ signalling pathway^34^.

The change from an incubation temperature of 32 °C to the challenge temperature of 24 °C over 7 hours might have had some influence on biological processes. In common carp (*Cyprinus carpio*) that experienced cold exposure from 30 °C to either 23 °C, 17 °C or 10 °C over 1, 2, or 3 days, respectively, the expression profile of approximately 3,400 unique genes was affected^35^. To evaluate the potential effect of temperature decrease, a comparison was performed between control larvae (without LPS treatment) that experienced a temperature decrease from 32 °C to 24 °C and those kept at a constant 24 °C (Supplementary Table S6). We observed the up-regulation of transcripts of one cold-induced gene *cold inducible RNA binding protein b* (*cirbpb*) (1.6-fold) and one temperature responsive process (“response to temperature stimulus”). In particular, the nuclear receptor *nuclear receptor subfamily 1*, *group d*, *member 1* (*nr1d1*) transcripts, which code for proteins involved in both circadian and thermogenic pathways through mediation of brown adipose tissue in response to cold exposure^36^, were up-regulated 3.5-fold. It has been demonstrated that the modulation of physiological metabolism occurs to mitigate the effect of temperature decrease^37^. As expected, some HSP transcripts (*hspb associated protein 1* (*hspbap1*) and *hsp70l*) were down-regulated, as well as the antioxidant gene transcripts *cytochrome P450*, *family 24*, *subfamily A*, *polypeptide 1* (*cyp24a1*) and *gpx1a*. Nonetheless, none of these processes or transcripts were significantly regulated when the LPS treatment was taken into account, indicating that the potential effect from temperature decrease on LPS-stimulated immune response was minimal. Moreover, 10 out of 33 DEGs and all (15 out of 15) GO processes were directly or indirectly related to immunity in larvae incubated at 32 °C and challenged with LPS at 24 °C, suggesting that a more effective immune response may be elicited in larvae from the 32 °C incubation temperature group compared to their counterparts incubated during embryonic development at 24 °C. These results explain the lowest mortality rate (20.5 ± 4.7%) of larvae incubated at 32 °C and challenged with LPS at 24 °C among all the temperature groups (Fig. 7c).

### Effect of challenge temperature on the innate immune response to LPS

In larvae incubated at 24 °C and exposed to LPS at 32 °C, only three immune-related processes (“response to external biotic stimulus”, “response to bacterium”, “regeneration”) were enriched, compared to their respective controls (Fig. 5a), and none of the key cytokine genes (*tnfα*, *il1β*, *il6*) was expressed at higher levels, suggesting a limited activation of the innate immune response by LPS. Nevertheless, the abundance of transcripts of some genes with important roles in inflammatory response was changed. For instance, *irg1l* transcript levels were up-regulated 6.3-fold. Its homolog gene, *Irg1*, is inducible by LPS in mouse macrophages, and encodes cis-aconitate decarboxylase to catalyse the production of the antimicrobial itaconate^38^. IRG1 is also involved in suppressing LPS-mediated sepsis and pro-inflammatory cytokine production in mouse^39^. The up-regulation of *timp2b* (4.4-fold) could either activate the pro-inflammatory NF-κB pathway in human melanoma cells, protecting cells from apoptosis^40^, or exert the anti-inflammatory function by inhibiting NF-κB activity in murine microglial cells, to suppress the production of nitric oxide, TNFα, IL1β, and reactive oxygen species (ROS)^41^. Transcript levels of the cytokine gene, *high mobility group box 1b* (*hmgb1b*), which is involved in pro-inflammatory response^42^, necrotic cell death^43^, and sepsis^44^, were down-regulated 1.5-fold. *lect2l* was up-regulated 2.7-fold; LECT2 has a neutrophil chemotactic activity specifically in the liver^45^. The protein encoded by *hspd1* (1.7-fold up-regulation) plays a critical role in regeneration and wound healing of both hair cells and caudal fins of zebrafish larvae^46^. The oxidative stress and antioxidant response of larvae were affected as well, with the induction of hypoxia processes (“response to hypoxia”, “response to oxygen levels”), hypoxia inducible (*myoglobin* (*mb*), *igfbp1a*) and antioxidant genes (*gpx1b*, *gsto2*, *mgst3b*). In fact, the regulation of ROS and antioxidant activities by LPS has been demonstrated in zebrafish embryos^47,48^. Taken together, the limited inflammatory response and the induced hypoxia and oxidative stress could contribute jointly to the high mortality rate (85.9 ± 2.3%) of larvae incubated at 24 °C and challenged with LPS at 32 °C (Fig. 7b).

An increase in water temperature could lead to hypoxic conditions, which further promote the production of ROS, causing oxidative stress and affecting physiological activities. In control zebrafish larvae experiencing a temperature increase from 24 °C to 32 °C, transcripts of *oxidative stress responsive serine-rich 1* (*oser1*) and *reactive oxygen species modulator 1* (*romo1*) were up-regulated 1.5-fold. On the other hand, transcripts of several antioxidant genes, including *gpx1a*, *glutathione S-transferase*, *alpha tandem duplicate 1* (*gsta*.*1*), *gsto2*, *glutathione S-transferase pi 1* (*gstp1*), *gstp2*, *peroxiredoxin 6* (*prdx6*), and *NADPH oxidase organizer 1a* (*noxo1a*) were down-regulated 1.5–2.4 fold, as compared to larvae kept at constant 24 °C. We also noticed the up-regulation of HSP transcripts, such as *serpin peptidase inhibitor*, *clade H*, *member 1b* (*serpinh1b*), *hsp90aa1*.*1*, *hsp90aa1*.*2*, *crystallin*, *alpha A* (*cryaa*), *DnaJ heat shock protein family member A4* (*dnaja4*), *heat shock cognate 70* (*hsc70*), and the down-regulation of *antifreeze protein type IV* (*afp4*), and *cold inducible RNA binding protein a* (*cirbpa*) (Supplementary Table S7); this suggested that both hypoxia and antioxidant activities were elicited. A study in adult Atlantic salmon demonstrated that high temperature and oxygen deficiency affected quite similar genes and pathways related to heat shock and antioxidant responses^49^. Another report in two-banded seabream (*Diplodus vulgaris*), white seabream (*Diplodus sargus*), European seabass (*Dicentrarchus labrax*) and thinlip grey mullet (*Liza ramada*) showed that protective mechanisms, including the production of HSPs, and the antioxidant activity of glutathione S-transferase, catalase, and lipid peroxidation, can be enhanced to alleviate the effects from temperature increase and associated oxidative stress^50^. In our study, there were no significant differences in mortality rates of control larvae when the temperature changed from 24 °C to 32 °C, suggesting a limited effect of the temperature increase *per se*. Nevertheless, we cannot exclude a possible interaction between challenge temperature and LPS treatment.

### Lipopolysaccharide signalling in zebrafish

It has been demonstrated that the regulation of immune signalling pathways in response to LPS is well conserved between teleosts and mammals^51^ but alternative receptors other than TLR4 for LPS signal transduction can exist in teleosts. Some other fish-specific TLRs, such as TLR21 and TLR22, have been proposed as LPS receptor candidates^52^. However, no TLR genes showed significantly different expression in the present study. Some non-TLR receptors are also known to be involved in LPS signal transduction, such as beta-2 integrins^53^, scavenger receptor^54^, and C-type lectin^55^. GO analyses and InterPro annotation identified some up-regulated transcripts with potentially similar functions in zebrafish larvae exposed to LPS. *CD44 molecule a* (*cd44a*) codes for a protein with a C-type lectin-like domain and the genes *transmembrane protease*, *serine 4a* (*tmprss4a*) and *tmprss13b* encode scavenger receptors. The products of *proteoglycan 4b* (*prg4b*) and *integrin*, *alpha V* (*itgav*) genes display scavenger receptor and integrin activities, respectively. Further experimental evidence is needed to support their potential roles in sensing LPS.

Heat shock proteins have been implicated in LPS signal transduction. Human HSP60 contains a specific region for LPS binding^56^, while murine HSP60 was able to bind to its specific receptor on the macrophage surface independent of TLR4, but its subsequent cytokine response was dependent on TLR4^57^. Another study in Chinese hamster (*Cricetulus griseus*) ovary cells revealed that HSP70 and HSP90 were involved in sensing LPS signal from CD14 and transferring to the downstream receptors^58^. Our data showed the up-regulation of *hspd1* and *hsp90b1* in larvae incubated at 24 °C and challenged with LPS at 32 °C, suggesting their possible roles in LPS signalling. Moreover, the expression of *anxa2a* and its receptor gene *s100a10b* was up-regulated in all three temperature groups. Annexin has multiple functions, including modulation of reactive oxygen species^59^ and regulation of the inflammatory response triggered by TLR4^60^. Its new function as a TLR2 ligand was recently reported in mouse^61^. It is also noteworthy that the transcripts of *lye* were up-regulated between 2.4- and 3.6-fold in all three temperature groups. *Lye* is constitutively expressed in immune and epithelial cells^62^, with pleiotropic functions in extracellular signal transduction, phagocyte activation, and inflammatory response^63^. The direct interaction between LPS and these genes should be investigated to ascertain their involvement in LPS recognition and signalling cascade in fish.

## Conclusions

In summary, we demonstrated that both embryonic incubation and challenge temperatures affected the innate immune response to LPS in zebrafish larvae (Fig. 7). The lowest incubation temperature (24 °C) resulted in a higher mortality rate of larvae compared to the other two incubation temperatures (28 °C and 32 °C). Transcriptome analyses revealed the underlying molecular basis of this plasticity. The up-regulation of innate immune processes in response to LPS challenge was restricted in larvae originating from the lowest embryonic incubation temperature. The highest challenge temperature not only limited the immune response but also stimulated additional hypoxia and oxidative stress processes. Three genes (*anxa2a*, *s100a10b*, and *lye*), whose transcripts were up-regulated in larvae from all the temperature groups are promising receptor candidates in LPS signal transduction. These results substantially increase our understanding of the thermal plasticity of the innate immunity in zebrafish during their early development and have broader implications for fisheries and aquaculture in the context of global climate change.

## Materials and Methods

### Ethics statement

All animal procedures were conducted in compliance with the guidelines provided by the Norwegian Animal Research Authority (FOTS ID 8387) and approved by the Nord University (Norway) ethics committee.

### Fish husbandry

Zebrafish (AB strain) were maintained in a recirculating system (Aquatic Habitats, USA) under standard husbandry conditions, including a stable temperature of 28 ± 0.5 °C and photoperiod of 12 h light: 12 h dark. Adult fish were fed SDS Small Granular diet (Special Diets Services, SDS, UK) for maintenance, and SDS 400 for conditioning prior to spawning.

### Experimental design

**Figure 8:**
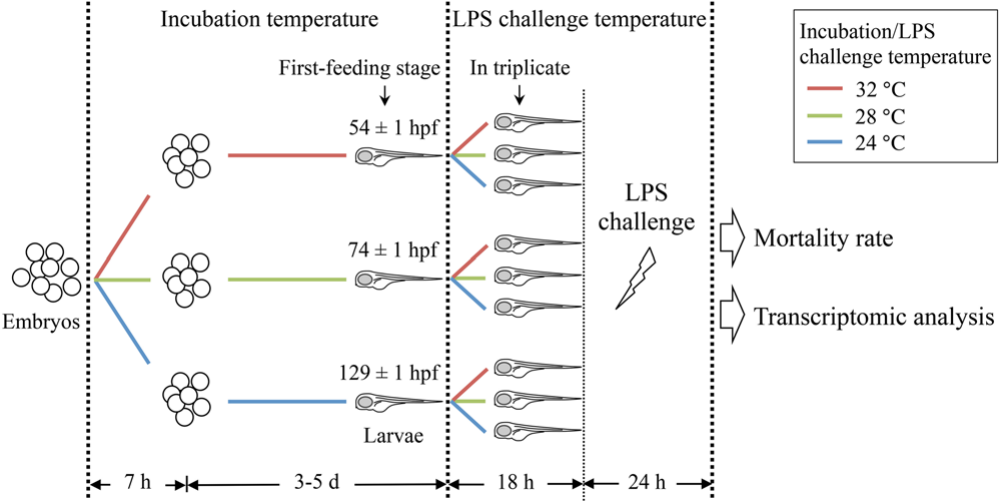
Experimental design. Zebrafish embryos obtained from spawning wild type fish maintained at 28 °C, were randomly assigned to three groups and incubated at 24 °C, 28 °C, or 32 °C (incubation temperature) throughout embryonic development. At the first-feeding stage, larvae from each incubation temperature group were divided into three new groups, followed by a temperature change to either of 24 °C, 28 °C or 32 °C (LPS challenge temperature) over 7 hours. At 18 h post the first-feeding stage, the LPS challenge was performed in all 9 temperature groups (3 incubation temperatures × 3 challenge temperatures). Mortality was evaluated at 24 h post LPS challenge. Groups exhibiting significantly different mortality rates were chosen for further transcriptomic analyses.

The experimental design is illustrated in Fig. 8. Eggs were collected in the morning two hours after first light. Approximately 3,000–4,000 eggs (2- to 64-cell stage) obtained from 10 males and 20 females were pooled and then divided into three groups with an approximately equal number in each group. The temperatures of three groups were adjusted to 24 °C, 28 °C, and 32 °C, respectively, at a rate of 0.6–0.8 °C/h. Eggs were incubated in sterile E3 medium containing 0.1 mg/L methylene blue (Sigma-Aldrich, USA) until the first-feeding stage. This standard ontogeny stage is defined as the point when the swim bladder is inflated, the mouth is protruding and larvae start to actively seek food^64^. One-third of the medium was changed daily and larvae were not fed throughout the experiment. When 75% reached the first-feeding stage (129 ± 1 hpf at 24 °C, 74 ± 1 at 28 °C, 54 ± 1 at 32 °C; Supplementary Table S1, Supplementary Fig. S1c), larvae from each incubation temperature group were further divided into the three challenge temperature groups (24 °C, 28 °C, 32 °C), and the temperature adjustments were performed as above. As shown in Supplementary Fig. S2, there were no significant differences in body length with incubation temperature (4.1 ± 0.2 mm at 24 °C, 4.0 ± 0.1 mm at 28 °C, and 4.1 ± 0.2 mm at 32 °C; mean ± s.d., n = 10). A full factorial design of three incubation temperatures and three challenge temperatures yielded nine temperature combinations. A total of 18 beakers (nine for LPS challenge and nine for control) were used. After 18 h, some larvae from each incubation × challenge temperature group were immersed in distilled water containing 10 µg/L LPS from *Pseudomonas aeruginosa* 10 (Sigma-Aldrich, USA). LPS was prepared as a stock solution at 10 mg/L in standard phosphate-buffered saline (Sigma-Aldrich, USA). The remaining individuals (controls) were immersed in distilled water containing the same dose of phosphate-buffered saline (200 µL). Larvae were kept in open 500 mL beakers immersed in fish tanks at 24 °C, 28 °C or 32 °C (challenge temperature) at a density of 149 ± 32 larva per 200 mL (n = 54, Supplementary Table S1). At the start (0 h) and 24 h post LPS challenge, mortality rates of LPS-treated and control larvae were determined in triplicate. Significant differences were evaluated by two-way analysis of variance (ANOVA) and LSD *post hoc* test with SPSS Statistics (v21.0.0.0, IBM). The ANOVA assumptions of normality and equal variance of the data were verified by Kolmogorov-Smirnov test and by Levene’s test, respectively. Statistical significance was determined at *p*-value < 0.05. Mortality rates were presented as mean ± standard deviation (s.d.). The experiment was repeated three times using randomly selected broodstock fish from the same laboratory population.

At 24 h post LPS challenge, three LPS challenge replicates and three control replicates from each of the three incubation × challenge temperature groups (incubation 24 °C × challenge 24 °C, incubation 24 °C × challenge 32 °C, incubation 32 °C × challenge 24 °C) were chosen for further transcriptomic analyses. All three incubation × challenge temperature groups showed significantly different mortality rates following LPS challenge (Fig. 1). Larvae were euthanized with 300 mg/L tricaine methanesulfonate (MS-222; Sigma-Aldrich, USA), snap-frozen in liquid nitrogen and stored at −80 °C until use. To obtain sufficient total RNA for transcriptome sequencing, each replicate was a pool of five larvae.

### Total RNA isolation, library preparation and mRNA sequencing

Samples were homogenized at 6,500 rpm for 2 × 20 s in a Precellys 24 homogenizer (Bertin Instruments, France). Total RNA was extracted from whole larvae following the QIAzol protocol (Qiagen, Germany). RNA concentration, purity and quality were determined using the NanoDrop 1000 (Thermo Scientific, USA) and the TapeStation 2200 (Agilent Technologies, USA).

TruSeq libraries were prepared from total RNA according to the manufacturer’s protocol (Illumina, USA). After purification with oligo-dT beads, mRNAs were washed and fragmented into an average length of 508–541 base pairs. The first strand of complementary DNA was synthesized with random hexamer primers (Illumina, USA), while the second strand was synthesized by Second Strand Master Mix (Illumina, USA). All 18 libraries were barcoded and normalized with the KAPA library quantification kit (Kapa Biosystems, USA). The pooled libraries were then denatured according to the NextSeq System Denature and Dilute Libraries Guide (Illumina, USA) and loaded at 11 pM on a NextSeq 500 reagent cartridge (Illumina, USA) for 150 cycle, paired-end sequencing at the Nord University genomics platform (Norway).

### Bioinformatics analyses

Raw RNA-seq data were converted to FASTQ format with bcl2fastq2 (v2.17, Illumina), followed by quality control using FastQC (https://www.bioinformatics.babraham.ac.uk/projects/fastqc/), and adapter removal using cutadapt (http://cutadapt.readthedocs.io/en/stable/guide.html) with the parameters: -q 30,25 --quality-base = 33 --trim-n -m 20. Clean reads were mapped to the zebrafish transcriptome (GRCz10.86.chr.gtf) and genome (GRCz10.dna.toplevel.fa) from Ensembl (http://www.ensembl.org) using TopHat2^65^ with parameters: -r 100 --mate-std-dev 100. Mapped reads were counted against the reference transcriptome (GRCz10.86.chr.gtf) by HTSeq-count (http://htseq.readthedocs.io/en/release_0.9.1/), and further used for differential expression analyses by DESeq2^66^ to compare LPS-treated versus control groups. DEGs were determined by DESeq2 with an adjusted *p*-value < 0.05 (Benjamin-Hochberg method). DEGs with a |fold change| ≥ 1.5 were subjected to Gene Ontology (GO) biological process and Kyoto Encyclopedia of Genes and Genomes (KEGG) pathway analyses by clusterProfiler^67^. Graphical representation was achieved using ggplot2 and pheatmap R packages.

## Electronic supplementary material

Supplementary Tables S1-S7 and Figures S1-S2
