# Supplementary material for: Low incubation temperature during early development negatively affects survival and related innate immune processes in zebrafish larvae exposed to lipopolysaccharide
Source: Sci Rep. 2018 Mar 7;8:4142. doi: 10.1038/s41598-018-22288-8 (PMC5841277; doi:10.1038/s41598-018-22288-8)

Low incubation temperature during early development negatively affects survival and related innate immune processes in zebrafish larvae exposed to lipopolysaccharide

Qirui Zhang, Martina Kopp, Igor Babiak & Jorge M.O. Fernandes*

Faculty of Biosciences and Aquaculture, Nord University, 8049 Bodø, Norway

* Correspondence and requests for materials should be addressed to J.M.O.F. (email: jorge.m.fernandes@nord.no)

Supplementary Material

Supplementary Table legends

Supplementary Table S1. Early development and mortality rate of zebrafish larvae following LPS challenge in all temperature groups. Embryo survival rate, hatching rate, hours until 75% larvae reaching first-feeding stage, body size, numbers of larvae used in experiment and mortality rates are shown. Significance was analysed using two-way ANOVA ($p < 0.05$).

Supplementary Table S2. Summary of sequenced and mapped reads from each library.

Supplementary Table S3. Full list of differentially expressed genes in LPS-challenged larvae compared to control (adjusted p-value < 0.05, Benjamin-Hochberg method, DESeq2).

Supplementary Table S4. Full list of GO processes enriched in LPS-challenged larvae compared to the corresponding untreated control (adjusted p-value < 0.05, Benjamin-Hochberg method, clusterProfiler).

Supplementary Table S5. KEGG pathways enriched in LPS-challenged larvae compared to the corresponding untreated control (adjusted p-value < 0.05, Benjamin-Hochberg method, clusterProfiler).

Supplementary Table S6. Comparison of DEGs and GO processes in control larvae between Incubation 32 °C × Challenge 24 °C and Incubation 24 °C × Challenge 24 °C.

Supplementary Table S7. Comparison of DEGs and GO processes in control larvae between Incubation 24 °C × Challenge 32 °C and Incubation 24 °C × Challenge 24 °C.

Supplementary Figure legends

Supplementary Figure S1. Early development of zebrafish at different incubation temperatures. Embryo survival (a), egg hatching rate (b), and time until 75% larvae reaching first-feeding stage (c) at different incubation temperatures are shown. The first-feeding stage is reached by 129 ± 1, 74 ± 1 and 54 ± 1 hours post-fertilization at 24 °C, 28 °C and 32 °C, respectively (mean ± S.E., n=129, 138 and 152, respectively). Superscript letters indicate significant (p-value < 0.05) differences determined by two-way ANOVA.

Supplementary Figure S2. Morphology of first feeding larvae at different incubation temperatures. (a) Morphology of first feeding larvae incubated at 24 °C (129 hpf), 28 °C (74 hpf) or 32 °C (54 hpf), and (b) respective body length (4.1 ± 0.2 mm at 24 °C, 4.0 ± 0.1 mm at 28 °C, and 4.1 ± 0.2 mm at 32 °C; mean ± s.d., n=10) are shown.

Supplementary Table S1. Early development and mortality rate of zebrafish larvae following LPS challenge in all temperature groups.

Mortality rates of LPS-challenged larvae and control at different temperatures

Thermal conditions		Mortality rate (%)									
Incubation	Challenge	Replicate 1		Replicate 2		Replicate 3		Mean		s.d.	
temperature	temperature	Control	LPS	Control	LPS	Control	LPS	Control	LPS	Control	LPS
24 °C	24 °C	0.0	48.1	0.0	51.5	0.0	61.0	0.0	53.51	0.00	3.87
24 °C	28 °C	2.5	62.9	0.0	76.1	0.0	76.9	0.8	71.93	0.85	4.54
24 °C	32 °C	1.5	82.0	0.0	85.5	2.9	90.1	1.5	85.85	0.84	2.34
28 °C	24 °C	0.0	15.4	0.0	20.0	0.0	37.7	0.0	24.35	0.00	6.79
28 °C	28 °C	1.3	58.7	0.0	43.5	0.0	49.5	0.4	50.56	0.43	4.42
28 °C	32 °C	2.0	78.3	0.0	60.5	0.0	68.3	0.7	69.02	0.68	5.16
32 °C	24 °C	0.0	11.5	1.8	27.4	0.0	22.8	0.6	20.54	0.61	4.72
32 °C	28 °C	0.9	49.2	0.0	47.7	0.0	52.2	0.3	49.69	0.30	1.33
32 °C	32 °C	2.0	60.8	5.9	72.3	1.0	65.8	1.5	66.31	1.51	3.32

The mortality rate of one control group (in red color) was identified as an outlier by Grubbs test, and was removed.

Larvae numbers used in the experiment

Replicate 1

Incubation temperature	Challenge temperature	Control				LPS			
		Dead	Survival	Total	Mortality (%)	Dead	Survival	Total	Mortality (%)
24 °C	24 °C	0	156	156	0.0	88	95	183	48.1
24 °C	28 °C	3	115	118	2.5	110	65	175	62.9
24 °C	32 °C	3	199	202	1.5	91	20	111	82.0
28 °C	24 °C	0	195	195	0.0	30	165	195	15.4
28 °C	28 °C	2	152	154	1.3	81	57	138	58.7
28 °C	32 °C	3	145	148	2.0	130	36	166	78.3
32 °C	24 °C	0	150	150	0.0	17	131	148	11.5
32 °C	28 °C	1	109	110	0.9	61	63	124	49.2
32 °C	32 °C	2	98	100	2.0	121	78	199	60.8

Replicate 2

Incubation temperature	Challenge temperature	Control				LPS			
		Dead	Survival	Total	Mortality (%)	Dead	Survival	Total	Mortality (%)
24 °C	24 °C	0	116	116	0.0	53	50	103	51.5
24 °C	28 °C	0	131	131	0.0	108	34	142	76.1
24 °C	32 °C	0	84	84	0.0	118	20	138	85.5
28 °C	24 °C	0	112	112	0.0	34	136	170	20.0
28 °C	28 °C	0	137	137	0.0	80	104	184	43.5
28 °C	32 °C	0	160	160	0.0	124	81	205	60.5
32 °C	24 °C	2	108	110	1.8	49	130	179	27.4
32 °C	28 °C	0	132	132	0.0	82	90	172	47.7
32 °C	32 °C	7	111	118	5.9	133	51	184	72.3

Replicate 3

Incubation temperature	Challenge temperature	Control				LPS			
		Dead	Survival	Total	Mortality (%)	Dead	Survival	Total	Mortality (%)
24 °C	24 °C	0	132	132	0.0	61	39	100	61.0
24 °C	28 °C	0	164	164	0.0	113	34	147	76.9
24 °C	32 °C	3	100	103	2.9	127	14	141	90.1
28 °C	24 °C	0	162	162	0.0	55	91	146	37.7
28 °C	28 °C	0	176	176	0.0	103	105	208	49.5
28 °C	32 °C	0	117	117	0.0	114	53	167	68.3
32 °C	24 °C	0	168	168	0.0	33	112	145	22.8
32 °C	28 °C	0	148	148	0.0	83	76	159	52.2
32 °C	32 °C	1	101	102	1.0	131	68	199	65.8

Survival rate of embryos incubated at different temperatures

24 ℃

	Total	Dead	Survival	Survival rate (%)
Replicate 1	132	32	100	75.8
Replicate 2	117	24	93	79.5
Replicate 3	120	30	90	75.0
Mean				76.7
S.D.				2.4

28 ℃

	Total	Dead	Survival	Survival rate (%)
Replicate 1	126	44	82	65.1
Replicate 2	116	32	84	72.4
Replicate 3	174	28	146	83.9
Mean				73.8
S.D.				9.5

32 ℃

	Total	Dead	Survival	Survival rate (%)
Replicate 1	129	30	99	76.7
Replicate 2	134	26	108	80.6
Replicate 3	120	20	100	83.3
Mean				80.2
S.D.				3.3

Hatching rate of eggs incubated at different temperatures

24 ℃

	Total	Final hatched	Final hatched %
Replicate 1	98	98	100.0
Replicate 2	126	120	95.2
Replicate 3	114	105	92.1
Mean			95.8
S.D.			4.0

28 ℃

	Total	Final hatched	Final hatched %
Replicate 1	102	100	98.0
Replicate 2	100	98	98.0
Replicate 3	111	111	100.0
Mean			98.7
S.D.			1.1

32 ℃

	Total	Final hatched	Final hatched %
Replicate 1	132	132	100.0
Replicate 2	104	104	100.0
Replicate 3	112	110	98.2
Mean			99.4
S.D.			1.0

Hours until first-feeding stage at different incubation temperatures

24 ℃

	Total	1st feeding	1st feeding %	First feeding time (h)
Replicate 1	49	37	75.5	130
Replicate 2	42	32	76.2	127.5
Replicate 3	38	29	76.3	129.5
Mean				129.0
S.D.				1.3

28 ℃

	Total			First feeding time (h)
Replicate 1	51	38	74.5	74
Replicate 2	50	38	76.0	72
Replicate 3	37	28	75.7	74.5
Mean				73.5
S.D.				1.3

32 ℃

	Total			First feeding time (h)
Replicate 1	44	33	75.0	53
Replicate 2	52	39	75.0	53.5
Replicate 3	56	42	75.0	54.5
Mean				53.7
S.D.				0.8

* The time is the hours post fertilization when 75% of larvae reached the first-feeding stage.

Supplementary Table S2. Summary of sequenced and mapped reads from each library.

Incubation x Challenge	Treatment	Replicate	Raw Reads	Trimmed Reads	Trimmed Reads (%)	≥ Q30 Reads	≥ Q30 Reads (%)	Mapped Reads	Mapped Reads (%)
24 °C x 24 °C	Control	1	29,138,294	27,993,192	96.1%	24,248,483	86.6	20,795,171	74.3
24 °C x 24 °C	Control	2	39,589,228	37,926,356	95.8%	32,266,081	85.1	27,513,112	72.5
24 °C x 24 °C	Control	3	48,009,280	46,119,584	96.1%	39,781,550	86.3	34,199,926	74.2
24 °C x 24 °C	LPS	1	38,607,716	37,020,126	95.9%	31,778,088	85.8	27,149,958	73.3
24 °C x 24 °C	LPS	2	32,606,122	31,319,710	96.1%	27,015,219	86.3	23,097,284	73.7
24 °C x 24 °C	LPS	3	13,087,100	12,494,350	95.5%	10,490,981	84.0	9,112,339	72.9
24 °C x 32 °C	Control	1	16,636,234	15,995,190	96.1%	13,842,591	86.5	12,000,553	75.0
24 °C x 32 °C	Control	2	16,462,628	15,830,164	96.2%	13,723,577	86.7	11,909,198	75.2
24 °C x 32 °C	Control	3	13,710,256	13,163,236	96.0%	11,401,416	86.6	9,886,811	75.1
24 °C x 32 °C	LPS	1	11,339,354	10,897,668	96.1%	9,344,199	85.7	8,183,262	75.1
24 °C x 32 °C	LPS	2	15,830,474	15,260,992	96.4%	13,247,552	86.8	11,573,004	75.8
24 °C x 32 °C	LPS	3	13,709,870	13,185,506	96.2%	11,463,527	86.9	9,744,808	73.9
32 °C x 24 °C	Control	1	19,012,080	18,266,678	96.1%	15,798,692	86.5	13,479,617	73.8
32 °C x 24 °C	Control	2	13,945,212	13,399,992	96.1%	11,512,240	85.9	9,927,664	74.1
32 °C x 24 °C	Control	3	13,415,120	12,885,140	96.0%	11,161,100	86.6	9,554,337	74.2
32 °C x 24 °C	LPS	1	13,706,952	13,159,532	96.0%	11,362,226	86.3	9,763,391	74.2
32 °C x 24 °C	LPS	2	14,563,068	13,992,340	96.1%	12,093,805	86.4	10,083,518	72.1
32 °C x 24 °C	LPS	3	12,885,394	12,324,942	95.7%	10,574,337	85.8	9,134,316	74.1
Sum			376,254,382	361,234,698	96.0%	311,105,664		267,108,269	

Supplementary Table S3. Full list of differentially expressed genes in LPS-challenged larvae compared to control (adjusted p-value < 0.05, Benjamin-Hochberg method, DESeq2).

Incubation 24 ℃ × Challenge 24 ℃ LPS vs control (294 DEGs)

* Adjusted *p* -value (padj) < 0.05, Benjamin-Hochberg method; |fold change| ≥ 1.5

Up-regulated (144 DEGs)

Ensembl_ID	Fold_ Change	Padj	ZFIN_Symbol	Description
ENSDARG00000012395	8.5	0.000	mmp13a	matrix metallopeptidase 13a
ENSDARG00000098700	5.3	0.000	il1b	interleukin 1, beta
ENSDARG00000010276	5.1	0.000	ptgs2b	prostaglandin-endoperoxide synthase 2b
ENSDARG00000093124	4.6	0.000	scpp8	secretory calcium-binding phosphoprotein 8
ENSDARG00000099351	3.7	0.000	igfbp1a	insulin-like growth factor binding protein 1a
ENSDARG00000015355	3.6	0.000	fosl1a	FOS-like antigen 1a
ENSDARG00000105422	3.4	0.000	si:dkey-1k23.3	si:dkey-1k23.3
ENSDARG00000100630	3.4	0.000	lye	lymphocyte antigen-6, epidermis
ENSDARG00000090352	3.4	0.000	CR855311.1	Chromosome 16: 31,668,025-31,670,760 reverse strand
ENSDARG00000073900	3.3	0.000	fam212b	family with sequence similarity 212, member B
ENSDARG00000102059	3.1	0.000	si:ch211-117l17.6	si:ch211-117l17.6
ENSDARG00000104593	3.1	0.000	cyp2k18	cytochrome P450, family 2, subfamily K, polypeptide 18
ENSDARG00000104992	3.1	0.000	epgn	epithelial mitogen homolog (mouse)
ENSDARG00000103277	2.9	0.000	cyp24a1	cytochrome P450, family 24, subfamily A, polypeptide 1
ENSDARG00000003216	2.9	0.000	anxa2a	annexin A2a
ENSDARG00000031136	2.8	0.000	moxd1	monooxygenase, DBH-like 1
ENSDARG00000040284	2.7	0.000	si:dkey-79d12.5	si:dkey-79d12.5
ENSDARG00000009544	2.7	0.000	cldnb	claudin b
ENSDARG00000007344	2.6	0.000	tcap	titin-cap (telethonin)
ENSDARG00000068493	2.5	0.000	si:zfos-411a11.2	si:zfos-411a11.2
ENSDARG00000002295	2.5	0.000	si:dkey-21p1.3	si:dkey-21p1.3
ENSDARG00000074487	2.5	0.000	CR762483.1	Chromosome 13: 30,562,119-30,564,069 forward strand
ENSDARG00000097123	2.4	0.000	FP102170.1	Chromosome 24: 38,777,319-38,782,952 reverse strand
ENSDARG00000060094	2.4	0.000	ptgis	prostaglandin I2 (prostacyclin) synthase
ENSDARG00000036135	2.4	0.000	bbox1	butyrobetaine (gamma), 2-oxoglutarate dioxygenase (gamma-butyrobetaine hydroxylase) 1
ENSDARG00000056795	2.3	0.000	serpine1	serpin peptidase inhibitor, clade E (nexin, plasminogen activator inhibitor type 1), member 1
ENSDARG00000075121	2.3	0.000	hbegfa	heparin-binding EGF-like growth factor a
ENSDARG00000091801	2.3	0.000	serpinb14	serpin peptidase inhibitor, clade B (ovalbumin), member 14
ENSDARG00000104795	2.2	0.000	cxcl8a	chemokine (C-X-C motif) ligand 8a
ENSDARG00000104133	2.2	0.000	5S_rRNA	5S ribosomal RNA
ENSDARG00000099411	2.2	0.000	zgc:158343	zgc:158343
ENSDARG00000087863	2.2	0.000	cd44a	CD44 molecule (Indian blood group) a
ENSDARG00000093381	2.1	0.000	tgm2l	transglutaminase 2, like
ENSDARG00000017799	2.1	0.000	tgm1	transglutaminase 1, K polypeptide
ENSDARG00000068288	2.1	0.000	lamc2	laminin, gamma 2
ENSDARG00000039863	2.1	0.000	lifrb	leukemia inhibitory factor receptor alpha b
ENSDARG00000075261	2.1	0.000	timp2b	TIMP metallopeptidase inhibitor 2b
ENSDARG00000002298	2.1	0.000	ankrd22	ankyrin repeat domain 22
ENSDARG00000013855	2.1	0.000	slc12a3	solute carrier family 12 (sodium/chloride transporter), member 3
ENSDARG00000089441	2.0	0.000	si:ch211-105c13.3	si:ch211-105c13.3

ENSDARG00000014047	2.0	0.000	cldn7b	claudin 7b
ENSDARG00000042816	2.0	0.001	mmp9	matrix metallopeptidase 9
ENSDARG00000099960	2.0	0.000	elovl1a	ELOVL fatty acid elongase 1a
ENSDARG00000101986	2.0	0.000	irf6	interferon regulatory factor 6
ENSDARG00000043128	2.0	0.000	cldne	claudin e
ENSDARG00000010347	2.0	0.001	acer1	alkaline ceramidase 1
ENSDARG00000105590	2.0	0.000	si:cabz01007794.1	si:cabz01007794.1
ENSDARG00000102299	2.0	0.001	cxcl8b.1	chemokine (C-X-C motif) ligand 8b, duplicate 1
ENSDARG00000002509	2.0	0.001	zgc:153911	zgc:153911
ENSDARG00000069888	2.0	0.000	cldna	claudin a
ENSDARG00000093048	1.9	0.001	si:ch211-158d24.4	si:ch211-158d24.4
ENSDARG00000042725	1.9	0.000	cebpb	CCAAT/enhancer binding protein (C/EBP), beta
ENSDARG00000017354	1.9	0.000	epha2a	eph receptor A2 a
ENSDARG00000059362	1.9	0.001	cavin1b	caveolae associated protein 1b
ENSDARG00000038025	1.9	0.000	cbx7a	chromobox homolog 7a
ENSDARG00000077069	1.9	0.000	srgn	serglycin
ENSDARG00000057644	1.9	0.000	adam8b	ADAM metallopeptidase domain 8b
ENSDARG00000071304	1.9	0.002	tbc1d2	TBC1 domain family, member 2
ENSDARG00000015709	1.9	0.000	hsd17b12a	hydroxysteroid (17-beta) dehydrogenase 12a
ENSDARG00000077573	1.9	0.002	tmprss13b	transmembrane protease, serine 13b
ENSDARG00000010752	1.9	0.000	acsl4b	acyl-CoA synthetase long-chain family member 4b
ENSDARG00000075993	1.9	0.000	clic5a	chloride intracellular channel 5a
ENSDARG00000103519	1.9	0.000	adamts1	ADAM metallopeptidase with thrombospondin type 1 motif, 1
ENSDARG00000098377	1.8	0.004	tnfrsf11b	tumor necrosis factor receptor superfamily, member 11b
ENSDARG00000013576	1.8	0.003	gadd45bb	growth arrest and DNA-damage-inducible, beta b
ENSDARG00000075045	1.8	0.007	cxcl18b	chemokine (C-X-C motif) ligand 18b
ENSDARG00000055365	1.8	0.006	si:dkey-25e12.3	si:dkey-25e12.3
ENSDARG00000059363	1.8	0.003	tgfbr2a	transforming growth factor beta receptor 2a
ENSDARG00000012789	1.8	0.009	plek2	pleckstrin 2
ENSDARG00000079372	1.8	0.010	si:ch211-264f5.6	si:ch211-264f5.6
ENSDARG00000096717	1.8	0.004	AL732488.1	Chromosome 12: 28,822,190-28,831,248 forward strand
ENSDARG00000041645	1.8	0.011	sb:cb37	sb:cb37
ENSDARG00000103117	1.8	0.000	tmprss4a	transmembrane protease, serine 4a
ENSDARG00000086815	1.8	0.002	si:dkeyp-67a8.2	si:dkeyp-67a8.2
ENSDARG00000075027	1.8	0.011	fgfr1bl	fibroblast growth factor receptor 1b, like
ENSDARG00000013711	1.8	0.001	zgc:77486	zgc:77486
ENSDARG00000077419	1.8	0.002	si:ch211-119e14.9	si:ch211-119e14.9
ENSDARG00000040623	1.8	0.000	fosl2	fos-like antigen 2
ENSDARG00000087143	1.8	0.011	serpina7	serpin peptidase inhibitor, clade A (alpha-1 antiproteinase, antitrypsin), member 7
ENSDARG00000026611	1.8	0.000	socs3b	suppressor of cytokine signaling 3b
ENSDARG00000036371	1.8	0.000	acta1a	actin, alpha 1a, skeletal muscle
ENSDARG00000077540	1.8	0.020	f2rl1.2	coagulation factor II (thrombin) receptor-like 1, tandem duplicate 2
ENSDARG00000097208	1.8	0.000	alkbh3	si:ch1073-281m9.1
ENSDARG00000097137	1.8	0.000	CR753876.1	Chromosome 19: 35,781,482-35,782,717 forward strand
ENSDARG00000104773	1.7	0.000	junbb	jun B proto-oncogene b
ENSDARG00000102750	1.7	0.000	cdh1	cadherin 1, type 1, E-cadherin (epithelial)
ENSDARG00000010255	1.7	0.011	bin2b	bridging integrator 2b
ENSDARG00000005673	1.7	0.012	f3b	coagulation factor IIIb
ENSDARG00000038424	1.7	0.024	si:dkey-8k3.2	si:dkey-8k3.2

ENSDARG00000100968	1.7	0.002	si:ch211-1a19.3	si:ch211-1a19.3
ENSDARG00000076839	1.7	0.008	ftr86	finTRIM family, member 86
ENSDARG00000035852	1.7	0.013	cart3	cocaine- and amphetamine-regulated transcript 3
ENSDARG00000058992	1.7	0.015	cers2b	ceramide synthase 2b
ENSDARG00000054616	1.7	0.000	cldni	claudin i
ENSDARG00000100088	1.7	0.001	zmp:0000001114	zmp:0000001114
ENSDARG00000090873	1.7	0.022	ccl34a.4	chemokine (C-C motif) ligand 34a, duplicate 4
ENSDARG00000028096	1.7	0.000	si:ch211-95j8.2	si:ch211-95j8.2
ENSDARG00000070571	1.7	0.008	zgc:153953	zgc:153953
ENSDARG00000013415	1.7	0.004	lmna	lamin A
ENSDARG00000089543	1.7	0.015	CU984579.1	Chromosome 5: 36,888,191-36,896,815 forward strand
ENSDARG00000069377	1.7	0.039	si:dkey-242g16.2	si:dkey-242g16.2
ENSDARG00000028396	1.7	0.000	fkbp5	FK506 binding protein 5
ENSDARG00000025403	1.7	0.000	ftr83	finTRIM family, member 83
ENSDARG00000089156	1.7	0.014	egr3	early growth response 3
ENSDARG00000035858	1.7	0.000	cnn2	calponin 2
ENSDARG00000089124	1.7	0.038	hbae1.3	hemoglobin, alpha embryonic 1.3
ENSDARG00000104511	1.7	0.046	SCARNA2	Small Cajal body-specific RNA 2
ENSDARG00000088040	1.7	0.025	si:dkeyp-27c8.2	si:dkeyp-27c8.2
ENSDARG00000070950	1.7	0.049	si:dkey-14o18.6	si:dkey-14o18.6
ENSDARG00000038742	1.7	0.001	si:ch211-119o8.7	si:ch211-119o8.7
ENSDARG00000089086	1.6	0.038	mustn1b	musculoskeletal, embryonic nuclear protein 1b
ENSDARG00000037746	1.6	0.000	actb1	actin, beta 1
ENSDARG00000061173	1.6	0.000	st14a	suppression of tumorigenicity 14 (colon carcinoma) a
ENSDARG00000042874	1.6	0.002	phlda2	pleckstrin homology-like domain, family A, member 2
ENSDARG00000016691	1.6	0.000	cd9b	CD9 molecule b
ENSDARG00000074378	1.6	0.001	junba	jun B proto-oncogene a
ENSDARG00000033466	1.6	0.000	tagln2	transgelin 2
ENSDARG00000099195	1.6	0.004	ier2a	immediate early response 2a
ENSDARG00000037421	1.6	0.000	egr1	early growth response 1
ENSDARG00000039142	1.6	0.000	arpc5a	actin related protein 2/3 complex, subunit 5A
ENSDARG00000104693	1.6	0.002	il6st	interleukin 6 signal transducer
ENSDARG00000005481	1.6	0.007	nfkbiaa	nuclear factor of kappa light polypeptide gene enhancer in B-cells inhibitor, alpha a
ENSDARG00000100339	1.6	0.020	arf4b	ADP-ribosylation factor 4b
ENSDARG00000070052	1.6	0.004	tdrd6	tudor domain containing 6
ENSDARG00000061081	1.6	0.009	arpp21	cAMP-regulated phosphoprotein, 21
ENSDARG00000099385	1.6	0.003	CT030188.1	Chromosome 7: 39,139,162-39,169,054 forward strand
ENSDARG00000092115	1.6	0.000	eif4a1a	eukaryotic translation initiation factor 4A1A
ENSDARG00000061242	1.6	0.002	tuft1a	tuftelin 1a
ENSDARG00000009133	1.6	0.011	myo1eb	myosin IE, b
ENSDARG00000080020	1.6	0.037	il13ra1	interleukin 13 receptor, alpha 1
ENSDARG00000031683	1.5	0.034	fosab	v-fos FBJ murine osteosarcoma viral oncogene homolog Ab
ENSDARG00000086098	1.5	0.025	errfi1b	ERBB receptor feedback inhibitor 1b
ENSDARG00000098853	1.5	0.002	ehd1a	EH-domain containing 1a
ENSDARG00000059906	1.5	0.000	sdc4	syndecan 4
ENSDARG00000068589	1.5	0.000	zmp:0000000719	zmp:0000000719
ENSDARG00000036074	1.5	0.014	cebpa	CCAAT/enhancer binding protein (C/EBP), alpha
ENSDARG00000041051	1.5	0.013	mid1ip1a	MID1 interacting protein 1a
ENSDARG00000025254	1.5	0.000	s100a10b	S100 calcium binding protein A10b
ENSDARG00000003808	1.5	0.000	aqp3a	aquaporin 3a

ENSDARG00000017320	1.5	0.002	f11r.1	F11 receptor, tandem duplicate 1
ENSDARG00000018569	1.5	0.031	tnfrsf1a	tumor necrosis factor receptor superfamily, member 1a
ENSDARG00000070427	1.5	0.007	s100v1	S100 calcium binding protein V1
ENSDARG00000091699	1.5	0.003	capn2a	calpain 2, (m/II) large subunit a
ENSDARG00000103032	1.5	0.015	rap2b	RAP2B, member of RAS oncogene family

Down-regulated (150 DEGs)

Ensembl_ID	Fold_ Change	Padj	ZFIN_Symbol	Description
ENSDARG00000058556	-5.5	0.000	muc5.2	mucin 5.2
ENSDARG00000070331	-3.6	0.000	muc5.1	mucin 5.1, oligomeric mucus/gel-forming
ENSDARG00000076830	-3.4	0.000	si:dkey-65b12.6	si:dkey-65b12.6
ENSDARG00000105341	-3.3	0.000	si:dkey-9l20.3	si:dkey-9l20.3
ENSDARG00000101473	-2.8	0.000	si:ch211-25g7.5	si:ch211-25g7.5
ENSDARG00000076192	-2.8	0.000	ankrd1b	ankyrin repeat domain 1b (cardiac muscle)
ENSDARG00000056873	-2.7	0.000	and3	actinodin3
ENSDARG00000023082	-2.7	0.000	krt1-19d	keratin, type 1, gene 19d
ENSDARG00000028098	-2.7	0.000	fut9d	fucosyltransferase 9d
ENSDARG00000090526	-2.7	0.000	zgc:158404	zgc:158404
ENSDARG00000003203	-2.7	0.000	rhcga	Rh family, C glycoprotein a
ENSDARG00000054753	-2.6	0.000	col10a1a	collagen, type X, alpha 1a
ENSDARG00000053761	-2.5	0.000	si:dkey-187j14.4	si:dkey-187j14.4
ENSDARG00000092730	-2.4	0.000	si:dkey-22i16.3	si:dkey-22i16.3
ENSDARG00000091996	-2.4	0.000	si:ch211-117m20.5	si:ch211-117m20.5
ENSDARG00000079227	-2.4	0.000	plekhs1	pleckstrin homology domain containing, family S member 1
ENSDARG00000097416	-2.4	0.000	si:ch73-306e8.2	si:ch73-306e8.2
ENSDARG00000068951	-2.3	0.000	si:ch211-219a15.4	si:ch211-219a15.4
ENSDARG00000057789	-2.3	0.000	lyz	lysozyme
ENSDARG00000088514	-2.3	0.000	and1	actinodin1
ENSDARG00000032801	-2.3	0.000	grk5	G protein-coupled receptor kinase 5
ENSDARG00000027088	-2.2	0.000	ptgdsb.1	prostaglandin D2 synthase b, tandem duplicate 1
ENSDARG00000052779	-2.2	0.000	zgc:153932	zgc:153932
ENSDARG00000093365	-2.2	0.000	si:ch211-226h7.3	si:ch211-226h7.3
ENSDARG00000101040	-2.1	0.000	ccl20a.3	chemokine (C-C motif) ligand 20a, duplicate 3
ENSDARG00000103199	-2.1	0.000	si:dkey-247k7.2	si:dkey-247k7.2
ENSDARG00000070041	-2.1	0.000	zgc:153920	zgc:153920
ENSDARG00000073936	-2.1	0.000	BX511021.2	Chromosome 3: 31,997,770-31,999,326 forward strand
ENSDARG00000021366	-2.1	0.000	fbp1a	fructose-1,6-bisphosphatase 1a
ENSDARG00000062221	-2.1	0.000	si:ch211-135f11.1	si:ch211-135f11.1
ENSDARG00000038185	-2.1	0.000	gh1	growth hormone 1
ENSDARG00000092704	-2.1	0.000	zmp:0000001031	zmp:0000001031
ENSDARG00000102548	-2.1	0.000	CR735107.1	Chromosome 12: 44,514,875-44,519,222 reverse strand
ENSDARG00000009153	-2.1	0.000	pla2g1b	phospholipase A2, group IB (pancreas)
ENSDARG00000069559	-2.1	0.001	muc13a	mucin 13a, cell surface associated
ENSDARG00000076623	-2.1	0.000	col14a1b	collagen, type XIV, alpha 1b
ENSDARG00000094210	-2.1	0.000	zgc:109934	zgc:109934
ENSDARG00000102435	-2.0	0.000	plekhf1	pleckstrin homology domain containing, family F (with FYVE domain) member 1
ENSDARG00000104642	-2.0	0.000	si:dkey-201i2.1	si:dkey-201i2.1
ENSDARG00000091757	-2.0	0.000	adgrf6	adhesion G protein-coupled receptor F6
ENSDARG00000036830	-2.0	0.000	krt17	keratin 17
ENSDARG00000095863	-2.0	0.001	afp4	antifreeze protein type IV

ENSDARG00000055514	-2.0	0.000	icn2	ictacalcin 2
ENSDARG00000101816	-2.0	0.002	col5a3b	collagen, type V, alpha 3b
ENSDARG00000092719	-2.0	0.002	AL954655.2	Chromosome 12: 46,103,512-46,121,565 reverse strand
ENSDARG00000098058	-2.0	0.001	im:7150988	im:7150988
ENSDARG00000099581	-1.9	0.001	txnbb	tenascin XBb
ENSDARG00000078832	-1.9	0.002	si:dkey-73p2.3	si:dkey-73p2.3
ENSDARG00000055504	-1.9	0.000	si:ch211-212k18.7	si:ch211-212k18.7
ENSDARG00000074642	-1.9	0.000	zgc:153932	zgc:153932
ENSDARG00000007024	-1.9	0.002	uox	urate oxidase
ENSDARG00000100190	-1.9	0.001	si:ch211-188p14.4	si:ch211-188p14.4
ENSDARG00000104359	-1.9	0.000	anxa1c	annexin A1c
ENSDARG00000094951	-1.9	0.003	BX072532.1	Chromosome 12: 46,386,343-46,402,220 reverse strand
ENSDARG00000094511	-1.9	0.002	ccl20b	chemokine (C-C motif) ligand 20b
ENSDARG00000097826	-1.9	0.003	si:dkey-239b22.2	si:dkey-239b22.2
ENSDARG00000069415	-1.9	0.000	col17a1a	collagen, type XVII, alpha 1a
ENSDARG00000045592	-1.9	0.001	tnni2a.1	troponin I type 2a (skeletal, fast), tandem duplicate 1
ENSDARG00000102442	-1.9	0.001	zgc:165502	zgc:165502
ENSDARG00000091715	-1.9	0.006	si:dkey-162h11.2	si:dkey-162h11.2
ENSDARG00000044512	-1.9	0.003	ca4c	carbonic anhydrase IV c
ENSDARG00000096712	-1.9	0.001	si:dkey-193p11.2	si:dkey-193p11.2
ENSDARG00000097157	-1.9	0.005	si:ch211-207n23.2	si:ch211-207n23.2
ENSDARG00000039684	-1.9	0.001	sirt5	sirtuin 5
ENSDARG00000068181	-1.8	0.004	dpep1	dipeptidase 1 (renal)
ENSDARG00000099448	-1.8	0.000	sh3d21	SH3 domain containing 21
ENSDARG00000044094	-1.8	0.010	gfpt2	glutamine-fructose-6-phosphate transaminase 2
ENSDARG00000094433	-1.8	0.007	caspbl	caspase b, like
ENSDARG00000076146	-1.8	0.011	zgc:172075	zgc:172075
ENSDARG00000030896	-1.8	0.008	foxq1a	forkhead box Q1a
ENSDARG00000040683	-1.8	0.005	si:ch73-40a17.3	si:ch73-40a17.3
ENSDARG00000020364	-1.8	0.000	fbp1b	fructose-1,6-bisphosphatase 1b
ENSDARG00000098315	-1.8	0.011	cyp1a	cytochrome P450, family 1, subfamily A
ENSDARG00000095888	-1.8	0.016	BX248517.1	Chromosome 16: 29,581,348-29,595,739 reverse strand
ENSDARG00000104216	-1.8	0.014	CU929368.1	Chromosome 8: 235,035-243,867 forward strand
ENSDARG00000037748	-1.8	0.002	slc43a1b	solute carrier family 43 (amino acid system L transporter), member 1b
ENSDARG00000098197	-1.8	0.006	CU633999.1	Chromosome 19: 60,788-64,099 reverse strand
ENSDARG00000020956	-1.8	0.000	pck2	phosphoenolpyruvate carboxykinase 2 (mitochondrial)
ENSDARG00000101199	-1.8	0.000	rbp4	retinol binding protein 4, plasma
ENSDARG00000036840	-1.8	0.000	krt15	keratin 15
ENSDARG00000040747	-1.7	0.000	tm4sf4	transmembrane 4 L six family member 4
ENSDARG00000095147	-1.7	0.006	krt96	keratin 96
ENSDARG00000079745	-1.7	0.000	si:ch211-166a6.5	si:ch211-166a6.5
ENSDARG00000052437	-1.7	0.001	mia	melanoma inhibitory activity
ENSDARG00000044935	-1.7	0.000	hpdb	4-hydroxyphenylpyruvate dioxygenase b
ENSDARG00000090899	-1.7	0.023	sycn.3	syncollin, tandem duplicate 3
ENSDARG00000076221	-1.7	0.020	zgc:198419	zgc:198419
ENSDARG00000079175	-1.7	0.001	si:ch211-79k12.1	si:ch211-79k12.1
ENSDARG00000100075	-1.7	0.001	abcg2a	ATP-binding cassette, sub-family G (WHITE), member 2a
ENSDARG00000078622	-1.7	0.028	scpp5	secretory calcium-binding phosphoprotein 5

ENSDARG00000096445	-1.7	0.009	si:ch211-214p16.3	si:ch211-214p16.3
ENSDARG00000098011	-1.7	0.022	BX000438.2	Chromosome 15: 31,042,185-31,043,291 forward strand
ENSDARG00000079302	-1.7	0.000	and2	actinodin2
ENSDARG00000077982	-1.7	0.002	elf3	E74-like factor 3 (ets domain transcription factor, epithelial-specific)
ENSDARG00000013771	-1.7	0.008	ctss2.2	cathepsin S, ortholog 2, tandem duplicate 2
ENSDARG00000070000	-1.7	0.000	txnipb	thioredoxin interacting protein b
ENSDARG00000097738	-1.7	0.000	AL929222.2	Chromosome 3: 23,089,722-23,107,761 reverse strand
ENSDARG00000041108	-1.7	0.000	ctsh	cathepsin H
ENSDARG00000043093	-1.7	0.034	mpeg1.2	macrophage expressed 1, tandem duplicate 2
ENSDARG00000021720	-1.7	0.006	col7a1	collagen, type VII, alpha 1
ENSDARG00000095388	-1.7	0.039	BX323590.2	Chromosome 1: 18,996,812-19,001,783 reverse strand
ENSDARG00000038359	-1.7	0.002	enosf1	enolase superfamily member 1
ENSDARG00000087574	-1.7	0.040	nox1	NADPH oxidase 1
ENSDARG00000098997	-1.7	0.023	BX001050.2	Chromosome 10: 40,892,716-40,893,360 forward strand
ENSDARG00000012405	-1.7	0.000	col1a1a	collagen, type I, alpha 1a
ENSDARG00000029747	-1.7	0.028	mep1a.1	meprin A, alpha (PABA peptide hydrolase), tandem duplicate 1
ENSDARG00000040298	-1.7	0.018	apoa4b.1	apolipoprotein A-IV b, tandem duplicate 1
ENSDARG00000010478	-1.7	0.003	hsp90aa1.1	heat shock protein 90, alpha (cytosolic), class A member 1, tandem duplicate 1
ENSDARG00000076862	-1.7	0.050	fam198a	family with sequence similarity 198, member A
ENSDARG00000069381	-1.7	0.036	si:ch211-125e6.5	si:ch211-125e6.5
ENSDARG00000018351	-1.7	0.001	hpda	4-hydroxyphenylpyruvate dioxygenase a
ENSDARG00000093936	-1.7	0.014	si:dkeyp-1h4.6	si:dkeyp-1h4.6
ENSDARG00000005799	-1.7	0.011	smim14	small integral membrane protein 14
ENSDARG00000098239	-1.7	0.012	zgc:85932	zgc:85932
ENSDARG00000043806	-1.7	0.014	postna	periostin, osteoblast specific factor a
ENSDARG00000070480	-1.6	0.007	agr2	anterior gradient 2
ENSDARG00000104200	-1.6	0.022	BX901923.2	Chromosome 15: 17,950,176-18,000,106 forward strand
ENSDARG00000001993	-1.6	0.001	myhb	myosin, heavy chain b
ENSDARG00000053625	-1.6	0.006	anxa2b	annexin A2b
ENSDARG00000006427	-1.6	0.000	fabp2	fatty acid binding protein 2, intestinal
ENSDARG00000006840	-1.6	0.006	gnmt	glycine N-methyltransferase
ENSDARG00000067958	-1.6	0.018	sh3gl1a	SH3-domain GRB2-like 1a
ENSDARG00000053554	-1.6	0.025	wdr76	WD repeat domain 76
ENSDARG00000056314	-1.6	0.036	a2ml	alpha-2-macroglobulin-like
ENSDARG00000097513	-1.6	0.049	CT573383.1	Chromosome 25: 33,550,114-33,564,975 forward strand
ENSDARG00000088366	-1.6	0.011	zgc:77938	zgc:77938
ENSDARG00000103785	-1.6	0.013	sult2st2	sulfotransferase family 2, cytosolic sulfotransferase 2
ENSDARG00000012881	-1.6	0.014	slc4a1a	solute carrier family 4 (anion exchanger), member 1a (Diego blood group)
ENSDARG00000055539	-1.6	0.001	epdl2	ependymin-like 2
ENSDARG00000019498	-1.6	0.015	cry5	cryptochrome circadian clock 5
ENSDARG00000020143	-1.6	0.004	pah	phenylalanine hydroxylase
ENSDARG00000058799	-1.6	0.027	mybpha	myosin binding protein Ha
ENSDARG00000012729	-1.6	0.009	hcls1	hematopoietic cell-specific Lyn substrate 1
ENSDARG00000053684	-1.6	0.002	aldob	aldolase b, fructose-bisphosphate
ENSDARG00000053483	-1.5	0.012	zgc:113054	zgc:113054

ENSDARG00000017985	-1.5	0.045	zgc:77739	zgc:77739
ENSDARG00000019096	-1.5	0.036	myl7	myosin, light chain 7, regulatory
ENSDARG00000068374	-1.5	0.049	si:ch211-132b12.7	si:ch211-132b12.7
ENSDARG00000103687	-1.5	0.011	sycn.2	syncollin, tandem duplicate 2
ENSDARG00000103659	-1.5	0.050	bco1l	beta-carotene oxygenase 1, like
ENSDARG00000103878	-1.5	0.031	anpepb	alanyl (membrane) aminopeptidase b
ENSDARG00000039269	-1.5	0.004	arg2	arginase 2
ENSDARG00000018263	-1.5	0.012	pdia2	protein disulfide isomerase family A, member 2
ENSDARG00000070057	-1.5	0.030	si:dkey-69o16.5	si:dkey-69o16.5
ENSDARG00000020007	-1.5	0.000	col1a2	collagen, type I, alpha 2
ENSDARG00000022165	-1.5	0.009	mgst1.2	microsomal glutathione S-transferase 1.2
ENSDARG00000006029	-1.5	0.032	lta4h	leukotriene A4 hydrolase
ENSDARG00000004658	-1.5	0.014	zgc:101810	zgc:101810
ENSDARG00000009443	-1.5	0.037	zgc:92137	zgc:92137
ENSDARG00000056781	-1.5	0.009	zanl	zonadhesin, like

Supplementary Table S4. Full list of GO processes enriched in LPS-challenged larvae compared to the corresponding untreated control.

Incubation 24 °C × Challenge 24 °C

* Adjusted *p*-value (padj) < 0.05, Benjamin-Hochberg method

* Enrichment = GeneRatio/BgRatio

Up-regulated

GO ID	Description	Gene Ratio	BgRatio	Enrichment	Padj	Gene Name	Count
GO:0009617	response to bacterium	7/84	119/14763	10.3	0.001	tnfrsf1a/cebpb/mmp9/junba/cxcl18b/il1b/junbb	7
GO:0043207	response to external biotic stimulus	8/84	186/14763	7.6	0.001	nfkbiaa/tnfrsf1a/cebpb/mmp9/junba/cxcl18b/il1b/junbb	8
GO:0032103	positive regulation of response to external stimulus	3/84	21/14763	25.1	0.005	mmp9/cxcl18b/il6st	3
GO:0002274	myeloid leukocyte activation	3/84	24/14763	22.0	0.007	cxcl18b/il1b/cxcl8b.1	3
GO:0097529	myeloid leukocyte migration	5/84	49/14763	17.9	0.001	mmp13a/cxcl18b/il1b/cxcl8b.1/cxcl8a	5
GO:0030595	leukocyte chemotaxis	5/84	52/14763	16.9	0.001	mmp13a/cxcl18b/il1b/cxcl8b.1/cxcl8a	5
GO:0016477	cell migration	10/84	467/14763	3.8	0.007	mmp13a/cnn2/cebpa/mmp9/sdc4/cxcl18b/il1b/cxcl8b.1/cdh1/cxcl8a	10
GO:0048870	cell motility	10/84	493/14763	3.6	0.009	mmp13a/cnn2/cebpa/mmp9/sdc4/cxcl18b/il1b/cxcl8b.1/cdh1/cxcl8a	10
GO:0006952	defense response	7/84	281/14763	4.4	0.017	ptgs2b/tnfrsf1a/mmp9/cxcl18b/il1b/cxcl8b.1/cxcl8a	7
GO:0009611	response to wounding	9/84	200/14763	7.9	0.001	f3b/mmp9/sdc4/hbegfa/f2rl1.2/cxcl8b.1/il6st/junbb/cxcl8a	9
GO:1901700	response to oxygen-containing compound	6/84	229/14763	4.6	0.026	tnfrsf1a/socs3b/junba/cxcl18b/il1b/junbb	6
GO:0042127	regulation of cell proliferation	6/84	237/14763	4.4	0.028	tnfrsf1a/junba/cxcl18b/hbegfa/igfbp1a/junbb	6
GO:0044255	cellular lipid metabolic process	8/84	411/14763	3.4	0.028	ptgs2b/acer1/hsd17b12a/si:ch211-119o8.7/cers2b/ptgis/elovl1a/cyp2k18	8
GO:0090130	tissue migration	3/84	49/14763	10.8	0.033	cnn2/acta1a/cdh1	3

Down-regulated

GO ID	Description	Gene Ratio	BgRatio	Enrichment	Padj	Gene Name	Count
GO:0009410	response to xenobiotic stimulus	4/91	49/14763	13.2	0.018	foxq1a/si:ch211-117m20.5/im:7150988/cyp1a	4
GO:0006952	defense response	7/91	281/14763	4.0	0.042	lta4h/mpeg1.2/lyz/elf3/caspbl/ccl20b/ccl20a.3	7
GO:0044712	single-organism catabolic process	10/91	283/14763	5.7	0.005	uox/pla2g1b/hpda/pah/fbp1b/fbp1a/gh1/enosf1/aldob/afp4	10
GO:0034637	cellular carbohydrate biosynthetic process	3/91	22/14763	22.1	0.019	fbp1b/fbp1a/si:ch211-219a15.4	3
GO:0006082	organic acid metabolic process	9/91	433/14763	3.4	0.042	lta4h/uox/hpda/pah/enosf1/arg2/hpdb/aldob/afp4	9
GO:0098856	intestinal lipid absorption	2/91	10/14763	32.4	0.042	anxa2b/afp4	2

Incubation 24 ℃ × Challenge 32 ℃

* Adjusted *p*-value (padj) < 0.05, Benjamin-Hochberg method

* Enrichment = GeneRatio/BgRatio

Up-regulated

GO ID	Description	GeneRatio	BgRatio	Enrichment	Padj	Gene Name	Count
GO:0043207	response to external biotic stimulus	7/129	186/14763	4.3	0.020	lect2l/cyp51/mmp9/hadhaa/irg1l/junba/hspa5	7
GO:0009617	response to bacterium	5/129	119/14763	4.8	0.046	lect2l/mmp9/hadhaa/irg1l/junba	5
GO:0031099	regeneration	7/129	137/14763	5.8	0.005	apoa1a/mvp/apoeb/mmp9/hspd1/agr1/hbegfa	7
GO:0001666	response to hypoxia	4/129	45/14763	10.2	0.013	hsp90b1/mb/igfbp1a/hspa5	4
GO:0070482	response to oxygen levels	4/129	46/14763	10.0	0.013	hsp90b1/mb/igfbp1a/hspa5	4
GO:0003014	renal system process	3/129	22/14763	15.6	0.016	myo1eb/anln/myh9a	3
GO:0035383	thioester metabolic process	3/129	37/14763	9.3	0.046	elovl7a/elovl1a/elovl1b	3
GO:0097006	regulation of plasma lipoprotein particle levels	2/129	10/14763	22.9	0.045	apoa1a/apoeb	2
GO:0044255	cellular lipid metabolic process	14/129	411/14763	3.9	0.001	ptgs2b/acer1/apoa1a/hsd17b12a/apoeb/degs2/msmo1/hadhaa/elovl7a/fa2h/elovl1a/hmgcs1/elovl1b/cyp2k18	14
GO:0006082	organic acid metabolic process	13/129	433/14763	3.4	0.003	ugt1ab/odc1/ptgs2b/apoa1a/hsd17b12a/idh1/msmo1/hadhaa/elovl7a/fa2h/elovl1a/elovl1b/cyp2k18	13
GO:0045454	cell redox homeostasis	4/129	56/14763	8.2	0.022	pdia4/glrx/txn/pdia3	4
GO:1901615	organic hydroxy compound metabolic process	5/129	115/14763	5.0	0.045	apoa1a/apoeb/degs2/cyp51/msmo1	5

Down-regulated

GO ID	Description	GeneRatio	BgRatio	Enrichment	Padj	Gene Name	Count
GO:0048736	appendage development	4/51	90/14763	12.9	0.038	col2a1b/col1a1a/and2/and1	4

Incubation 32 °C × Challenge 24 °C

* Adjusted *p*-value (padj) < 0.05, Benjamin-Hochberg method

* Enrichment = GeneRatio/BgRatio

Up-regulated

GO ID	Description	Gene Ratio	BgRatio	Enrichment	Padj	Gene Name	Count
GO:0009617	response to bacterium	3/14	119/14763	26.6	0.003	cebpb/mmp9/il1b	3
GO:0043207	response to external biotic stimulus	3/14	186/14763	17.0	0.005	cebpb/mmp9/il1b	3
GO:0001775	cell activation	2/14	107/14763	19.7	0.020	il1b/cxcl8b.1	2
GO:0002274	myeloid leukocyte activation	2/14	24/14763	87.9	0.003	il1b/cxcl8b.1	2
GO:0097529	myeloid leukocyte migration	3/14	49/14763	64.6	0.001	mmp13a/il1b/cxcl8b.1	3
GO:0030595	leukocyte chemotaxis	3/14	52/14763	60.8	0.001	mmp13a/il1b/cxcl8b.1	3
GO:0016477	cell migration	4/14	467/14763	9.0	0.005	mmp13a/mmp9/il1b/cxcl8b.1	4
GO:0048870	cell motility	4/14	493/14763	8.6	0.005	mmp13a/mmp9/il1b/cxcl8b.1	4
GO:0006935	chemotaxis	3/14	265/14763	11.9	0.010	mmp13a/il1b/cxcl8b.1	3
GO:0042330	taxis	3/14	278/14763	11.4	0.011	mmp13a/il1b/cxcl8b.1	3
GO:0006952	defense response	4/14	281/14763	15.0	0.002	ptgs2b/mmp9/il1b/cxcl8b.1	4
GO:0009611	response to wounding	3/14	200/14763	15.8	0.005	mmp9/hbegfa/cxcl8b.1	3
GO:0042246	tissue regeneration	2/14	93/14763	22.7	0.016	mmp9/hbegfa	2
GO:0031099	regeneration	2/14	137/14763	15.4	0.030	mmp9/hbegfa	2
GO:0002699	positive regulation of immune effector process	1/14	12/14763	87.9	0.039	mmp9	1

Supplementary Table S5. KEGG pathways enriched in LPS-challenged larvae compared to the corresponding untreated control.

Incubation 24 ℃ × Challenge 24 ℃

* Adjusted *p*-value (padj) < 0.05, Benjamin-Hochberg method

* Enrichment = GeneRatio/BgRatio

Up-regulated

KEGG ID	Description	Gene Ratio	BgRatio	Enrichment	Padj	Gene Name	Count
dre04514	Cell adhesion molecules (CAMs)	7/37	139/5659	7.7	0.001	cldnb/cldn7b/f11r.1/cldne/sdc4/cldna/cdh1	7
dre04210	Apoptosis	7/37	174/5659	6.2	0.002	nfkbiaa/lmna/gadd45bb/tnfrsf1a/fosab/actb1/capn2a	7
dre05132	Salmonella infection	5/37	97/5659	7.9	0.006	fosab/actb1/arpc5a/si:dkey-181m9.8/cxcl8a	5
dre04933	AGE-RAGE signaling pathway in diabetic complications	5/37	128/5659	6.0	0.015	f3b/egr1/serpine1/si:dkey-181m9.8/cxcl8a	5
dre04530	Tight junction	6/37	217/5659	4.2	0.022	cldnb/cldn7b/f11r.1/actb1/cldne/cldna	6
dre04920	Adipocytokine signaling pathway	4/37	92/5659	6.6	0.022	nfkbiaa/acsl4b/tnfrsf1a/socs3b	4
dre04620	Toll-like receptor signaling pathway	4/37	101/5659	6.1	0.026	nfkbiaa/fosab/si:dkey-181m9.8/cxcl8a	4
dre04060	Cytokine-cytokine receptor interaction	5/37	175/5659	4.4	0.029	tnfrsf1a/il13ra1/si:dkey-181m9.8/il6st/cxcl8a	5
dre05168	Herpes simplex infection	5/37	187/5659	4.1	0.034	nfkbiaa/tnfrsf1a/socs3b/fosab/si:dkey-181m9.8	5

Down-regulated

KEGG ID	Description	Gene Ratio	BgRatio	Enrichment	Padj	Gene Name	Count
dre00360	Phenylalanine metabolism	3/41	16/5659	25.9	0.006	hpda/pah/hpdb	3
dre00051	Fructose and mannose metabolism	4/41	43/5659	12.8	0.006	fbp1b/fbp1a/enosf1/aldob	4
dre00590	Arachidonic acid metabolism	4/41	51/5659	10.8	0.007	lta4h/pla2g1b/ptgdsb.1/si:ch211-214p16.3	4
dre00030	Pentose phosphate pathway	3/41	33/5659	12.5	0.019	fbp1b/fbp1a/aldob	3
dre00010	Glycolysis / Gluconeogenesis	4/41	75/5659	7.4	0.019	fbp1b/pck2/fbp1a/aldob	4
dre00130	Ubiquinone and other terpenoid-quinone biosynthesis	2/41	11/5659	25.1	0.022	hpda/hpdb	2
dre00592	alpha-Linolenic acid metabolism	2/41	18/5659	15.3	0.050	pla2g1b/si:ch211-214p16.3	2

Incubation 24 ℃ × Challenge 32 ℃

* Adjusted *p*-value (padj) < 0.05, Benjamin-Hochberg method

* Enrichment value = GeneRatio/BgRatio

Up-regulated

KEGG ID	Description	Gene Ratio	BgRatio	Enrichment	Padj	Gene Name	Count
dre04530	Tight junction	12/76	217/5659	4.1	0.002	oclna/tuba8l4/actn1/cldnb/cldn7b/f11r.1/actb1/cdc42l/tuba8l/cldne/myh9a/cldna	12
dre00982	Drug metabolism - cytochrome P450	5/76	36/5659	10.3	0.003	ugt1ab/ugt1ab/aldh3b1/gsto2/mgst3b	5
dre04141	Protein processing in endoplasmic reticulum	10/76	181/5659	4.1	0.003	hsp90b1/pdia4/ddost/canx/bcap31/capn2a/pdia3/calr3b/hspa5/calr3a	10
dre00980	Metabolism of xenobiotics by cytochrome P450	5/76	39/5659	9.5	0.003	ugt1ab/ugt1ab/aldh3b1/gsto2/mgst3b	5
dre04514	Cell adhesion molecules (CAMs)	8/76	139/5659	4.3	0.008	oclna/itgav/cldnb/cldn7b/f11r.1/uck1/cldna/cdh1	8
dre00062	Fatty acid elongation	4/76	34/5659	8.8	0.013	hsd17b12a/hadhaa/elovl7a/elovl1b	4
dre04145	Phagosome	8/76	158/5659	3.8	0.013	tuba8l4/itgav/cyba/canx/actb1/tuba8l/calr3b/calr3a	8
dre00100	Steroid biosynthesis	3/76	21/5659	10.6	0.025	cyp51/msmo1/sqlea	3

Down-regulated

KEGG ID	Description	Gene Ratio	BgRatio	Enrichment	Padj	Gene Name	Count
dre04512	ECM-receptor interaction	4/16	81/5659	17.5	0.002	col2a1b/col1a1a/col1a2/col1a1b	4
dre00360	Phenylalanine metabolism	2/16	16/5659	44.2	0.012	pah/hpdb	2
dre04510	Focal adhesion	4/16	246/5659	5.8	0.029	col2a1b/col1a1a/col1a2/col1a1b	4
dre04744	Phototransduction	2/16	37/5659	19.1	0.029	gnat2/calm3a	2
dre04933	AGE-RAGE signaling pathway in diabetic complications	3/16	128/5659	8.3	0.029	col1a1a/col1a2/col1a1b	3
dre00590	Arachidonic acid metabolism	2/16	51/5659	13.9	0.041	ptgdsb.1/ptgdsb.2	2

Supplementary Table S6. Comparison of DEGs and GO processes in control larvae between Incubation 32 °C × Challenge 24 °C and Incubation 24 °C × Challenge 24 °C.

Control: Incubation 32 ℃ × Challenge 24 ℃ vs Incubation 24 ℃ × Challenge 24 ℃ (200 DEGs)

* Adjusted *p*-value (padj) < 0.05, Benjamin-Hochberg method; |fold change| ≥ 1.5

Up-regulated (70 DEGs)

Ensembl_ID	Fold_ Change	Padj	ZFIN_Symbol	Description
ENSDARG00000102364	4.0	0.000	si:dkey-202l22.6	si:dkey-202l22.6
ENSDARG00000033160	3.5	0.000	nr1d1	nuclear receptor subfamily 1, group d, member 1
ENSDARG00000056885	3.0	0.000	per1a	period circadian clock 1a
ENSDARG00000039099	3.0	0.000	aep1	aerolysin-like protein
ENSDARG00000088589	2.5	0.000	ponzr3	plac8 onzin related protein 3
ENSDARG00000058094	2.4	0.000	ciarta	circadian associated repressor of transcription a
ENSDARG00000003203	2.4	0.001	rhcga	Rh family, C glycoprotein a
ENSDARG00000007382	2.4	0.000	ubtd1a	ubiquitin domain containing 1a
ENSDARG00000103016	2.3	0.002	si:ch211-180m24.1	si:ch211-180m24.1
ENSDARG00000057652	2.2	0.000	dbpb	D site albumin promoter binding protein b
ENSDARG00000006008	2.2	0.000	dct	dopachrome tautomerase
ENSDARG00000100288	2.1	0.002	impg2b	interphotoreceptor matrix proteoglycan 2b
ENSDARG00000102403	2.1	0.000	cry2	cryptochrome circadian clock 2
ENSDARG00000041691	2.1	0.000	bhlhe41	basic helix-loop-helix family, member e41
ENSDARG00000055638	2.0	0.021	ankrd33aa	ankyrin repeat domain 33Aa
ENSDARG00000088171	2.0	0.021	ciartb	circadian associated repressor of transcription b
ENSDARG00000087873	2.0	0.000	eevs	2-epi-5-epi-valiolone synthase
ENSDARG00000020602	2.0	0.000	grk7a	G protein-coupled receptor kinase 7a
ENSDARG00000007480	2.0	0.000	rpe65a	retinal pigment epithelium-specific protein 65a
ENSDARG00000033760	2.0	0.021	pmelb	premelanosome protein b
ENSDARG00000069377	1.9	0.044	si:dkey-242g16.2	si:dkey-242g16.2
ENSDARG00000053315	1.9	0.036	tmprss3a	transmembrane protease, serine 3a
ENSDARG00000076554	1.9	0.037	cdkn1a	cyclin-dependent kinase inhibitor 1A
ENSDARG00000012499	1.9	0.000	per1b	period circadian clock 1b
ENSDARG00000094280	1.9	0.007	usp21	ubiquitin specific peptidase 21
ENSDARG00000063014	1.9	0.000	dbpa	D site albumin promoter binding protein a
ENSDARG00000005023	1.9	0.005	fkbp9	FK506 binding protein 9
ENSDARG00000019949	1.9	0.047	serpinh1b	serpin peptidase inhibitor, clade H (heat shock protein 47), member 1b
ENSDARG00000053509	1.9	0.009	kazald3	Kazal-type serine peptidase inhibitor domain 3
ENSDARG00000070157	1.8	0.007	tgm2a	transglutaminase 2, C polypeptide A
ENSDARG00000012126	1.8	0.035	zgc:109965	zgc:109965
ENSDARG00000026771	1.8	0.012	tmem41ab	transmembrane protein 41ab
ENSDARG00000045737	1.8	0.033	guca1g	guanylate cyclase activator 1g
ENSDARG00000097445	1.8	0.033	si:dkey-195m11.8	si:dkey-195m11.8
ENSDARG00000077652	1.8	0.019	zgc:194355	zgc:194355
ENSDARG00000101289	1.8	0.004	slc29a1a	solute carrier family 29 (equilibrative nucleoside transporter), member 1a
ENSDARG00000078095	1.7	0.003	cipcb	CLOCK-interacting pacemaker b
ENSDARG00000101597	1.7	0.049	si:dkeyp-57d7.4	si:dkeyp-57d7.4
ENSDARG00000096242	1.7	0.028	si:ch211-76l23.4	si:ch211-76l23.4

ENSDARG00000077388	1.7	0.015	obsl1b	obscurin-like 1b
ENSDARG00000038658	1.7	0.004	murca	muscle-related coiled-coil protein a
ENSDARG00000098646	1.7	0.006	mthfd2	methylenetetrahydrofolate dehydrogenase (NADP+ dependent) 2, methenyltetrahydrofolate cyclohydrolase
ENSDARG00000038785	1.7	0.025	abcf2a	ATP-binding cassette, sub-family F (GCN20), member 2a
ENSDARG00000075942	1.7	0.004	si:ch211-69m14.1	si:ch211-69m14.1
ENSDARG00000028396	1.7	0.040	fkbp5	FK506 binding protein 5
ENSDARG00000077236	1.7	0.018	hspb6	heat shock protein, alpha-crystallin-related, b6
ENSDARG00000057427	1.7	0.004	sv2ba	synaptic vesicle glycoprotein 2Ba
ENSDARG00000055118	1.7	0.044	mylipb	myosin regulatory light chain interacting protein b
ENSDARG00000070107	1.6	0.010	six7	SIX homeobox 7
ENSDARG00000074396	1.6	0.046	fscn2b	fascin actin-bundling protein 2b, retinal
ENSDARG00000057683	1.6	0.014	mcm6	minichromosome maintenance complex component 6
ENSDARG00000068374	1.6	0.043	si:ch211-132b12.7	si:ch211-132b12.7
ENSDARG00000092115	1.6	0.002	eif4a1a	eukaryotic translation initiation factor 4A1A
ENSDARG00000091298	1.6	0.006	pmela	premelanosome protein a
ENSDARG00000056151	1.6	0.014	tyrp1b	tyrosinase-related protein 1b
ENSDARG00000006900	1.6	0.036	impdh2	IMP (inosine 5'-monophosphate) dehydrogenase 2
ENSDARG00000036832	1.6	0.036	cyt1l	type I cytokeratin, enveloping layer, like
ENSDARG00000056743	1.6	0.002	murcb	muscle-related coiled-coil protein b
ENSDARG00000075161	1.6	0.009	defbl1	defensin, beta-like 1
ENSDARG00000040334	1.6	0.001	mat2aa	methionine adenosyltransferase II, alpha a
ENSDARG00000013351	1.6	0.000	cirbpb	cold inducible RNA binding protein b
ENSDARG00000102798	1.6	0.022	mcm2	minichromosome maintenance complex component 2
ENSDARG00000060457	1.5	0.006	pmp22b	peripheral myelin protein 22b
ENSDARG00000104353	1.5	0.012	nop58	NOP58 ribonucleoprotein homolog (yeast)
ENSDARG00000024561	1.5	0.043	nolc1	nucleolar and coiled-body phosphoprotein 1
ENSDARG00000042322	1.5	0.043	si:dkey-177p2.6	si:dkey-177p2.6
ENSDARG00000044299	1.5	0.010	lmnb1	lamin B1
ENSDARG00000008732	1.5	0.012	zgc:66479	zgc:66479
ENSDARG00000036811	1.5	0.049	ifrd2	interferon-related developmental regulator 2
ENSDARG00000007141	1.5	0.008	psmc3	proteasome 26S subunit, ATPase 3

Down-regulated (130 DEGs)

Ensembl_ID	Fold_ Change	Padj	ZFIN_Symbol	Description
ENSDARG00000042816	-3.8	0.000	mmp9	matrix metallopeptidase 9
ENSDARG00000045835	-3.3	0.000	si:dkey-14d8.6	si:dkey-14d8.6
ENSDARG00000096445	-3.3	0.000	si:ch211-214p16.3	si:ch211-214p16.3
ENSDARG00000017780	-2.9	0.000	rorcb	RAR-related orphan receptor C b
ENSDARG00000061858	-2.8	0.000	zgc:153968	zgc:153968
ENSDARG00000100795	-2.8	0.000	timp4a	TIMP metallopeptidase inhibitor 4a
ENSDARG00000040683	-2.8	0.000	si:ch73-40a17.3	si:ch73-40a17.3
ENSDARG00000091131	-2.7	0.000	cry1bb	cryptochrome circadian clock 1bb
ENSDARG00000016391	-2.7	0.000	calcoco1b	transcription elongation factor B (SIII), polypeptide 3

ENSDARG00000010420	-2.6	0.000	ndrg1b	N-myc downstream regulated 1b
ENSDARG00000015355	-2.6	0.000	fosl1a	FOS-like antigen 1a
ENSDARG00000030915	-2.5	0.000	cpa1	carboxypeptidase A1 (pancreatic)
ENSDARG00000038643	-2.4	0.000	alas2	aminolevulinate, delta-, synthase 2
ENSDARG00000101051	-2.4	0.000	ctsbb	cathepsin Bb
ENSDARG00000073936	-2.3	0.000		ferritin, middle subunit-like
ENSDARG00000100504	-2.3	0.002	ppp1r27b	protein phosphatase 1, regulatory subunit 27b
ENSDARG00000017490	-2.3	0.001	cel.1	carboxyl ester lipase, tandem duplicate 1
ENSDARG00000029587	-2.2	0.000	msra	methionine sulfoxide reductase A
ENSDARG00000042725	-2.2	0.000	cebpb	CCAAT/enhancer binding protein (C/EBP), beta
ENSDARG00000041433	-2.2	0.006	si:dkey-7c18.24	si:dkey-7c18.24
ENSDARG00000036028	-2.2	0.000	arrdc3b	arrestin domain containing 3b
ENSDARG00000055539	-2.2	0.001	epdl2	ependymin-like 2
ENSDARG00000079347	-2.1	0.001	zgc:194659	zgc:194659
ENSDARG00000096739	-2.1	0.010	si:dkey-219e21.2	si:dkey-219e21.2
ENSDARG00000077360	-2.1	0.010	zgc:173593	zgc:173593
ENSDARG00000044566	-2.1	0.001	fabp6	fatty acid binding protein 6, ileal (gastrotropin)
ENSDARG00000039547	-2.1	0.013	si:ch211-113a14.22	si:ch211-113a14.22
ENSDARG00000074613	-2.1	0.013	si:ch211-240l19.6	si:ch211-240l19.6
ENSDARG00000022466	-2.1	0.005	clcn5b	chloride channel, voltage-sensitive 5b
ENSDARG00000076563	-2.1	0.013	zgc:92249	zgc:92249
ENSDARG00000053526	-2.1	0.013	enpp7.1	ectonucleotide pyrophosphatase/phosphodiesterase 7, tandem duplicate 1
ENSDARG00000094557	-2.0	0.001	nupr1	nuclear protein 1
ENSDARG00000097533	-2.0	0.000	si:dkey-29l4.4	si:dkey-29l4.4
ENSDARG00000035519	-2.0	0.000	histh1l	histone H1 like
ENSDARG00000102059	-2.0	0.024	si:ch211-117l17.6	si:ch211-117l17.6
ENSDARG00000038559	-2.0	0.000	h1f0	H1 histone family, member 0
ENSDARG00000023759	-2.0	0.004	zgc:73226	zgc:73226
ENSDARG00000021339	-2.0	0.005	cpa5	carboxypeptidase A5
ENSDARG00000103498	-2.0	0.000	epd	ependymin
ENSDARG00000103277	-2.0	0.022	cyp24a1	cytochrome P450, family 24, subfamily A, polypeptide 1
ENSDARG00000080337	-2.0	0.000	AC024175.4	AC024175.4
ENSDARG00000104687	-2.0	0.004	slc16a9b	solute carrier family 16, member 9b
ENSDARG00000018351	-2.0	0.002	hpda	4-hydroxyphenylpyruvate dioxygenase a
ENSDARG00000023820	-2.0	0.002	faxdc2	fatty acid hydroxylase domain containing 2
ENSDARG00000043719	-2.0	0.036	c3a.6	complement component c3a, duplicate 6
ENSDARG00000055723	-2.0	0.035	hsp70l	heat shock cognate 70-kd protein, like
ENSDARG00000070000	-2.0	0.000	txnipb	thioredoxin interacting protein b
ENSDARG00000074345	-1.9	0.043	si:ch211-214b16.4	si:ch211-214b16.3
ENSDARG00000069630	-1.9	0.010	tat	tyrosine aminotransferase
ENSDARG00000094073	-1.9	0.043		CR381686.3
ENSDARG00000069074	-1.9	0.000	cry1ba	cryptochrome circadian clock 1ba
ENSDARG00000102593	-1.9	0.047	si:ch211-214b16.2	si:ch211-214b16.2
ENSDARG00000033854	-1.9	0.021	abrab	actin binding Rho activating protein b

ENSDARG00000103659	-1.9	0.018	bco1l	beta-carotene oxygenase 1, like
ENSDARG00000092033	-1.9	0.010	si:dkey-239h2.3	si:dkey-239h2.3
ENSDARG00000039730	-1.9	0.013	zgc:112160	zgc:112160
ENSDARG00000102907	-1.9	0.022	CABZ01072043.1	CABZ01072043.1
ENSDARG00000001953	-1.9	0.018	pfkfb3	6-phosphofructo-2-kinase/fructose-2,6-biphosphatase 3
ENSDARG00000087911	-1.9	0.012	psme4a	proteasome activator subunit 4a
ENSDARG00000095633	-1.9	0.026	si:ch211-133l5.7	si:ch211-133l5.7
ENSDARG00000032469	-1.9	0.010	ampd3b	adenosine monophosphate deaminase 3b
ENSDARG00000019532	-1.9	0.033	fads2	fatty acid desaturase 2
ENSDARG00000033771	-1.9	0.015	hspbap1	hspb associated protein 1
ENSDARG00000105408	-1.8	0.002	si:ch211-237b12.4	si:ch211-237b12.4
ENSDARG00000102558	-1.8	0.000	pde6h	phosphodiesterase 6H, cGMP-specific, cone, gamma
ENSDARG00000104037	-1.8	0.001	si:dkey-74k8.4	si:dkey-74k8.4
ENSDARG00000099519	-1.8	0.001	zgc:66475	zgc:66475
ENSDARG00000003281	-1.8	0.001	pik3ip1	phosphoinositide-3-kinase interacting protein 1
ENSDARG00000075727	-1.8	0.007	map1lc3cl	microtubule-associated protein 1 light chain 3 gamma, like
ENSDARG00000037551	-1.8	0.049	pm20d1.1	peptidase M20 domain containing 1, tandem duplicate 1
ENSDARG00000092870	-1.8	0.000	si:dkey-100n23.4	si:dkey-100n23.4
ENSDARG00000052690	-1.8	0.004	arrdc3a	arrestin domain containing 3a
ENSDARG00000042112	-1.8	0.038	dio1	deiodinase, iodothyronine, type I
ENSDARG00000035810	-1.8	0.002	rgcc	regulator of cell cycle
ENSDARG00000104929	-1.8	0.018	si:ch211-153f2.7	si:ch211-153f2.7
ENSDARG00000005085	-1.8	0.000	ggctb	gamma-glutamylcyclotransferase b
ENSDARG00000038153	-1.8	0.022	lgals2b	lectin, galactoside-binding, soluble, 2b
ENSDARG00000070057	-1.8	0.050	si:dkey-69o16.5	si:dkey-69o16.5
ENSDARG00000055595	-1.8	0.043	clul1	clusterin-like 1 (retinal)
ENSDARG00000012881	-1.8	0.016	slc4a1a	solute carrier family 4 (anion exchanger), member 1a (Diego blood group)
ENSDARG00000022832	-1.8	0.040	bnip4	BCL2/adenovirus E1B interacting protein 4
ENSDARG00000011373	-1.8	0.041	mknk2a	MAP kinase interacting serine/threonine kinase 2a
ENSDARG00000022660	-1.8	0.000	armc2	armadillo repeat containing 2
ENSDARG00000023712	-1.8	0.007	mao	monoamine oxidase
ENSDARG00000054058	-1.8	0.007	h1fx	H1 histone family, member X
ENSDARG00000055510	-1.8	0.002	ypel3	yippee-like 3
ENSDARG00000002405	-1.7	0.004	si:ch211-225b11.1	si:ch211-225b11.1
ENSDARG00000009779	-1.7	0.001	mcl1a	myeloid cell leukemia 1a
ENSDARG00000074378	-1.7	0.010	junba	jun B proto-oncogene a
ENSDARG00000013794	-1.7	0.010	klf11b	Kruppel-like factor 11b
ENSDARG00000054848	-1.7	0.003	pdk4	pyruvate dehydrogenase kinase, isozyme 4
ENSDARG00000002758	-1.7	0.000	dedd1	death effector domain-containing 1
ENSDARG00000045522	-1.7	0.022	si:dkeyp-89c11.3	si:dkeyp-89c11.3
ENSDARG00000071626	-1.7	0.000	ptgdsb.2	prostaglandin D2 synthase b, tandem duplicate 2

ENSDARG00000036848	-1.7	0.049	slc43a2a	solute carrier family 43 (amino acid system L transporter), member 2a
ENSDARG00000062487	-1.7	0.021	si:dkey-6n6.1	si:dkey-6n6.1
ENSDARG00000045887	-1.7	0.021	mmp30	matrix metallopeptidase 30
ENSDARG00000036140	-1.7	0.000	crybgx	crystallin beta gamma X
ENSDARG00000006526	-1.7	0.019	fn1b	fibronectin 1b
ENSDARG00000045842	-1.7	0.049	zgc:113263	zgc:113263
ENSDARG00000094018	-1.7	0.035	si:dkey-111e8.1	si:dkey-111e8.1
ENSDARG00000100939	-1.7	0.002	LOC103909724	T-lymphocyte surface antigen Ly-9-like
ENSDARG00000029795	-1.7	0.013	fam213b	family with sequence similarity 213, member B
ENSDARG00000018146	-1.7	0.012	gpx1a	glutathione peroxidase 1a
ENSDARG00000104773	-1.7	0.021	junbb	jun B proto-oncogene b
ENSDARG00000074221	-1.6	0.049	zgc:172302	zgc:172302
ENSDARG00000025436	-1.6	0.046	msrb1a	methionine sulfoxide reductase B1a
ENSDARG00000025254	-1.6	0.008	s100a10b	S100 calcium binding protein A10b
ENSDARG00000020693	-1.6	0.010	sesn1	sestrin 1
ENSDARG00000055192	-1.6	0.006	zgc:136930	zgc:136930
ENSDARG00000036830	-1.6	0.002	krt17	keratin 91
ENSDARG00000094965	-1.6	0.009	nfil3-5	nuclear factor, interleukin 3 regulated, member 5
ENSDARG00000099749	-1.6	0.015	ube2d2l	ubiquitin-conjugating enzyme E2D 2 (UBC4/5 homolog, yeast)
ENSDARG00000045408	-1.6	0.043	tagln	transgelin
ENSDARG00000034883	-1.6	0.022	acbd5a	acyl-CoA binding domain containing 5a
ENSDARG00000102379	-1.6	0.032	si:zfos-80g12.1	si:zfos-80g12.1
ENSDARG00000031317	-1.6	0.007	ppdpfb	pancreatic progenitor cell differentiation and proliferation factor b
ENSDARG00000103639	-1.6	0.028	si:dkey-36i7.3	si:dkey-36i7.3
ENSDARG00000060113	-1.6	0.044	znf395a	zinc finger protein 395a
ENSDARG00000055101	-1.6	0.033	hmox2a	heme oxygenase 2a
ENSDARG00000040971	-1.6	0.022	zgc:92606	zgc:92606
ENSDARG00000045929	-1.5	0.013	oaz2a	ornithine decarboxylase antizyme 2a
ENSDARG00000036967	-1.5	0.021	smox	spermine oxidase
ENSDARG00000003216	-1.5	0.036	anxa2a	annexin A2a
ENSDARG00000091061	-1.5	0.045	slc38a3b	solute carrier family 38, member 3b
ENSDARG00000041665	-1.5	0.032	mkrn1	makorin, ring finger protein, 1
ENSDARG00000068996	-1.5	0.007	hist2h2l	histone 2, H2, like
ENSDARG00000104613	-1.5	0.036	znf1179	zinc finger protein 1179
ENSDARG00000063626	-1.5	0.016	ddx21	DEAD (Asp-Glu-Ala-Asp) box helicase 21
ENSDARG00000075048	-1.5	0.008	lonrf1	LON peptidase N-terminal domain and ring finger 1

Control: Incubation 32 ℃ × Challenge 24 ℃ vs Incubation 24 ℃ × Challenge 24 ℃

* Adjusted *p*-value (padj) < 0.05, Benjamin-Hochberg method
* Enrichment value = GeneRatio/BgRatio

Up-regulated

GO ID	Description	Gene Ratio	BgRatio	Padj	Gene Name	Count
GO:0042752	regulation of circadian rhythm	3/53	11/14763	0.001	per1b/si:ch211-132b12.7/cipcb	3
GO:0009266	response to temperature stimulus	3/53	28/14763	0.006	per1b/cirbpb/cry2	3
GO:0009314	response to radiation	5/53	139/14763	0.006	per1b/grk7a/per1a/cdkn1a/cry2	5
GO:0033059	cellular pigmentation	3/53	45/14763	0.016	pmelb/tyrp1b/pmela	3

Down-regulated

GO ID	Description	Gene Ratio	BgRatio	Padj	Gene Name	Count
GO:0006979	response to oxidative stress	5/97	55/14763	0.016	gpx1a/sesn1/msrb1a/msra/si:zfos-80g12.1	5

Supplementary Table S7. Comparison of DEGs and GO processes in control larvae between Incubation 24 °C × Challenge 32 °C and Incubation 24 °C × Challenge 24 °C.

Control: Incubation 24 ℃ × Challenge 32 ℃ vs Incubation 24 ℃ × Challenge 24 ℃ (1109 DEGs)

* Adjusted *p*-value (padj) < 0.05, Benjamin-Hochberg method; |fold change| ≥ 1.5

Up-regulated (494 DEGs)

Ensembl_ID	Fold_ Change	Padj	ZFIN_Symbol	Description
ENSDARG00000019949	20.2	0.000	serpinh1b	serpin peptidase inhibitor, clade H (heat shock protein 47), member 1b
ENSDARG00000037403	6.8	0.000	si:dkey-4p15.3	si:dkey-4p15.3
ENSDARG00000097268	5.6	0.000	si:ch73-171a6.2	si:ch73-171a6.2
ENSDARG00000010478	5.3	0.000	hsp90aa1.1	heat shock protein 90, alpha (cytosolic), class A member 1, tandem duplicate 1
ENSDARG00000024746	5.2	0.000	hsp90aa1.2	heat shock protein 90, alpha (cytosolic), class A member 1, tandem duplicate 2
ENSDARG00000069831	4.7	0.000	crygm2c	crystallin, gamma M2c
ENSDARG00000078876	4.2	0.000	crygm2b	crystallin, gamma M2b
ENSDARG00000059351	3.8	0.000	hnrnpa3	heterogeneous nuclear ribonucleoprotein A3
ENSDARG00000053502	3.8	0.000	cryaa	crystallin, alpha A
ENSDARG00000057911	3.5	0.000	zgc:86709	zgc:86709
ENSDARG00000075881	3.4	0.000	si:ch211-39k3.2	si:ch211-39k3.2
ENSDARG00000040278	3.4	0.000	klhl38b	kelch-like family member 38b
ENSDARG00000079307	3.4	0.000	si:dkey-205h13.1	si:dkey-205h13.1
ENSDARG00000075463	3.3	0.000	mss51	MSS51 mitochondrial translational activator
ENSDARG00000051762	3.3	0.000	dnaja4	DnaJ heat shock protein family (Hsp40) member A4
ENSDARG00000075855	3.2	0.000	adgre10	adhesion G protein-coupled receptor E10
ENSDARG00000030440	3.2	0.000	rsrp1	arginine/serine-rich protein 1
ENSDARG00000036371	3.0	0.000	acta1a	actin, alpha 1a, skeletal muscle
ENSDARG00000091253	2.9	0.000	smyd1b	SET and MYND domain containing 1b
ENSDARG00000051914	2.9	0.000	slc14a2	solute carrier family 14 (urea transporter), member 2
ENSDARG00000100795	2.9	0.000	timp4a	TIMP metallopeptidase inhibitor 4a
ENSDARG00000041394	2.8	0.000	dnajb1b	DnaJ (Hsp40) homolog, subfamily B, member 1b
ENSDARG00000075954	2.8	0.000	serpinh1a	serpin peptidase inhibitor, clade H (heat shock protein 47), member 1a
ENSDARG00000055595	2.8	0.000	clul1	clusterin-like 1 (retinal)
ENSDARG00000008447	2.8	0.000	fkbp4	FK506 binding protein 4
ENSDARG00000016477	2.7	0.000	eif4a2	eukaryotic translation initiation factor 4A, isoform 2
ENSDARG00000089205	2.7	0.000	si:dkey-73n8.3	si:dkey-73n8.3
ENSDARG00000035458	2.7	0.000	atp2a1l	ATPase, Ca++ transporting, cardiac muscle, fast twitch 1 like
ENSDARG00000036840	2.7	0.000	krt15	keratin 15
ENSDARG00000028625	2.6	0.000	jph2	junctophilin 2
ENSDARG00000062446	2.5	0.001	neurl2	neuralized E3 ubiquitin protein ligase 2
ENSDARG00000102490	2.5	0.000	asb2b	ankyrin repeat and SOCS box containing 2b
ENSDARG00000094000	2.5	0.000	si:dkey-153k10.6	si:dkey-153k10.6
ENSDARG00000030972	2.5	0.000	dnaja1	DnaJ (Hsp40) homolog, subfamily A, member 1
ENSDARG00000036894	2.5	0.000	zgc:101853	zgc:101853

ENSDARG00000007080	2.5	0.000	rhcgl1	Rh family, C glycoprotein, like 1
ENSDARG00000012381	2.5	0.000	hsc70	heat shock cognate 70
ENSDARG00000015422	2.5	0.000	ppil1	peptidylprolyl isomerase (cyclophilin)-like 1
ENSDARG00000099889	2.4	0.000	dusp27	dual specificity phosphatase 27 (putative)
ENSDARG00000041492	2.4	0.000	si:ch211-196f5.2	si:ch211-196f5.2
ENSDARG00000040277	2.4	0.004	fbxo32	F-box protein 32
ENSDARG00000038583	2.4	0.000	abraa	actin binding Rho activating protein a
ENSDARG00000033160	2.4	0.000	nr1d1	nuclear receptor subfamily 1, group d, member 1
ENSDARG00000010873	2.4	0.000	si:dkey-156n14.5	probable ATP-dependent RNA helicase DDX17
ENSDARG00000037191	2.4	0.000	ttr	transthyretin (prealbumin, amyloidosis type I)
ENSDARG00000030176	2.4	0.000	zgc:92429	zgc:92429
ENSDARG00000010085	2.3	0.000	p4ha2	procollagen-proline, 2-oxoglutarate 4-dioxygenase (proline 4-hydroxylase), alpha polypeptide 2
ENSDARG00000019874	2.3	0.000	hsph1	heat shock 105/110 protein 1
ENSDARG00000028027	2.3	0.000	trim63a	tripartite motif containing 63a
ENSDARG00000042552	2.3	0.000	cacna1sb	calcium channel, voltage-dependent, L type, alpha 1S subunit, b
ENSDARG00000101407	2.3	0.005	tgm1l4	transglutaminase 1 like 4
ENSDARG00000100443	2.3	0.000	si:ch211-71k24.8	si:ch211-71k24.8
ENSDARG00000010519	2.3	0.000	per3	period circadian clock 3
ENSDARG00000099805	2.3	0.000	LOC100332847	zinc finger protein 154-like
ENSDARG00000036832	2.3	0.000	cyt1l	type I cytokeratin, enveloping layer, like
ENSDARG00000020811	2.2	0.000	efemp2b	EGF containing fibulin-like extracellular matrix protein 2b
ENSDARG00000036830	2.2	0.000	krt17	keratin 91
ENSDARG00000095553	2.2	0.003	lenep	lens epithelial protein
ENSDARG00000010434	2.2	0.000	clu	clusterin
ENSDARG00000039299	2.2	0.000	rdh14a	retinol dehydrogenase 14a (all-trans/9-cis/11-cis)
ENSDARG00000076163	2.2	0.000	col19a1	collagen, type XIX, alpha 1
ENSDARG00000089920	2.2	0.000	mlip	muscular LMNA-interacting protein
ENSDARG00000079462	2.2	0.011	si:ch211-131k2.3	si:ch211-131k2.3
ENSDARG00000087301	2.2	0.000	crygm2d14	crystallin, gamma M2d14
ENSDARG00000007382	2.2	0.000	ubtd1a	ubiquitin domain containing 1a
ENSDARG00000104487	2.2	0.003	mthfs	5,10-methenyltetrahydrofolate synthetase (5-formyltetrahydrofolate cyclo-ligase)
ENSDARG00000062359	2.2	0.000	scn3b	sodium channel, voltage-gated, type III, beta
ENSDARG00000037894	2.2	0.000	ezh1	enhancer of zeste 1 polycomb repressive complex 2 subunit
ENSDARG00000069074	2.2	0.000	cry1ba	cryptochrome circadian clock 1ba
ENSDARG00000058630	2.2	0.000	zgc:162472	zgc:162472
ENSDARG00000057772	2.2	0.000	march7	membrane-associated ring finger (C3HC4) 7
ENSDARG00000008433	2.2	0.000	unc45b	unc-45 myosin chaperone B
ENSDARG00000101984	2.2	0.000	pde6g	phosphodiesterase 6G, cGMP-specific, rod, gamma
ENSDARG00000070597	2.2	0.001	prelp	proline/arginine-rich end leucine-rich repeat protein
ENSDARG00000090722	2.1	0.000	zgc:172246	zgc:172246

ENSDARG00000046014	2.1	0.003	kcna6a	potassium voltage-gated channel, shaker-related, subfamily, member 6 a
ENSDARG00000038858	2.1	0.000	setd7	SET domain containing (lysine methyltransferase) 7
ENSDARG00000079111	2.1	0.000	zgc:86725	zgc:86725
ENSDARG00000099817	2.1	0.000	tusc2a	tumor suppressor candidate 2a
ENSDARG00000105162	2.1	0.009	si:dkey-224k5.13	si:dkey-224k5.13
ENSDARG00000026726	2.1	0.000	anxa1a	annexin A1a
ENSDARG00000104204	2.1	0.000	slc1a8a	solute carrier family 1 (glutamate transporter), member 8a
ENSDARG00000057652	2.1	0.000	dbpb	D site albumin promoter binding protein b
ENSDARG00000098201	2.1	0.004	mtmr3	myotubularin related protein 3
ENSDARG00000105443	2.1	0.003	si:ch211-63p21.8	si:ch211-63p21.8
ENSDARG00000056915	2.1	0.000	si:ch211-237l4.6	si:ch211-237l4.6
ENSDARG00000023797	2.1	0.000	ryr1b	ryanodine receptor 1b (skeletal)
ENSDARG00000105422	2.1	0.011	si:dkey-1k23.3	si:dkey-1k23.3
ENSDARG00000042857	2.1	0.001	si:ch211-246m6.4	si:ch211-246m6.4
ENSDARG00000041339	2.1	0.000	zgc:92380	zgc:92380
ENSDARG00000008880	2.1	0.000	usp28	ubiquitin specific peptidase 28
ENSDARG00000090866	2.1	0.004	kcnj1a.2	potassium inwardly-rectifying channel, subfamily J, member 1a, tandem duplicate 2
ENSDARG00000032005	2.1	0.008	ccdc65	coiled-coil domain containing 65
ENSDARG00000079569	2.1	0.005	amigo3	amphoterin-induced protein 3-like
ENSDARG00000093647	2.1	0.007	si:dkey-48g21.7	si:dkey-48g21.7
ENSDARG00000056885	2.1	0.001	per1a	period circadian clock 1a
ENSDARG00000029830	2.1	0.010	myf6	myogenic factor 6
ENSDARG00000016570	2.1	0.000	prlra	prolactin receptor a
ENSDARG00000079055	2.1	0.003	si:dkey-85a20.4	si:dkey-85a20.4
ENSDARG00000063026	2.0	0.000	mlc1	megalencephalic leukoencephalopathy with subcortical cysts 1
ENSDARG00000038742	2.0	0.000	si:ch211-119o8.7	si:ch211-119o8.7
ENSDARG00000013997	2.0	0.000	ern1	endoplasmic reticulum to nucleus signaling 1
ENSDARG00000039502	2.0	0.000	eef1a1a	eukaryotic translation elongation factor 1 alpha 1a
ENSDARG00000045598	2.0	0.000	si:dkey-180p18.9	si:dkey-180p18.9
ENSDARG00000076241	2.0	0.000	txlnbb	taxilin beta b
ENSDARG00000079745	2.0	0.000	si:ch211-166a6.5	si:ch211-166a6.5
ENSDARG00000020239	2.0	0.000	lpin1	lipin 1
ENSDARG00000042055	2.0	0.003	fam129aa	family with sequence similarity 129, member Aa
ENSDARG00000055752	2.0	0.003	npas4a	neuronal PAS domain protein 4a
ENSDARG00000089124	2.0	0.009	si:ch211-5k11.2	hemoglobin embryonic subunit alpha
ENSDARG00000074216	2.0	0.002	wu:fc38h03	wu:fc38h03
ENSDARG00000097487	2.0	0.000	si:ch211-175f11.5	si:ch211-175f11.5
ENSDARG00000104478	2.0	0.000	cap2	CAP, adenylate cyclase-associated protein, 2 (yeast)
ENSDARG00000098051	2.0	0.001	opn6b	opsin 6, group member b
ENSDARG00000029822	2.0	0.016	cell	carboxyl ester lipase, tandem duplicate 2
ENSDARG00000100584	2.0	0.003	ccdc40	coiled-coil domain containing 40
ENSDARG00000096037	2.0	0.011	si:ch211-214j24.14	si:ch211-214j24.14

ENSDARG00000105325	2.0	0.019	si:cabz01044764.2	si:cabz01044764.2
ENSDARG00000069529	2.0	0.000	zgc:153981	zgc:153981
ENSDARG00000105183	2.0	0.024	si:ch211-198i6.4	si:ch211-198i6.4
ENSDARG00000058371	2.0	0.000	krt5	keratin 5
ENSDARG00000037554	2.0	0.027	arhgef3l	Rho guanine nucleotide exchange factor (GEF) 3, like
ENSDARG00000103586	2.0	0.003	si:dkey-65j6.2	si:dkey-65j6.2
ENSDARG00000097224	2.0	0.000	prr33	proline rich 33
ENSDARG00000019950	2.0	0.000	carf	calcium responsive transcription factor
ENSDARG00000016391	2.0	0.000	calcoco1b	transcription elongation factor B (SIII), polypeptide 3
ENSDARG00000076996	2.0	0.024	si:ch73-182a11.2	si:ch73-182a11.2
ENSDARG00000080337	2.0	0.000	AC024175.4	AC024175.4
ENSDARG00000098175	1.9	0.030	dgkab	diacylglycerol kinase, alpha b
ENSDARG00000022817	1.9	0.001	pvalb3	parvalbumin 3
ENSDARG00000099383	1.9	0.012	dnajb1a	DnaJ (Hsp40) homolog, subfamily B, member 1a
ENSDARG00000021163	1.9	0.000	thrb	thyroid hormone receptor beta
ENSDARG00000086917	1.9	0.000	crygm2d2	crystallin, gamma M2d2
ENSDARG00000101244	1.9	0.003	gli1	GLI family zinc finger 1
ENSDARG00000093318	1.9	0.000	si:dkey-57a22.15	si:dkey-57a22.15
ENSDARG00000079031	1.9	0.017	si:ch211-22d5.2	si:ch211-22d5.2
ENSDARG00000078159	1.9	0.008	zgc:175284	zgc:175284
ENSDARG00000037199	1.9	0.023	zgc:101851	zgc:101851
ENSDARG00000094890	1.9	0.005	si:ch211-163b2.4	si:ch211-163b2.4
ENSDARG00000014587	1.9	0.000	slc38a5b	solute carrier family 38, member 5b
ENSDARG00000045904	1.9	0.002	nr2e3	nuclear receptor subfamily 2, group E, member 3
ENSDARG00000058978	1.9	0.009	zgc:113423	zgc:113423
ENSDARG00000078535	1.9	0.001	lrrcc1	leucine rich repeat and coiled-coil centrosomal protein 1
ENSDARG00000088881	1.9	0.001	si:dkeyp-69b9.3	si:dkeyp-69b9.3
ENSDARG00000069792	1.9	0.000	crygm2d5	crystallin, gamma M2d5
ENSDARG00000003216	1.9	0.000	anxa2a	annexin A2a
ENSDARG00000000212	1.9	0.000	krt97	keratin 97
ENSDARG00000009018	1.9	0.000	rhbg	Rh family, B glycoprotein (gene/pseudogene)
ENSDARG00000033655	1.9	0.000	stmn1b	stathmin 1b
ENSDARG00000055223	1.9	0.018	ablim2	actin binding LIM protein family, member 2
ENSDARG00000051970	1.9	0.012	smu1b	smu-1 suppressor of mec-8 and unc-52 homolog b (C. elegans)
ENSDARG00000086100	1.9	0.039	cd302	CD302 molecule
ENSDARG00000101952	1.9	0.039	pigv	phosphatidylinositol glycan anchor biosynthesis, class V
ENSDARG00000041022	1.9	0.000	pdcd4b	programmed cell death 4b
ENSDARG00000097497	1.9	0.024	si:dkey-45m5.6	si:dkey-45m5.6
ENSDARG00000096216	1.9	0.037	si:ch211-162i8.7	si:ch211-162i8.7
ENSDARG00000069951	1.9	0.000	eef1a1b	eukaryotic translation elongation factor 1 alpha 1b
ENSDARG00000095675	1.9	0.003	ccdc141	coiled-coil domain containing 141

ENSDARG00000021564	1.9	0.020	zgc:56235	zgc:56235
ENSDARG00000052688	1.9	0.008	paqr5b	progestin and adipoQ receptor family member Vb
ENSDARG00000002916	1.9	0.021	sec31b	SEC31 homolog B, COPII coat complex component
ENSDARG00000006923	1.9	0.040	cacna1ab	calcium channel, voltage-dependent, P/Q type, alpha 1A subunit, b
ENSDARG00000037099	1.9	0.006	irs2a	insulin receptor substrate 2a
ENSDARG00000103000	1.9	0.046	si:ch211-107p11.3	si:ch211-107p11.3
ENSDARG00000094041	1.9	0.017	krt17	keratin 17
ENSDARG00000105441	1.9	0.000	pcdh11	protocadherin 11
ENSDARG00000077643	1.9	0.020	lypd6b	LY6/PLAUR domain containing 6B
ENSDARG00000022260	1.9	0.049	si:ch211-57i17.1	si:ch211-57i17.1
ENSDARG00000044875	1.8	0.035	crygm2e	crystallin, gamma M2e
ENSDARG00000014233	1.8	0.010	sept8b	septin 8b
ENSDARG00000016363	1.8	0.007	her8a	hairy-related 8a
ENSDARG00000043102	1.8	0.000	lxn	latexin
ENSDARG00000105587	1.8	0.000	si:dkey-111f13.3	si:dkey-111f13.3
ENSDARG00000102474	1.8	0.001	dusp16	dual specificity phosphatase 16
ENSDARG00000038731	1.8	0.000	slc25a36a	solute carrier family 25 (pyrimidine nucleotide carrier), member 36a
ENSDARG00000099511	1.8	0.011	CABZ01034698.4	CABZ01034698.4
ENSDARG00000051852	1.8	0.000	kcnc1a	potassium voltage-gated channel, Shaw-related subfamily, member 1a
ENSDARG00000079645	1.8	0.003	sc:d217	sc:d217
ENSDARG00000093244	1.8	0.000	si:ch211-32p8.1	si:ch211-32p8.1
ENSDARG00000075454	1.8	0.000	hdc	histidine decarboxylase
ENSDARG00000042848	1.8	0.004	pcmtl	l-isoaspartyl protein carboxyl methyltransferase, like
ENSDARG00000051981	1.8	0.041	zgc:165520	zgc:165520
ENSDARG00000007025	1.8	0.050	ttc9c	tetratricopeptide repeat domain 9C
ENSDARG00000075887	1.8	0.018	herc3	HECT and RLD domain containing E3 ubiquitin protein ligase 3
ENSDARG00000078095	1.8	0.000	cipcb	CLOCK-interacting pacemaker b
ENSDARG00000092895	1.8	0.017	si:dkey-188i13.11	si:dkey-188i13.11
ENSDARG00000014496	1.8	0.002	trpv6	transient receptor potential cation channel, subfamily V, member 6
ENSDARG00000098151	1.8	0.031	si:ch211-249o11.3	si:ch211-249o11.3
ENSDARG00000015164	1.8	0.000	mknk2b	MAP kinase interacting serine/threonine kinase 2b
ENSDARG00000056888	1.8	0.046	si:dkeyp-87a6.1	si:dkeyp-87a6.1
ENSDARG00000105001	1.8	0.000	zgc:91999	zgc:91999
ENSDARG00000053803	1.8	0.002	klhl43	kelch-like family member 43
ENSDARG00000033854	1.8	0.001	abrab	actin binding Rho activating protein b
ENSDARG00000063635	1.8	0.007	nrxn1b	neurexin 1b
ENSDARG00000024195	1.8	0.000	znf395b	zinc finger protein 395b
ENSDARG00000099970	1.8	0.000	malat1	CR383676.1
ENSDARG00000075993	1.8	0.001	clic5a	chloride intracellular channel 5a
ENSDARG00000097208	1.8	0.003	si:ch1073-281m9.1	si:ch1073-281m9.1

ENSDARG00000002894	1.8	0.000	aven	apoptosis, caspase activation inhibitor
ENSDARG00000088023	1.8	0.007	si:dkey-26c10.5	si:dkey-26c10.5
ENSDARG00000088798	1.8	0.048	plcxd1	phosphatidylinositol-specific phospholipase C, X domain containing 1
ENSDARG00000071076	1.8	0.001	ldhbb	lactate dehydrogenase Bb
ENSDARG00000096398	1.8	0.034	si:ch211-276a17.5	si:ch211-276a17.5
ENSDARG00000011785	1.8	0.025	tbx6	T-box 6
ENSDARG00000104758	1.8	0.043	zgc:194981	zgc:194981
ENSDARG00000092033	1.8	0.001	si:dkey-239h2.3	si:dkey-239h2.3
ENSDARG00000071331	1.8	0.000	ryr3	ryanodine receptor 3
ENSDARG00000079347	1.8	0.003	zgc:194659	zgc:194659
ENSDARG00000056605	1.8	0.000	wbp2	WW domain binding protein 2
ENSDARG00000055192	1.8	0.000	zgc:136930	zgc:136930
ENSDARG00000038288	1.8	0.005	cd151	CD151 molecule
ENSDARG00000098765	1.8	0.041	si:ch211-167b20.9	si:ch211-167b20.9
ENSDARG00000061314	1.8	0.003	ngrn	neugrin, neurite outgrowth associated
ENSDARG00000017757	1.8	0.022	pik3cg	phosphatidylinositol-4,5-bisphosphate 3-kinase, catalytic subunit gamma
ENSDARG00000100288	1.8	0.048	impg2b	interphotoreceptor matrix proteoglycan 2b
ENSDARG00000088567	1.8	0.016	si:ch211-203d17.1	si:ch211-203d17.1
ENSDARG00000052019	1.8	0.019	si:ch73-109d9.1	si:ch73-109d9.1
ENSDARG00000104071	1.8	0.000	zgc:110091	zgc:110091
ENSDARG00000097528	1.8	0.000	si:dkey-7j14.5	si:dkey-7j14.5
ENSDARG00000100095	1.8	0.000	anxa1b	annexin A1b
ENSDARG00000102802	1.8	0.000	brdt	bromodomain, testis-specific
ENSDARG00000021265	1.8	0.001	mybpc2b	myosin binding protein C, fast type b
ENSDARG00000062363	1.8	0.001	phex	phosphate regulating endopeptidase homolog, X-linked
ENSDARG00000031890	1.8	0.005	tcp11l1	t-complex 11, testis-specific-like 1
ENSDARG00000076779	1.8	0.000	fam13b	family with sequence similarity 13, member B
ENSDARG00000062277	1.8	0.009	ppp2r2cb	protein phosphatase 2, regulatory subunit B, gamma b
ENSDARG00000018149	1.8	0.000	slc38a4	solute carrier family 38, member 4
ENSDARG00000057368	1.8	0.009	rps8b	ribosomal protein S8b
ENSDARG00000069030	1.8	0.017	skor1a	SKI family transcriptional corepressor 1a
ENSDARG00000067701	1.8	0.004	myoz3a	myozenin 3a
ENSDARG00000003203	1.7	0.014	rhcga	Rh family, C glycoprotein a
ENSDARG00000035870	1.7	0.000	laptm4b	lysosomal protein transmembrane 4 beta
ENSDARG00000095041	1.7	0.024	si:rp71-78h1.10	si:rp71-78h1.10
ENSDARG00000021250	1.7	0.015	slc25a48	solute carrier family 25, member 48
ENSDARG00000055510	1.7	0.000	ypel3	yippee-like 3
ENSDARG00000056726	1.7	0.022	mettl7a	methyltransferase like 7A
ENSDARG00000098994	1.7	0.000	CR352263.1	CR352263.1
ENSDARG00000099961	1.7	0.000	bnip3	BCL2/adenovirus E1B interacting protein 3
ENSDARG00000009142	1.7	0.000	ppp1r13bb	protein phosphatase 1, regulatory subunit 13Bb
ENSDARG00000087765	1.7	0.000	crygm2d10	crystallin, gamma M2d10

ENSDARG00000026359	1.7	0.035	pbld2	phenazine biosynthesis-like protein domain containing 2
ENSDARG00000101597	1.7	0.016	si:dkeyp-57d7.4	si:dkeyp-57d7.4
ENSDARG00000100963	1.7	0.005	si:ch211-60d22.1	si:ch211-60d22.1
ENSDARG00000093745	1.7	0.043	htr1aa	5-hydroxytryptamine (serotonin) receptor 1A a
ENSDARG00000056209	1.7	0.000	myoz1a	myozenin 1a
ENSDARG00000007601	1.7	0.010	zmynd8	zinc finger, MYND-type containing 8
ENSDARG00000041179	1.7	0.002	crygm5	crystallin, gamma M5
ENSDARG00000014731	1.7	0.001	cacybp	calcyclin binding protein
ENSDARG00000102493	1.7	0.045	ticam1	toll-like receptor adaptor molecule 1
ENSDARG00000078546	1.7	0.009	cars2	cysteinyl-tRNA synthetase 2, mitochondrial
ENSDARG00000092283	1.7	0.001	cxl34b.11	CX chemokine ligand 34b, duplicate 11
ENSDARG00000076843	1.7	0.017	si:dkey-174i8.1	si:dkey-174i8.1
ENSDARG00000076533	1.7	0.006	nfe2l1b	nuclear factor, erythroid 2-like 1b
ENSDARG00000100227	1.7	0.003	zgc:63694	zgc:63694
ENSDARG00000035327	1.7	0.000	ckma	creatine kinase, muscle a
ENSDARG00000102379	1.7	0.000	si:zfos-80g12.1	si:zfos-80g12.1
ENSDARG00000099362	1.7	0.013	LOC101886884	anoctamin-1-like
ENSDARG00000105079	1.7	0.047	si:ch211-157e21.2	si:ch211-157e21.2
ENSDARG00000098392	1.7	0.000	si:ch73-28h20.1	si:ch73-28h20.1
ENSDARG00000016745	1.7	0.000	slc35f6	solute carrier family 35, member F6
ENSDARG00000052515	1.7	0.011	calcoco2	calcium binding and coiled-coil domain 2
ENSDARG00000060578	1.7	0.008	si:dkey-226m8.10	uncharacterized LOC100001344
ENSDARG00000055618	1.7	0.000	acta1b	actin, alpha 1b, skeletal muscle
ENSDARG00000059028	1.7	0.002	srrd	SRR1 domain containing
ENSDARG00000100233	1.7	0.002	si:ch1073-100e22.1	si:ch1073-100e22.1
ENSDARG00000044935	1.7	0.000	hpdb	4-hydroxyphenylpyruvate dioxygenase b
ENSDARG00000008904	1.7	0.000	smarca2	SWI/SNF related, matrix associated, actin dependent regulator of chromatin, subfamily a, member 2
ENSDARG00000089372	1.7	0.000	clk4a	CDC-like kinase 4a
ENSDARG00000071673	1.7	0.000	ctdspla	CTD (carboxy-terminal domain, RNA polymerase II, polypeptide A) small phosphatase-like a
ENSDARG00000036140	1.7	0.000	crybgx	crystallin beta gamma X
ENSDARG00000089225	1.7	0.004	trove2	TROVE domain family, member 2
ENSDARG00000103125	1.7	0.000	tsc2	tuberous sclerosis 2
ENSDARG00000101367	1.7	0.016	si:ch73-193i22.1	si:ch73-193i22.1
ENSDARG00000022509	1.7	0.000	cox4i2	cytochrome c oxidase subunit IV isoform 2
ENSDARG00000002758	1.7	0.000	dedd1	death effector domain-containing 1
ENSDARG00000078911	1.7	0.000	zgc:163098	zgc:163098
ENSDARG00000057299	1.7	0.000	pbdc1	polysaccharide biosynthesis domain containing 1
ENSDARG00000076903	1.7	0.049	bend3	BEN domain containing 3
ENSDARG00000023181	1.7	0.011	pcp4l1	Purkinje cell protein 4 like 1
ENSDARG00000045071	1.7	0.002	chad	chondroadherin
ENSDARG00000078814	1.7	0.028	si:dkey-34m19.3	si:dkey-34m19.3
ENSDARG00000063354	1.7	0.011	abtb1	ankyrin repeat and BTB (POZ) domain containing 1

ENSDARG00000058587	1.7	0.038	ccdc79	coiled-coil domain containing 79
ENSDARG00000069269	1.7	0.014	wdr35	WD repeat domain 35
ENSDARG00000035810	1.7	0.001	rgcc	regulator of cell cycle
ENSDARG00000104685	1.7	0.002	grk1b	G protein-coupled receptor kinase 1 b
ENSDARG00000012044	1.7	0.030	polr3gla	polymerase (RNA) III (DNA directed) polypeptide G like a
ENSDARG00000001999	1.7	0.046	adamts18	ADAM metallopeptidase with thrombospondin type 1 motif, 18
ENSDARG00000008788	1.7	0.001	camk1gb	calcium/calmodulin-dependent protein kinase IGb
ENSDARG00000070818	1.7	0.004	pax7b	paired box 7b
ENSDARG00000036895	1.7	0.004	dap1b	death associated protein 1b
ENSDARG00000076229	1.7	0.000	mkl1b	megakaryoblastic leukemia (translocation) 1b
ENSDARG00000076074	1.7	0.000	dda1	DET1 and DDB1 associated 1
ENSDARG00000094362	1.7	0.000	si:dkey-162b3.5	si:dkey-162b3.5
ENSDARG00000019063	1.7	0.030	fat1b	FAT atypical cadherin 1b
ENSDARG00000031343	1.7	0.000	rab6bb	RAB6B, member RAS oncogene family b
ENSDARG00000089458	1.6	0.013	rp1l1a	retinitis pigmentosa 1-like 1a
ENSDARG00000077256	1.6	0.000	nat8l	N-acetyltransferase 8-like
ENSDARG00000076170	1.6	0.000	pcsk1nl	proprotein convertase subtilisin/kexin type 1 inhibitor, like
ENSDARG00000086842	1.6	0.000	dap1b	death-associated protein-like 1-B
ENSDARG00000097445	1.6	0.033	si:dkey-195m11.8	si:dkey-195m11.8
ENSDARG00000033138	1.6	0.002	lyrm2	LYR motif containing 2
ENSDARG00000067990	1.6	0.000	myhz1.1	myosin, heavy polypeptide 1.1, skeletal muscle
ENSDARG00000021372	1.6	0.000	tob1b	transducer of ERBB2, 1b
ENSDARG00000075282	1.6	0.001	irs2b	insulin receptor substrate 2b
ENSDARG00000039255	1.6	0.006	klhl21	kelch-like family member 21
ENSDARG00000059020	1.6	0.013	thap1	THAP domain containing, apoptosis associated protein 1
ENSDARG00000056477	1.6	0.021	ccdc125	coiled-coil domain containing 125
ENSDARG00000086658	1.6	0.000	crygm2d21	crystallin, gamma M2d21
ENSDARG00000015404	1.6	0.020	arl3l2	ADP-ribosylation factor-like 3, like 2
ENSDARG00000097962	1.6	0.030	si:ch211-214g1.4	si:ch211-214g1.4
ENSDARG00000055722	1.6	0.029	bco2a	beta-carotene oxygenase 2a
ENSDARG00000055868	1.6	0.000	rsl1d1	ribosomal L1 domain containing 1
ENSDARG00000039008	1.6	0.020	zgc:85858	zgc:85858
ENSDARG00000039211	1.6	0.005	zgc:77439	zgc:77439
ENSDARG00000056805	1.6	0.018	plch2b	phospholipase C, eta 2b
ENSDARG00000025605	1.6	0.016	nvl	nuclear VCP-like
ENSDARG00000007421	1.6	0.014	ftcd	formimidoyltransferase cyclodeaminase
ENSDARG00000087164	1.6	0.000	crygm2d4	crystallin, gamma M2d4
ENSDARG00000062902	1.6	0.021	si:dkey-220f10.4	si:dkey-220f10.4
ENSDARG00000083312	1.6	0.034	AC024175.21	AC024175.21
ENSDARG00000038074	1.6	0.000	ergic3	ERGIC and golgi 3
ENSDARG00000086107	1.6	0.046	mterf1	mitochondrial transcription termination factor 1
ENSDARG00000046010	1.6	0.008	kdm2bb	lysine-specific demethylase 2B

ENSDARG00000012504	1.6	0.003	rlbp1a	retinaldehyde binding protein 1a
ENSDARG00000041665	1.6	0.000	mkrn1	makorin, ring finger protein, 1
ENSDARG00000043542	1.6	0.014	zpr1	ZPR1 zinc finger
ENSDARG00000087857	1.6	0.038	si:ch211-180a12.2	si:ch211-180a12.2
ENSDARG00000004580	1.6	0.004	nmnat2	nicotinamide nucleotide adenylyltransferase 2
ENSDARG00000062727	1.6	0.002	cep290	centrosomal protein 290
ENSDARG00000035332	1.6	0.024	c10h21orf59	c10h21orf59 homolog (H. sapiens)
ENSDARG00000007171	1.6	0.042	aig1	androgen-induced 1 (H. sapiens)
ENSDARG00000053110	1.6	0.005	pkib	protein kinase (cAMP-dependent, catalytic) inhibitor beta
ENSDARG00000035256	1.6	0.000	eef2l2	eukaryotic translation elongation factor 2, like 2
ENSDARG00000097055	1.6	0.025	si:ch211-284f22.3	si:ch211-284f22.3
ENSDARG00000105368	1.6	0.009	si:ch211-276i12.12	si:ch211-276i12.12
ENSDARG00000092557	1.6	0.046	si:ch211-69k21.3	si:ch211-69k21.3
ENSDARG00000028815	1.6	0.042	zgc:162945	zgc:162945
ENSDARG00000033382	1.6	0.013	grifin	galectin-related inter-fiber protein
ENSDARG00000075461	1.6	0.009	suv420h2	suppressor of variegation 4-20 homolog 2 (Drosophila)
ENSDARG00000056121	1.6	0.018	serac1	serine active site containing 1
ENSDARG00000031240	1.6	0.001	kidins220a	kinase D-interacting substrate 220a
ENSDARG00000076679	1.6	0.002	rai1	retinoic acid induced 1
ENSDARG00000102772	1.6	0.014	klhdc3	kelch domain containing 3
ENSDARG00000076693	1.6	0.000	crygm2d19	crystallin, gamma M2d19
ENSDARG00000052575	1.6	0.023	arl13a	ADP-ribosylation factor-like 13A
ENSDARG00000025436	1.6	0.003	msrb1a	methionine sulfoxide reductase B1a
ENSDARG00000045685	1.6	0.011	cntn1b	contactin 1b
ENSDARG00000103845	1.6	0.001	zgc:55733	zgc:55733
ENSDARG00000074779	1.6	0.020	alms1	Alstrom syndrome protein 1
ENSDARG00000042322	1.6	0.001	si:dkey-177p2.6	si:dkey-177p2.6
ENSDARG00000001882	1.6	0.006	kbtbd12	kelch repeat and BTB (POZ) domain containing 12
ENSDARG00000076440	1.6	0.007	btbd6a	BTB (POZ) domain containing 6a
ENSDARG00000058032	1.6	0.012	si:dkey-6n6.2	si:dkey-6n6.2
ENSDARG00000068637	1.6	0.015	si:ch211-281l24.3	si:ch211-281l24.3
ENSDARG00000041223	1.6	0.045	fam173a	family with sequence similarity 173, member A
ENSDARG00000070122	1.6	0.007	stk11ip	serine/threonine kinase 11 interacting protein
ENSDARG00000103908	1.6	0.006	LOC100535417	fumarate hydratase, mitochondrial-like
ENSDARG00000102506	1.6	0.018	lrp2a	low density lipoprotein receptor-related protein 2a
ENSDARG00000098855	1.6	0.044	osxm	3-oxoacyl-ACP synthase, mitochondrial
ENSDARG00000062855	1.6	0.043	plekhg7	pleckstrin homology domain containing, family G (with RhoGef domain) member 7
ENSDARG00000017602	1.6	0.000	ccng2	cyclin G2
ENSDARG00000091148	1.6	0.000	crygm2d5	crystallin, gamma M2d20
ENSDARG00000052000	1.6	0.028	cav2	caveolin 2
ENSDARG00000090914	1.6	0.001	si:ch211-117k10.3	si:ch211-117k10.3
ENSDARG00000100406	1.6	0.031	zgc:112265	zgc:112265

ENSDARG00000016835	1.6	0.018	tcirg1a	T-cell, immune regulator 1, ATPase, H+ transporting, lysosomal V0 subunit A3a
ENSDARG00000035978	1.6	0.008	ube3c	ubiquitin protein ligase E3C
ENSDARG00000095147	1.6	0.033	krt96	keratin 96
ENSDARG00000073747	1.6	0.006	si:dkey-1j5.4	si:dkey-1j5.4
ENSDARG00000027501	1.6	0.011	usp20	ubiquitin specific peptidase 20
ENSDARG00000003281	1.6	0.017	pik3ip1	phosphoinositide-3-kinase interacting protein 1
ENSDARG00000069048	1.6	0.002	serpinf1	serpin peptidase inhibitor, clade F (alpha-2 antiplasmin, pigment epithelium derived factor), member 1
ENSDARG00000059259	1.6	0.000	pabpc4	poly(A) binding protein, cytoplasmic 4 (inducible form)
ENSDARG00000035835	1.6	0.000	eef2k	eukaryotic elongation factor 2 kinase
ENSDARG00000027744	1.6	0.025	gadd45ba	growth arrest and DNA-damage-inducible, beta a
ENSDARG00000069100	1.6	0.000	aldh9a1a.1	aldehyde dehydrogenase 9 family, member A1a, tandem duplicate 1
ENSDARG00000040565	1.6	0.000	ckmb	creatine kinase, muscle b
ENSDARG00000099954	1.6	0.022	plekhm1	pleckstrin homology domain containing, family M (with RUN domain) member 1
ENSDARG00000009341	1.6	0.001	nrap	nebulin-related anchoring protein
ENSDARG00000038978	1.6	0.017	dnajb4	DnaJ (Hsp40) homolog, subfamily B, member 4
ENSDARG00000004537	1.6	0.000	kmt2a	lysine (K)-specific methyltransferase 2A
ENSDARG00000007715	1.6	0.001	lgsn	lengsin, lens protein with glutamine synthetase domain
ENSDARG00000070826	1.6	0.004	bpgm	2,3-bisphosphoglycerate mutase
ENSDARG00000078989	1.6	0.018	alpk3a	alpha-kinase 3a
ENSDARG00000030775	1.6	0.016	sybl1	synaptobrevin-like 1
ENSDARG00000034522	1.6	0.004	rab6ba	RAB6B, member RAS oncogene family a
ENSDARG00000037789	1.6	0.000	pvalb1	parvalbumin 1
ENSDARG00000006307	1.6	0.010	shisa4	shisa family member 4
ENSDARG00000011208	1.6	0.006	cox5b	cytochrome c oxidase subunit 5B
ENSDARG00000028721	1.6	0.005	mapk14b	mitogen-activated protein kinase 14b
ENSDARG00000008639	1.6	0.003	kifap3a	kinesin-associated protein 3a
ENSDARG00000102472	1.6	0.039	btbd17b	BTB (POZ) domain containing 17b
ENSDARG00000054087	1.6	0.016	irs1	insulin receptor substrate 1
ENSDARG00000069796	1.6	0.043	znf1124	zinc finger protein 1124
ENSDARG00000097706	1.6	0.015	birc6-as2	birc6 antisense RNA 2
ENSDARG00000059498	1.6	0.050	arl16	ADP-ribosylation factor-like 16
ENSDARG00000053113	1.6	0.014	ly75	lymphocyte antigen 75
ENSDARG00000043701	1.6	0.015	gpd1a	glycerol-3-phosphate dehydrogenase 1a
ENSDARG00000039066	1.6	0.000	klhl31	kelch-like family member 31
ENSDARG00000062361	1.6	0.001	zgc:158234	zgc:158234
ENSDARG00000053453	1.6	0.002	mpp2a	membrane protein, palmitoylated 2a (MAGUK p55 subfamily member 2)
ENSDARG00000030349	1.6	0.000	cryba2a	crystallin, beta A2a
ENSDARG00000078114	1.6	0.022	si:ch73-237c6.1	si:ch73-237c6.1
ENSDARG00000105529	1.6	0.001	vgf	VGF nerve growth factor inducible

ENSDARG00000042535	1.6	0.000	actc1a	actin, alpha, cardiac muscle 1a
ENSDARG00000100313	1.6	0.006	pip5k1cb	si:ch211-243a15.1
ENSDARG00000104172	1.6	0.045	diabloa	diablo, IAP-binding mitochondrial protein a
ENSDARG00000058656	1.6	0.000	desma	desmin a
ENSDARG00000034470	1.6	0.000	aldoab	aldolase a, fructose-bisphosphate, b
ENSDARG00000098820	1.6	0.019	ftr67	finTRIM family, member 67
ENSDARG00000068507	1.6	0.000	crybb1	crystallin, beta B1
ENSDARG00000053254	1.6	0.000	mylpfa	myosin light chain, phosphorylatable, fast skeletal muscle a
ENSDARG00000013880	1.6	0.049	spata20	spermatogenesis associated 20
ENSDARG00000099106	1.6	0.013	afmid	arylformamidase
ENSDARG00000003820	1.6	0.000	nr1d2a	nuclear receptor subfamily 1, group D, member 2a
ENSDARG00000094460	1.6	0.032	si:dkey-100j22.4	si:dkey-100j22.4
ENSDARG00000076572	1.6	0.000	crygm2d7	crystallin, gamma M2d7
ENSDARG00000056938	1.6	0.037	kera	keratocan
ENSDARG00000100431	1.5	0.006	spaca4l	sperm acrosome membrane-associated protein 4
ENSDARG00000029443	1.5	0.012	zgc:92242	zgc:92242
ENSDARG00000069801	1.5	0.000	crygm2d12	crystallin, gamma M2d12
ENSDARG00000038076	1.5	0.000	romo1	reactive oxygen species modulator 1
ENSDARG00000002745	1.5	0.001	tdh	L-threonine dehydrogenase
ENSDARG00000026664	1.5	0.003	uri1	URI1, prefoldin-like chaperone
ENSDARG00000078151	1.5	0.048	si:ch211-264f5.8	si:ch211-264f5.8
ENSDARG00000062487	1.5	0.002	si:dkey-6n6.1	si:dkey-6n6.1
ENSDARG00000030154	1.5	0.010	pak7	p21 protein (Cdc42/Rac)-activated kinase 7
ENSDARG00000105018	1.5	0.014	lrrc20	leucine rich repeat containing 20
ENSDARG00000044420	1.5	0.049	dnajc19	DnaJ (Hsp40) homolog, subfamily C, member 19
ENSDARG00000013755	1.5	0.000	actn3a	actinin alpha 3a
ENSDARG00000069827	1.5	0.000	crygm2d11	crystallin, gamma M2d11
ENSDARG00000095991	1.5	0.034	si:ch211-199c19.4	si:ch211-199c19.4
ENSDARG00000012776	1.5	0.037	smarca1	SWI/SNF related, matrix associated, actin dependent regulator of chromatin, subfamily a, member 1
ENSDARG00000073766	1.5	0.032	tnni1d	troponin I, skeletal, slow d
ENSDARG00000057918	1.5	0.004	tsc1b	tuberous sclerosis 1b
ENSDARG00000079931	1.5	0.036	snx19b	sorting nexin 19b
ENSDARG00000071518	1.5	0.024	krt222	keratin 222
ENSDARG00000062352	1.5	0.044	sema4ab	sema domain, immunoglobulin domain (Ig), transmembrane domain (TM) and short cytoplasmic domain, (semaphorin) 4Ab
ENSDARG00000051800	1.5	0.000	fnbp4	formin binding protein 4
ENSDARG00000008487	1.5	0.006	dmd	dystrophin
ENSDARG00000092677	1.5	0.014	coa6	cytochrome c oxidase assembly factor 6
ENSDARG00000020761	1.5	0.023	arrdc2	arrestin domain containing 2
ENSDARG00000005416	1.5	0.006	map3k5	mitogen-activated protein kinase kinase kinase 5
ENSDARG00000015807	1.5	0.025	rab22a	RAB22A, member RAS oncogene family
ENSDARG00000035400	1.5	0.000	btf3	basic transcription factor 3

ENSDARG00000041317	1.5	0.002	rangap1a	RAN GTPase activating protein 1a
ENSDARG00000071196	1.5	0.000	sdprb	serum deprivation response b
ENSDARG00000032272	1.5	0.035	sat1b	spermidine/spermine N1-acetyltransferase 1b
ENSDARG00000026484	1.5	0.049	rab15	RAB15, member RAS oncogene family
ENSDARG00000042133	1.5	0.012	si:dkey-51e6.1	si:dkey-51e6.1
ENSDARG00000070220	1.5	0.050	pou3f2a	POU class 3 homeobox 2a
ENSDARG00000063229	1.5	0.003	xpo1b	exportin 1 (CRM1 homolog, yeast) b
ENSDARG00000060330	1.5	0.036	si:ch211-284e13.4	si:ch211-284e13.4
ENSDARG00000086342	1.5	0.007	zgc:101566	zgc:101566
ENSDARG00000036414	1.5	0.009	si:ch73-335l21.1	si:ch73-335l21.1
ENSDARG00000076223	1.5	0.000	zgc:92744	zgc:92744
ENSDARG00000075132	1.5	0.000	fh	fumarate hydratase
ENSDARG00000040177	1.5	0.004	rgs16	regulator of G-protein signaling 16
ENSDARG00000027611	1.5	0.002	sdpra	serum deprivation response a
ENSDARG00000070511	1.5	0.002	si:dkey-183j2.10	si:dkey-183j2.10
ENSDARG00000054272	1.5	0.000	caprin1b	cell cycle associated protein 1b
ENSDARG00000040463	1.5	0.039	slc25a51b	solute carrier family 25, member 51b
ENSDARG00000094692	1.5	0.000	si:ch211-122a17.5	si:ch211-122a17.5
ENSDARG00000097205	1.5	0.001	ulk2	unc-51 like autophagy activating kinase 2
ENSDARG00000076667	1.5	0.000	ccng1	cyclin G1
ENSDARG00000100317	1.5	0.018	ahsa1b	AHA1, activator of heat shock protein ATPase homolog 1b
ENSDARG00000056932	1.5	0.000	tfip11	tuftelin interacting protein 11
ENSDARG00000091111	1.5	0.020	si:ch211-15b10.6	si:ch211-15b10.6
ENSDARG00000042477	1.5	0.009	nr2c2	nuclear receptor subfamily 2, group C, member 2
ENSDARG00000077237	1.5	0.015	rfx7	regulatory factor X, 7
ENSDARG00000105302	1.5	0.013	lrrn2	leucine rich repeat neuronal 2
ENSDARG00000036096	1.5	0.009	smad3a	SMAD family member 3a
ENSDARG00000045019	1.5	0.000	aamp	angio-associated, migratory cell protein
ENSDARG00000009280	1.5	0.000	smyd1a	SET and MYND domain containing 1a
ENSDARG00000058039	1.5	0.005	bhlhe22	basic helix-loop-helix family, member e22
ENSDARG00000002249	1.5	0.017	tbxas1	thromboxane A synthase 1 (platelet)
ENSDARG00000028276	1.5	0.025	si:ch211-196h16.12	si:ch211-196h16.12
ENSDARG00000102712	1.5	0.002	si:ch211-71k24.7	si:ch211-71k24.7
ENSDARG00000007639	1.5	0.017	cnot4b	CCR4-NOT transcription complex, subunit 4b
ENSDARG00000025788	1.5	0.028	chp2	calcineurin-like EF-hand protein 2
ENSDARG00000077608	1.5	0.015	gpr137bb	integral membrane protein GPR137B-like
ENSDARG00000019081	1.5	0.011	zgc:101723	zgc:101723
ENSDARG00000060029	1.5	0.036	fam160b2	family with sequence similarity 160, member B2
ENSDARG00000101959	1.5	0.039	etv1	ets variant 1
ENSDARG00000006990	1.5	0.017	creld1b	cysteine-rich with EGF-like domain protein 1
ENSDARG00000053840	1.5	0.017	ei24	etoposide induced 2.4
ENSDARG00000067795	1.5	0.043	crfb6	cytokine receptor family member b6
ENSDARG00000037783	1.5	0.001	proza	protein Z, vitamin K-dependent plasma glycoprotein a
ENSDARG00000010442	1.5	0.011	rnf11a	ring finger protein 11a

ENSDARG00000098376	1.5	0.019	si:ch211-207l14.1	si:ch211-207l14.1
ENSDARG00000024669	1.5	0.015	oser1	oxidative stress responsive serine-rich 1
ENSDARG00000101387	1.5	0.008	si:dkey-33m11.6	si:dkey-33m11.6
ENSDARG00000056647	1.5	0.046	klhl3	kelch-like family member 3

Down-regulated (615 DEGs)

Ensembl_ID	Fold_ Change	Padj	ZFIN_Symbol	Description
ENSDARG00000093888	-4.8	0.000	si:dkeyp-87e3.1	si:dkeyp-87e3.1
ENSDARG00000056725	-4.3	0.000	hmgb3a	high mobility group box 3a
ENSDARG00000054753	-4.2	0.000	col10a1a	collagen, type X, alpha 1a
ENSDARG00000055644	-4.1	0.000	prss60.2	protease, serine, 60.2
ENSDARG00000105341	-4.1	0.000	si:dkey-9l20.3	si:dkey-9l20.3
ENSDARG00000091996	-3.7	0.000	si:ch211-117m20.5	si:ch211-117m20.5
ENSDARG00000074642	-3.6	0.000	zgc:153932	uncharacterized LOC108179107
ENSDARG00000006220	-3.6	0.000	ugt1ab	UDP glucuronosyltransferase 1 family, polypeptide A2
ENSDARG00000071592	-3.5	0.000	aqp8a.2	aquaporin 8a, tandem duplicate 2
ENSDARG00000042816	-3.4	0.000	mmp9	matrix metallopeptidase 9
ENSDARG00000068220	-3.4	0.000	hbl3	hexose-binding lectin 3
ENSDARG00000053625	-3.3	0.000	anxa2b	annexin A2b
ENSDARG00000001993	-3.3	0.000	myhb	myosin, heavy chain b
ENSDARG00000098475	-3.3	0.000	arr3b	arrestin 3b, retinal (X-arrestin)
ENSDARG00000058799	-3.3	0.000	mybpha	myosin binding protein Ha
ENSDARG00000046098	-3.2	0.000	ebp	emopamil binding protein (sterol isomerase)
ENSDARG00000092704	-3.2	0.000	BX072532.1	BX072532.1
ENSDARG00000042780	-3.1	0.000	apoba	apolipoprotein Ba
ENSDARG00000103019	-3.0	0.000	gstp2	glutathione S-transferase pi 2
ENSDARG00000004141	-3.0	0.000	zgc:92630	zgc:92630
ENSDARG00000058206	-3.0	0.000	si:ch211-153b23.5	si:ch211-153b23.5
ENSDARG00000051925	-3.0	0.000	cyb5r2	cytochrome b5 reductase 2
ENSDARG00000016081	-2.9	0.000	cldn15la	claudin 15-like a
ENSDARG00000094570	-2.9	0.000	si:ch211-226h7.2	si:ch211-226h7.2
ENSDARG00000101393	-2.8	0.000	si:dkey-31g6.6	si:dkey-31g6.6
ENSDARG00000095863	-2.8	0.000	afp4	antifreeze protein type IV
ENSDARG00000037551	-2.8	0.000	pm20d1.1	peptidase M20 domain containing 1, tandem duplicate 1
ENSDARG00000035958	-2.7	0.000	tnni2b.1	troponin I type 2b (skeletal, fast), tandem duplicate 1
ENSDARG00000070021	-2.7	0.000	cyp3c4	cytochrome P450, family 3, subfamily C, polypeptide 4
ENSDARG00000073799	-2.7	0.001	zgc:194210	zgc:194210
ENSDARG00000094466	-2.7	0.000	si:ch73-199e17.1	si:ch73-199e17.1
ENSDARG00000070775	-2.6	0.000	cyp2x9	cytochrome P450, family 2, subfamily X, polypeptide 9
ENSDARG00000070480	-2.6	0.000	agr2	anterior gradient 2
ENSDARG00000070041	-2.6	0.001	zgc:153920	zgc:153920

ENSDARG00000040466	-2.6	0.000	vil1	villin 1
ENSDARG00000100352	-2.6	0.000	aglb	amylo-alpha-1, 6-glucosidase, 4-alpha-glucanotransferase b
ENSDARG00000059227	-2.5	0.000	fabp1b.1	fatty acid binding protein 1b, tandem duplicate 1
ENSDARG00000002401	-2.5	0.000	gale	UDP-galactose-4-epimerase
ENSDARG00000061355	-2.5	0.000	aoc1	amine oxidase, copper containing 1
ENSDARG00000038068	-2.5	0.000	ddx5	DEAD (Asp-Glu-Ala-Asp) box helicase 5
ENSDARG00000013871	-2.5	0.000	slc5a1	solute carrier family 5 (sodium/glucose cotransporter), member 1
ENSDARG00000019228	-2.5	0.000	mogat2	monoacylglycerol O-acyltransferase 2
ENSDARG00000038881	-2.5	0.000	acaa2	acetyl-CoA acyltransferase 2
ENSDARG00000062788	-2.5	0.003	irg1l	immunoresponsive gene 1, like
ENSDARG00000093365	-2.5	0.001	si:ch211-226h7.3	si:ch211-226h7.3
ENSDARG00000100635	-2.4	0.000	chia.1	chitinase, acidic.1
ENSDARG00000104919	-2.4	0.003	si:ch211-153b23.3	si:ch211-153b23.3
ENSDARG00000057789	-2.4	0.000	lyz	lysozyme
ENSDARG00000052779	-2.4	0.003	zgc:153932	zgc:153932
ENSDARG00000038834	-2.4	0.000	etfdh	electron-transferring-flavoprotein dehydrogenase
ENSDARG00000103785	-2.4	0.000	sult2st2	sulfotransferase family 2, cytosolic sulfotransferase 2
ENSDARG00000038703	-2.4	0.000	hkdc1	hexokinase domain containing 1
ENSDARG00000105187	-2.4	0.000	gcshb	glycine cleavage system protein H (aminomethyl carrier), b
ENSDARG00000059367	-2.4	0.000	mfap2	microfibrillar-associated protein 2
ENSDARG00000013741	-2.4	0.000	lancl1	LanC antibiotic synthetase component C-like 1 (bacterial)
ENSDARG00000088140	-2.4	0.002	hsd17b7	hydroxysteroid (17-beta) dehydrogenase 7
ENSDARG00000041294	-2.4	0.002	noxo1a	NADPH oxidase organizer 1a
ENSDARG00000030215	-2.4	0.000	matn1	matrilin 1
ENSDARG00000029615	-2.4	0.000	zgc:77056	zgc:77056
ENSDARG00000053554	-2.4	0.000	wdr76	WD repeat domain 76
ENSDARG00000079946	-2.4	0.002	sqlea	squalene epoxidase a
ENSDARG00000043729	-2.4	0.000	plac8.1	placenta-specific 8, tandem duplicate 1
ENSDARG00000091560	-2.3	0.002	slc6a19a.2	solute carrier family 6 (neutral amino acid transporter), member 19a, tandem duplicate 2
ENSDARG00000056203	-2.3	0.003	si:ch211-201h21.5	si:ch211-201h21.5
ENSDARG00000092730	-2.3	0.003	si:dkey-22i16.3	si:dkey-22i16.3
ENSDARG00000069752	-2.3	0.000	ckba	creatine kinase, brain a
ENSDARG00000098237	-2.3	0.003	fbn2b	fibrillin 2b
ENSDARG00000103034	-2.3	0.005	LOC100536500	cysteine-rich venom protein natrin-1-like
ENSDARG00000036993	-2.3	0.000	erbb3b	erb-b2 receptor tyrosine kinase 3b
ENSDARG00000008637	-2.3	0.000	mtp	microsomal triglyceride transfer protein
ENSDARG00000016538	-2.3	0.002	zgc:55888	zgc:55888
ENSDARG00000097826	-2.3	0.002	si:dkey-239b22.2	si:dkey-239b22.2
ENSDARG00000045297	-2.3	0.000	phb2a	prohibitin 2a
ENSDARG00000103672	-2.3	0.000	cirbpa	cold inducible RNA binding protein a

ENSDARG00000006408	-2.3	0.000	hmgb3b	high mobility group box 3b
ENSDARG00000100712	-2.3	0.000	si:dkey-19b23.12	si:dkey-19b23.12
ENSDARG00000030478	-2.3	0.000	zgc:66484	zgc:66484
ENSDARG00000070416	-2.3	0.006	zgc:162816	zgc:162816
ENSDARG00000026759	-2.3	0.000	ldlrb	low density lipoprotein receptor b
ENSDARG00000071377	-2.3	0.004	hsd11b1la	hydroxysteroid (11-beta) dehydrogenase 1-like a
ENSDARG00000022466	-2.3	0.001	clcn5b	chloride channel, voltage-sensitive 5b
ENSDARG00000035872	-2.2	0.000	hsd17b12b	hydroxysteroid (17-beta) dehydrogenase 12b
ENSDARG00000079752	-2.2	0.000	col6a4a	collagen, type VI, alpha 4a
ENSDARG00000031891	-2.2	0.000	tbx1	T-box 1
ENSDARG00000078622	-2.2	0.007	scpp5	secretory calcium-binding phosphoprotein 5
ENSDARG00000043281	-2.2	0.006	stap2b	signal transducing adaptor family member 2b
ENSDARG00000068516	-2.2	0.000	hapln1b	hyaluronan and proteoglycan link protein 1b
ENSDARG00000003219	-2.2	0.000	bin2a	bridging integrator 2a
ENSDARG00000044972	-2.2	0.000	bcap31	B-cell receptor-associated protein 31
ENSDARG00000076146	-2.2	0.007	zgc:172075	zgc:172075
ENSDARG00000079651	-2.2	0.000	si:ch1073-174d20.2	si:ch1073-174d20.2
ENSDARG00000101200	-2.2	0.007	zgc:112964	zgc:112964
ENSDARG00000102477	-2.2	0.001	stard4	StAR-related lipid transfer (START) domain containing 4
ENSDARG00000077169	-2.2	0.012	si:ch211-153b23.4	si:ch211-153b23.4
ENSDARG00000007377	-2.2	0.000	odc1	ornithine decarboxylase 1
ENSDARG00000053526	-2.2	0.003	enpp7.1	ectonucleotide pyrophosphatase/phosphodiesterase 7, tandem duplicate 1
ENSDARG00000101847	-2.2	0.001	si:dkey-185e18.3	si:dkey-185e18.3
ENSDARG00000026629	-2.2	0.000	gmds	GDP-mannose 4,6-dehydratase
ENSDARG00000087625	-2.2	0.008	si:ch211-173a9.7	si:ch211-173a9.7
ENSDARG00000103025	-2.2	0.000	hmgcs1	3-hydroxy-3-methylglutaryl-CoA synthase 1 (soluble)
ENSDARG00000061858	-2.2	0.000	zgc:153968	zgc:153968
ENSDARG00000004748	-2.2	0.000	zgc:100868	zgc:100868
ENSDARG00000101482	-2.2	0.001	hk2	hexokinase 2
ENSDARG00000023712	-2.2	0.000	mao	monoamine oxidase
ENSDARG00000105592	-2.2	0.000	erv	endogenous retrovirus
ENSDARG00000086112	-2.1	0.005	si:ch211-266i6.3	si:ch211-266i6.3
ENSDARG00000068088	-2.1	0.001	tcnl	transcobalamin like
ENSDARG00000089361	-2.1	0.006	wu:fb59d01	wu:fb59d01
ENSDARG00000087873	-2.1	0.000	eevs	2-epi-5-epi-valiolone synthase
ENSDARG00000063177	-2.1	0.001	manf	mesencephalic astrocyte-derived neurotrophic factor
ENSDARG00000061481	-2.1	0.002	zgc:163022	zgc:163022
ENSDARG00000071048	-2.1	0.004	wisp3	WNT1 inducible signaling pathway protein 3
ENSDARG00000077180	-2.1	0.000	slc37a4b	solute carrier family 37 (glucose-6-phosphate transporter), member 4b
ENSDARG00000057035	-2.1	0.001	stoml3b	stomatin (EPB72)-like 3b

ENSDARG00000001760	-2.1	0.004	tnxba	tenascin XBa
ENSDARG00000021242	-2.1	0.000	mvp	major vault protein
ENSDARG00000042708	-2.1	0.000	tuba8l	tubulin, alpha 8 like
ENSDARG00000042544	-2.1	0.001	degs2	delta(4)-desaturase, sphingolipid 2
ENSDARG00000052960	-2.1	0.012	nppa	natriuretic peptide A
ENSDARG00000058638	-2.1	0.005	si:ch211-71m22.1	si:ch211-71m22.1
ENSDARG00000018283	-2.1	0.000	cyba	cytochrome b-245, alpha polypeptide
ENSDARG00000038713	-2.1	0.001	tep1	telomerase-associated protein 1
ENSDARG00000098589	-2.1	0.000	cyb5a	cytochrome b5 type A (microsomal)
ENSDARG00000038185	-2.1	0.013	gh1	growth hormone 1
ENSDARG00000027584	-2.1	0.006	ttpa	tocopherol (alpha) transfer protein
ENSDARG00000053761	-2.1	0.014	si:dkey-187j14.4	si:dkey-187j14.4
ENSDARG00000069559	-2.1	0.010	muc13a	mucin 13a, cell surface associated
ENSDARG00000037654	-2.1	0.005	pmm2	phosphomannomutase 2
ENSDARG00000077045	-2.1	0.009	scg5	secretogranin V
ENSDARG00000003615	-2.1	0.003	slc26a3.2	solute carrier family 26 (anion exchanger), member 3, tandem duplicate 2
ENSDARG00000069116	-2.1	0.000	timm10	translocase of inner mitochondrial membrane 10 homolog (yeast)
ENSDARG00000099960	-2.1	0.011	elovl1a	ELOVL fatty acid elongase 1a
ENSDARG00000039576	-2.1	0.000	fstl1b	follistatin-like 1b
ENSDARG00000099774	-2.1	0.009	cyb5b	cytochrome b5 type B
ENSDARG00000017126	-2.1	0.001	ilvbl	ilvB (bacterial acetolactate synthase)-like
ENSDARG00000002986	-2.1	0.009	gda	guanine deaminase
ENSDARG00000068006	-2.1	0.020	gck	glucokinase (hexokinase 4)
ENSDARG00000074908	-2.1	0.000	col6a1	collagen, type VI, alpha 1
ENSDARG00000075707	-2.1	0.000	nid2a	wu:fb77a09
ENSDARG00000008491	-2.1	0.000	si:ch211-107o10.3	si:ch211-107o10.3
ENSDARG00000020610	-2.1	0.010	tnnt2a	troponin T type 2a (cardiac)
ENSDARG00000092115	-2.1	0.000	eif4a1a	eukaryotic translation initiation factor 4A1A
ENSDARG00000038296	-2.1	0.006	tmem86b	transmembrane protein 86B
ENSDARG00000063333	-2.1	0.001	nup210	nucleoporin 210
ENSDARG00000088366	-2.1	0.000	zgc:77938	zgc:77938
ENSDARG00000074809	-2.0	0.008	zgc:194392	zgc:194392
ENSDARG00000008457	-2.0	0.006	faah2a	fatty acid amide hydrolase 2a
ENSDARG00000024602	-2.0	0.019	zgc:162964	zgc:162964
ENSDARG00000013125	-2.0	0.003	dlx1a	distal-less homeobox 1a
ENSDARG00000042018	-2.0	0.001	fhl2a	four and a half LIM domains 2a
ENSDARG00000022689	-2.0	0.006	itgb1b.2	integrin, beta 1b.2
ENSDARG00000094965	-2.0	0.000	nfil3-5	nuclear factor, interleukin 3 regulated, member 5
ENSDARG00000056008	-2.0	0.000	atp5g1	ATP synthase, H+ transporting, mitochondrial Fo complex, subunit C1 (subunit 9)
ENSDARG00000040747	-2.0	0.000	tm4sf4	transmembrane 4 L six family member 4
ENSDARG00000036161	-2.0	0.000	hnrnpa0l	heterogeneous nuclear ribonucleoprotein A0, like
ENSDARG00000079443	-2.0	0.015	vipb	vasoactive intestinal peptide b
ENSDARG00000104370	-2.0	0.024	esm1	endothelial cell-specific molecule 1

ENSDARG00000029406	-2.0	0.010	h2afx	H2A histone family, member X
ENSDARG00000052905	-2.0	0.021	zgc:165423	zgc:165423
ENSDARG00000012199	-2.0	0.000	gpt2	glutamic pyruvate transaminase (alanine aminotransferase) 2
ENSDARG00000057128	-2.0	0.000	hadhaa	hydroxyacyl-CoA dehydrogenase/3-ketoacyl-CoA thiolase/enoyl-CoA hydratase (trifunctional protein), alpha subunit a
ENSDARG00000039436	-2.0	0.023	il13ra2	interleukin 13 receptor, alpha 2
ENSDARG00000069045	-2.0	0.022	chtf8	CTF8, chromosome transmission fidelity factor 8 homolog (S. cerevisiae)
ENSDARG00000012366	-2.0	0.000	fbp2	fructose-1,6-bisphosphatase 2
ENSDARG00000036291	-2.0	0.001	nucb2b	nucleobindin 2b
ENSDARG00000103825	-2.0	0.012	galnt10	UDP-N-acetyl-alpha-D-galactosamine:polypeptide N-acetylgalactosaminyltransferase 10 (GalNAc-T10)
ENSDARG00000054319	-2.0	0.005	oxct1b	3-oxoacid CoA transferase 1b
ENSDARG00000092044	-2.0	0.009	si:dkey-22f5.9	si:dkey-22f5.9
ENSDARG00000089507	-2.0	0.001	ugt1b5	UDP glucuronosyltransferase 1 family, polypeptide B5
ENSDARG00000075161	-2.0	0.000	defbl1	defensin, beta-like 1
ENSDARG00000097711	-2.0	0.008	si:ch1073-526c11.2	si:ch1073-526c11.2
ENSDARG00000027689	-2.0	0.002	pold1	polymerase (DNA directed), delta 1, catalytic subunit
ENSDARG00000052336	-2.0	0.006	ociad2	OCIA domain containing 2
ENSDARG00000009637	-2.0	0.000	rcvrn3	recoverin 3
ENSDARG00000103163	-2.0	0.000	tomm20a	translocase of outer mitochondrial membrane 20
ENSDARG00000056511	-2.0	0.000	arr3a	arrestin 3a, retinal (X-arrestin)
ENSDARG00000042124	-2.0	0.002	si:dkey-4e7.3	si:dkey-4e7.3
ENSDARG00000055045	-2.0	0.017	casp3b	caspase 3, apoptosis-related cysteine peptidase b
ENSDARG00000051939	-2.0	0.002	pcxb	pyruvate carboxylase b
ENSDARG00000010445	-2.0	0.020	trabd	TraB domain containing
ENSDARG00000062864	-2.0	0.009	gk5	glycerol kinase 5 (putative)
ENSDARG00000068181	-2.0	0.009	dpep1	dipeptidase 1 (renal)
ENSDARG00000076830	-2.0	0.006	si:dkey-65b12.6	si:dkey-65b12.6
ENSDARG00000054632	-2.0	0.003	fli1a	Fli-1 proto-oncogene, ETS transcription factor a
ENSDARG00000015088	-2.0	0.000	dnajb11	DnaJ (Hsp40) homolog, subfamily B, member 11
ENSDARG00000058800	-2.0	0.005	rab25a	RAB25, member RAS oncogene family a
ENSDARG00000060248	-2.0	0.011	FGD4	FYVE, RhoGEF and PH domain-containing protein 4-like
ENSDARG00000053493	-2.0	0.000	aldh1a2	aldehyde dehydrogenase 1 family, member A2
ENSDARG00000035832	-2.0	0.030	pyyb	peptide YYb
ENSDARG00000006215	-2.0	0.000	akr1b1	aldo-keto reductase family 1, member B1 (aldose reductase)
ENSDARG00000007867	-2.0	0.000	dazap2	DAZ associated protein 2
ENSDARG00000073820	-2.0	0.020	zgc:174917	zgc:174917
ENSDARG00000091916	-2.0	0.010	ugt5b2	UDP glucuronosyltransferase 5 family, polypeptide B4

ENSDARG00000030743	-2.0	0.017	sptlc3	serine palmitoyltransferase, long chain base subunit 3
ENSDARG00000098315	-2.0	0.017	cyp1a	cytochrome P450, family 1, subfamily A
ENSDARG00000051957	-2.0	0.001	selm	selenoprotein M
ENSDARG00000002071	-2.0	0.017	adss	adenylosuccinate synthase
ENSDARG00000009844	-2.0	0.011	dusp23a	dual specificity phosphatase 23a
ENSDARG00000024278	-1.9	0.000	adh8b	alcohol dehydrogenase 8b
ENSDARG00000005023	-1.9	0.007	fkbp9	FK506 binding protein 9
ENSDARG00000042856	-1.9	0.010	itpka	inositol-trisphosphate 3-kinase A
ENSDARG00000104901	-1.9	0.000	ostc	oligosaccharyltransferase complex subunit
ENSDARG00000039490	-1.9	0.001	pitpnaa	phosphatidylinositol transfer protein, alpha a
ENSDARG00000074628	-1.9	0.009	si:dkey-10o6.2	si:dkey-10o6.2
ENSDARG00000069018	-1.9	0.011	cyp7a1	cytochrome P450, family 7, subfamily A, polypeptide 1
ENSDARG00000029722	-1.9	0.000	hmgb2a	high mobility group box 2a
ENSDARG00000070427	-1.9	0.000	s100v1	S100 calcium binding protein V1
ENSDARG00000020956	-1.9	0.000	pck2	phosphoenolpyruvate carboxykinase 2 (mitochondrial)
ENSDARG00000100353	-1.9	0.010	si:ch1073-398f15.1	si:ch1073-398f15.1
ENSDARG00000070046	-1.9	0.006	eny2	enhancer of yellow 2 homolog (Drosophila)
ENSDARG00000099558	-1.9	0.037	NPC1L1	NPC1 like intracellular cholesterol transporter 1
ENSDARG00000038056	-1.9	0.001	fgfbp2b	fibroblast growth factor binding protein 2b
ENSDARG00000090185	-1.9	0.000	si:dkeyp-73b11.8	si:dkeyp-73b11.8
ENSDARG00000102442	-1.9	0.006	zgc:165502	zgc:165502
ENSDARG00000088432	-1.9	0.049	si:dkey-162h11.3	si:dkey-162h11.3
ENSDARG00000075261	-1.9	0.028	timp2b	TIMP metallopeptidase inhibitor 2b
ENSDARG00000097513	-1.9	0.003	CT573383.1	CT573383.1
ENSDARG00000095417	-1.9	0.011	or125-4	odorant receptor, family E, subfamily 125, member 4
ENSDARG00000003941	-1.9	0.008	rrs1	RRS1 ribosome biogenesis regulator homolog (S. cerevisiae)
ENSDARG00000103443	-1.9	0.000	vwde	von Willebrand factor D and EGF domains
ENSDARG00000057706	-1.9	0.002	si:ch211-137i24.10	si:ch211-137i24.10
ENSDARG00000087640	-1.9	0.000	nsfl1c	NSFL1 (p97) cofactor (p47)
ENSDARG00000016393	-1.9	0.000	arf2b	ADP-ribosylation factor 2b
ENSDARG00000095796	-1.9	0.001	si:dkey-87o1.2	si:dkey-87o1.2
ENSDARG00000052437	-1.9	0.000	mia	melanoma inhibitory activity
ENSDARG00000057910	-1.9	0.000	mrps34	mitochondrial ribosomal protein S34
ENSDARG00000038010	-1.9	0.000	rac2	ras-related C3 botulinum toxin substrate 2 (rho family, small GTP binding protein Rac2)
ENSDARG00000057504	-1.9	0.010	si:dkey-202g17.3	si:dkey-202g17.3
ENSDARG00000027807	-1.9	0.012	fynrk	fyn-related Src family tyrosine kinase
ENSDARG00000061621	-1.9	0.026	them4	thioesterase superfamily member 4
ENSDARG00000003931	-1.9	0.000	cndp2	CNDP dipeptidase 2 (metallopeptidase M20 family)
ENSDARG00000096454	-1.9	0.002	ap1m2	adaptor-related protein complex 1, mu 2 subunit
ENSDARG00000019838	-1.9	0.004	ugdh	UDP-glucose 6-dehydrogenase

ENSDARG00000008305	-1.9	0.022	hand2	heart and neural crest derivatives expressed 2
ENSDARG00000093535	-1.9	0.001	si:ch211-265m20.8	si:ch211-265m20.8
ENSDARG00000019532	-1.9	0.006	fads2	fatty acid desaturase 2
ENSDARG00000020866	-1.9	0.000	apoa4b.2	apolipoprotein A-IV b, tandem duplicate 2
ENSDARG00000100792	-1.9	0.011	zgc:154142	zgc:154142
ENSDARG00000102798	-1.9	0.000	mcm2	minichromosome maintenance complex component 2
ENSDARG00000009544	-1.9	0.000	cldnb	claudin b
ENSDARG00000091116	-1.9	0.007	pkhd1l1	polycystic kidney and hepatic disease 1 (autosomal recessive)-like 1
ENSDARG00000095832	-1.9	0.009	si:ch73-269m23.4	si:ch73-269m23.4
ENSDARG00000003259	-1.9	0.030	loxa	lysyl oxidase a
ENSDARG00000028106	-1.9	0.000	glrx	glutaredoxin (thioltransferase)
ENSDARG00000099175	-1.9	0.000	hmgb1a	high mobility group box 1a
ENSDARG00000055540	-1.9	0.017	pfkfb4a	6-phosphofructo-2-kinase/fructose-2,6-biphosphatase 4a
ENSDARG00000006029	-1.9	0.001	lta4h	leukotriene A4 hydrolase
ENSDARG00000103369	-1.9	0.000	taldo1	transaldolase 1
ENSDARG00000069261	-1.9	0.037	metap2a	methionyl aminopeptidase 2a
ENSDARG00000038785	-1.9	0.002	abcf2a	ATP-binding cassette, sub-family F (GCN20), member 2a
ENSDARG00000003869	-1.9	0.005	decr1	2,4-dienoyl CoA reductase 1, mitochondrial
ENSDARG00000005112	-1.9	0.000	cdh17	cadherin 17, LI cadherin (liver-intestine)
ENSDARG00000097040	-1.9	0.000	si:dkey-146l2.3	si:dkey-146l2.3
ENSDARG00000057983	-1.9	0.028	svopl	SVOP-like
ENSDARG00000058556	-1.9	0.004	muc5.2	mucin 5.2
ENSDARG00000053068	-1.9	0.037	cyp8b1	cytochrome P450, family 8, subfamily B, polypeptide 1
ENSDARG00000055591	-1.9	0.037	pipox	pipecolic acid oxidase
ENSDARG00000095464	-1.9	0.010	gstt2	glutathione S-transferase theta 2
ENSDARG00000105188	-1.9	0.000	si:dkey-76i15.1	si:dkey-76i15.1
ENSDARG00000011770	-1.9	0.004	dhrs12	dehydrogenase/reductase (SDR family) member 12
ENSDARG00000018491	-1.9	0.001	pdia4	protein disulfide isomerase family A, member 4
ENSDARG00000022303	-1.9	0.000	higd1a	HIG1 hypoxia inducible domain family, member 1A
ENSDARG00000079932	-1.9	0.001	zgc:152830	zgc:152830
ENSDARG00000028816	-1.9	0.005	tmed3	transmembrane p24 trafficking protein 3
ENSDARG00000020679	-1.8	0.000	clpp	caseinolytic mitochondrial matrix peptidase proteolytic subunit
ENSDARG00000007480	-1.8	0.000	rpe65a	retinal pigment epithelium-specific protein 65a
ENSDARG00000092110	-1.8	0.000	si:ch1073-470i20.2	si:ch1073-470i20.2
ENSDARG00000097533	-1.8	0.000	si:dkey-29l4.4	si:dkey-29l4.4
ENSDARG00000021135	-1.8	0.005	dhrs4	dehydrogenase/reductase (SDR family) member 4
ENSDARG00000090969	-1.8	0.041	cbln18	cerebellin 18
ENSDARG00000012903	-1.8	0.024	slc34a2a	solute carrier family 34 (type II sodium/phosphate cotransporter), member 2a
ENSDARG00000053405	-1.8	0.022	sord	sorbitol dehydrogenase
ENSDARG00000009488	-1.8	0.002	gipc2	GIPC PDZ domain containing family, member 2

ENSDARG00000103488	-1.8	0.002	si:ch211-107o10.4	si:ch211-107o10.4
ENSDARG00000068515	-1.8	0.001	chs1	chitin synthase 1
ENSDARG00000014031	-1.8	0.004	abcc2	ATP-binding cassette, sub-family C (CFTR/MRP), member 2
ENSDARG00000033273	-1.8	0.048	primpol	primase and polymerase (DNA-directed)
ENSDARG00000077313	-1.8	0.027	slc46a3	solute carrier family 46, member 3
ENSDARG00000025375	-1.8	0.000	idh1	isocitrate dehydrogenase 1 (NADP+), soluble
ENSDARG00000100315	-1.8	0.025	slc15a1a	solute carrier family 15 (oligopeptide transporter), member 1a
ENSDARG00000037640	-1.8	0.010	aurkb	aurora kinase B
ENSDARG00000020364	-1.8	0.000	fbp1b	fructose-1,6-bisphosphatase 1b
ENSDARG00000057064	-1.8	0.010	enpep	glutamyl aminopeptidase
ENSDARG00000002197	-1.8	0.003	pygl	phosphorylase
ENSDARG00000017985	-1.8	0.007	zgc:77739	zgc:77739
ENSDARG00000097821	-1.8	0.045	si:dkey-244a7.2	si:dkey-244a7.2
ENSDARG00000013794	-1.8	0.001	klf11b	Kruppel-like factor 11b
ENSDARG00000061436	-1.8	0.000	col6a2	collagen, type VI, alpha 2
ENSDARG00000030844	-1.8	0.001	klf11a	Kruppel-like factor 11a
ENSDARG00000076192	-1.8	0.000	ankrd1b	ankyrin repeat domain 1b (cardiac muscle)
ENSDARG00000053133	-1.8	0.032	b3gnt7l	UDP-GlcNAc:betaGal beta-1,3-N-acetylglucosaminyltransferase 7, like
ENSDARG00000091574	-1.8	0.047	suox	sulfite oxidase
ENSDARG00000016304	-1.8	0.015	tfdp1b	transcription factor Dp-1, b
ENSDARG00000024717	-1.8	0.002	selenbp1	selenium binding protein 1
ENSDARG00000013670	-1.8	0.004	hyou1	hypoxia up-regulated 1
ENSDARG00000011521	-1.8	0.034	upb1	ureidopropionase, beta
ENSDARG00000071021	-1.8	0.010	papss2a	3'-phosphoadenosine 5'-phosphosulfate synthase 2a
ENSDARG00000076526	-1.8	0.000	gar1	GAR1 homolog, ribonucleoprotein
ENSDARG00000041493	-1.8	0.015	ipo4	importin 4
ENSDARG00000089863	-1.8	0.031	ccdc57	coiled-coil domain containing 57
ENSDARG00000099448	-1.8	0.001	sh3d21	SH3 domain containing 21
ENSDARG00000044691	-1.8	0.040	ppp1r3b	protein phosphatase 1, regulatory subunit 3B
ENSDARG00000045414	-1.8	0.027	elovl2	ELOVL fatty acid elongase 2
ENSDARG00000038225	-1.8	0.001	nras	neuroblastoma RAS viral (v-ras) oncogene homolog
ENSDARG00000062795	-1.8	0.002	abcb7	ATP-binding cassette, sub-family B (MDR/TAP), member 7
ENSDARG00000023498	-1.8	0.017	gmppab	GDP-mannose pyrophosphorylase Ab
ENSDARG00000019128	-1.8	0.004	tpm4b	tropomyosin 4b
ENSDARG00000028275	-1.8	0.006	sult1st1	sulfotransferase family 1, cytosolic sulfotransferase 1
ENSDARG00000097553	-1.8	0.003	si:dkey-193e13.8	si:dkey-193e13.8
ENSDARG00000058953	-1.8	0.025	abcc4	ATP-binding cassette, sub-family C (CFTR/MRP), member 4
ENSDARG00000090548	-1.8	0.041	CR753886.1	GTPase IMAP family member 4-like
ENSDARG00000102097	-1.8	0.007	nfkbiz	nuclear factor of kappa light polypeptide gene enhancer in B-cells inhibitor, zeta

ENSDARG00000074852	-1.8	0.026	myo15b	myosin XVB
ENSDARG00000070833	-1.8	0.024	lin52	lin-52 DREAM MuvB core complex component
ENSDARG00000039669	-1.8	0.004	zgc:100864	zgc:100864
ENSDARG00000017794	-1.8	0.009	sdr16c5b	short chain dehydrogenase/reductase family 16C, member 5b
ENSDARG00000045946	-1.8	0.036	sec24d	SEC24 homolog D, COPII coat complex component
ENSDARG00000052708	-1.8	0.013	tnni1b	troponin I type 1b (skeletal, slow)
ENSDARG00000011404	-1.8	0.009	fen1	flap structure-specific endonuclease 1
ENSDARG00000087198	-1.8	0.016	cthrc1a	collagen triple helix repeat containing 1a
ENSDARG00000099148	-1.8	0.001	bzw1b	basic leucine zipper and W2 domains 1b
ENSDARG00000095633	-1.8	0.002	si:ch211-133l5.7	si:ch211-133l5.7
ENSDARG00000100265	-1.8	0.048	rhcgb	Rh family, C glycoprotein b
ENSDARG00000045051	-1.8	0.003	slc16a3	solute carrier family 16 (monocarboxylate transporter), member 3
ENSDARG00000027966	-1.8	0.024	ap1s3b	adaptor-related protein complex 1, sigma 3 subunit, b
ENSDARG00000036846	-1.8	0.019	anks4b	ankyrin repeat and sterile alpha motif domain containing 4B
ENSDARG00000070487	-1.8	0.038	zgc:110783	zgc:110783
ENSDARG00000099185	-1.8	0.000	chia.2	chitinase, acidic.2
ENSDARG00000043242	-1.8	0.007	si:dkey-222f2.1	si:dkey-222f2.1
ENSDARG00000069615	-1.8	0.000	ckmt2a	creatine kinase, mitochondrial 2a
ENSDARG00000012399	-1.8	0.000	adssl	adenylosuccinate synthase, like
ENSDARG00000017775	-1.8	0.000	atp5g3a	ATP synthase, H+ transporting, mitochondrial Fo complex, subunit C3 (subunit 9), genome duplicate a
ENSDARG00000069099	-1.8	0.002	tmco1	transmembrane and coiled-coil domains 1
ENSDARG00000037846	-1.8	0.002	hm13	histocompatibility (minor) 13
ENSDARG00000086896	-1.8	0.001	fubp3	far upstream element (FUSE) binding protein 3
ENSDARG00000021239	-1.8	0.010	apaf1	apoptotic peptidase activating factor 1
ENSDARG00000033466	-1.8	0.000	tagln2	transgelin 2
ENSDARG00000074745	-1.7	0.000	zmp:0000000760	zmp:0000000760
ENSDARG00000017874	-1.7	0.014	dnajc3b	DnaJ (Hsp40) homolog, subfamily C, member 3b
ENSDARG00000019398	-1.7	0.001	psma6a	proteasome subunit alpha 6a
ENSDARG00000019976	-1.7	0.003	idi1	isopentenyl-diphosphate delta isomerase 1
ENSDARG00000061375	-1.7	0.000	sgpl1	sphingosine-1-phosphate lyase 1
ENSDARG00000018146	-1.7	0.000	gpx1a	glutathione peroxidase 1a
ENSDARG00000009401	-1.7	0.008	vcanb	versican b
ENSDARG00000036463	-1.7	0.048	cldn15a	claudin 15a
ENSDARG00000097902	-1.7	0.003	si:ch73-204p21.2	si:ch73-204p21.2
ENSDARG00000101986	-1.7	0.010	irf6	interferon regulatory factor 6
ENSDARG00000002405	-1.7	0.003	si:ch211-225b11.1	si:ch211-225b11.1
ENSDARG00000007285	-1.7	0.006	mrpl57	mitochondrial ribosomal protein L57
ENSDARG00000103404	-1.7	0.003	uchl5	ubiquitin carboxyl-terminal hydrolase L5
ENSDARG00000093044	-1.7	0.032	si:ch211-161h7.5	si:ch211-161h7.5
ENSDARG00000097351	-1.7	0.001	flnbl	filamin B, like

ENSDARG00000000069	-1.7	0.000	dap	death-associated protein
ENSDARG00000019998	-1.7	0.009	slc30a7	solute carrier family 30 (zinc transporter), member 7
ENSDARG00000053912	-1.7	0.000	fbl	fibrillarin
ENSDARG00000102888	-1.7	0.038	gpr39	G protein-coupled receptor 39
ENSDARG00000009342	-1.7	0.004	txndc5	thioredoxin domain containing 5
ENSDARG00000102808	-1.7	0.031	calr3b	calreticulin 3b
ENSDARG00000029695	-1.7	0.025	pgp	phosphoglycolate phosphatase
ENSDARG00000034916	-1.7	0.001	hat1	histone acetyltransferase 1
ENSDARG00000020944	-1.7	0.001	ezra	ezrin a
ENSDARG00000030765	-1.7	0.000	hadh	hydroxyacyl-CoA dehydrogenase
ENSDARG00000091656	-1.7	0.016	lsm8	LSM8 homolog, U6 small nuclear RNA associated
ENSDARG00000012060	-1.7	0.016	thbs3b	thrombospondin 3b
ENSDARG00000044261	-1.7	0.002	si:ch211-243g18.2	si:ch211-243g18.2
ENSDARG00000068851	-1.7	0.005	rnf183	ring finger protein 183
ENSDARG00000058605	-1.7	0.024	vsig10	V-set and immunoglobulin domain containing 10
ENSDARG00000090228	-1.7	0.000	gsta.1	glutathione S-transferase, alpha tandem duplicate 1
ENSDARG00000030781	-1.7	0.003	acads	acyl-CoA dehydrogenase, C-2 to C-3 short chain
ENSDARG00000075445	-1.7	0.000	psmb5	proteasome subunit beta 5
ENSDARG00000017811	-1.7	0.002	si:ch211-210c8.6	si:ch211-210c8.6
ENSDARG00000079255	-1.7	0.031	zgc:174935	zgc:174935
ENSDARG00000091222	-1.7	0.046	si:dkey-190l8.2	solute carrier family 22 member 8-like
ENSDARG00000038618	-1.7	0.008	cpt2	carnitine palmitoyltransferase 2
ENSDARG00000029795	-1.7	0.000	fam213b	family with sequence similarity 213, member B
ENSDARG00000040683	-1.7	0.030	si:ch73-40a17.3	si:ch73-40a17.3
ENSDARG00000092337	-1.7	0.040	gas5	growth arrest-specific 5
ENSDARG00000043684	-1.7	0.041	bpnt1	bisphosphate nucleotidase 1
ENSDARG00000005423	-1.7	0.000	pgam1a	phosphoglycerate mutase 1a
ENSDARG00000097118	-1.7	0.000	si:dkey-33c14.3	si:dkey-33c14.3
ENSDARG00000100585	-1.7	0.003	coa3a	cytochrome C oxidase assembly factor 3a
ENSDARG00000101629	-1.7	0.010	mgam	maltase-glucoamylase
ENSDARG00000042296	-1.7	0.024	dlx5a	distal-less homeobox 5a
ENSDARG00000093219	-1.7	0.003	si:ch211-152f6.7	si:ch211-152f6.7
ENSDARG00000044402	-1.7	0.013	nop16	NOP16 nucleolar protein homolog (yeast)
ENSDARG00000020187	-1.7	0.000	zgc:92027	zgc:92027
ENSDARG00000026979	-1.7	0.027	krt1-c5	keratin, type 1, gene c5
ENSDARG00000037361	-1.7	0.002	kdelr2b	KDEL (Lys-Asp-Glu-Leu) endoplasmic reticulum protein retention receptor 2b
ENSDARG00000006260	-1.7	0.000	tuba8l4	tubulin, alpha 8 like 4
ENSDARG00000105408	-1.7	0.001	si:ch211-237b12.4	si:ch211-237b12.4
ENSDARG00000009215	-1.7	0.039	zgc:112437	zgc:112437
ENSDARG00000017676	-1.7	0.000	mmp2	matrix metallopeptidase 2
ENSDARG00000018002	-1.7	0.003	eci1	enoyl-CoA delta isomerase 1
ENSDARG00000071014	-1.7	0.001	s100u	S100 calcium binding protein U
ENSDARG00000101861	-1.7	0.041	cyp2k19	cytochrome P450, family 2, subfamily k, polypeptide 19

ENSDARG00000103226	-1.7	0.048	dhcr7	7-dehydrocholesterol reductase
ENSDARG00000042876	-1.7	0.005	abracl	ABRA C-terminal like
ENSDARG00000103759	-1.7	0.033	fahd2a	fumarylacetoacetate hydrolase domain containing 2A
ENSDARG00000060594	-1.7	0.000	hadhab	hydroxyacyl-CoA dehydrogenase/3-ketoacyl-CoA thiolase/enoyl-CoA hydratase (trifunctional protein), alpha subunit b
ENSDARG00000043237	-1.7	0.049	nfil3-2	nuclear factor, interleukin 3 regulated, member 2
ENSDARG00000045254	-1.7	0.030	C7orf50	chromosome 24 open reading frame, human C7orf50
ENSDARG00000099291	-1.7	0.014	lsr	lipolysis stimulated lipoprotein receptor
ENSDARG00000055876	-1.7	0.017	msmo1	methylsterol monooxygenase 1
ENSDARG00000104085	-1.7	0.002	abcd3a	ATP-binding cassette, sub-family D (ALD), member 3a
ENSDARG00000031929	-1.7	0.011	stard14	START domain containing 14
ENSDARG00000099705	-1.7	0.040	foxred1	FAD-dependent oxidoreductase domain containing 1
ENSDARG00000096533	-1.7	0.007	rltgr	RAMP-like triterpene glycoside receptor
ENSDARG00000100558	-1.7	0.011	slbp	stem-loop binding protein
ENSDARG00000004251	-1.7	0.028	dhfr	dihydrofolate reductase
ENSDARG00000092099	-1.7	0.030	si:ch211-133n4.6	si:ch211-133n4.6
ENSDARG00000103659	-1.7	0.046	bco1l	beta-carotene oxygenase 1, like
ENSDARG00000005058	-1.7	0.022	ncapd2	non-SMC condensin I complex, subunit D2
ENSDARG00000010246	-1.7	0.000	prmt1	protein arginine methyltransferase 1
ENSDARG00000005122	-1.7	0.000	atp2a2b	ATPase, Ca++ transporting, cardiac muscle, slow twitch 2b
ENSDARG00000021149	-1.7	0.001	cbr1l	carbonyl reductase 1-like
ENSDARG00000096922	-1.7	0.003	si:ch211-147k10.6	si:ch211-147k10.6
ENSDARG00000045091	-1.7	0.033	mrpl10	mitochondrial ribosomal protein L10
ENSDARG00000033285	-1.7	0.000	gsto2	glutathione S-transferase omega 2
ENSDARG00000032639	-1.7	0.002	cd36	CD36 molecule (thrombospondin receptor)
ENSDARG00000056665	-1.7	0.016	nsun2	NOP2/Sun RNA methyltransferase family, member 2
ENSDARG00000038213	-1.7	0.005	slc35b1	solute carrier family 35, member B1
ENSDARG00000058088	-1.7	0.000	aifm1	apoptosis-inducing factor, mitochondrion-associated 1
ENSDARG00000076221	-1.7	0.008	zgc:198419	zgc:198419
ENSDARG00000016496	-1.7	0.027	cdk8	cyclin-dependent kinase 8
ENSDARG00000079847	-1.7	0.048	zgc:194578	zgc:194578
ENSDARG00000017744	-1.7	0.017	smc2	structural maintenance of chromosomes 2
ENSDARG00000039423	-1.7	0.024	si:ch211-217g15.3	si:ch211-217g15.3
ENSDARG00000008982	-1.7	0.000	casq2	calsequestrin 2
ENSDARG00000101900	-1.7	0.000	xrn2	5'-3' exoribonuclease 2
ENSDARG00000079119	-1.7	0.016	si:ch211-229d2.5	si:ch211-229d2.5
ENSDARG00000013776	-1.7	0.010	thop1	thimet oligopeptidase 1
ENSDARG00000020454	-1.7	0.001	psmd8	proteasome 26S subunit, non-ATPase 8
ENSDARG00000071429	-1.7	0.007	tdo2a	tryptophan 2,3-dioxygenase a

ENSDARG00000063627	-1.7	0.010	bop1	block of proliferation 1
ENSDARG00000078069	-1.7	0.017	rrm2	ribonucleotide reductase M2 polypeptide
ENSDARG00000070651	-1.7	0.002	prkcdb	protein kinase C, delta b
ENSDARG00000012450	-1.7	0.010	vmp1	vacuole membrane protein 1
ENSDARG00000044167	-1.6	0.003	padi2	peptidyl arginine deiminase, type II
ENSDARG00000020494	-1.6	0.002	znf330	zinc finger protein 330
ENSDARG00000056186	-1.6	0.000	eif5a2	eukaryotic translation initiation factor 5A2
ENSDARG00000045167	-1.6	0.041	dlgap5	discs, large (Drosophila) homolog-associated protein 5
ENSDARG00000097209	-1.6	0.031	cox8b	cytochrome c oxidase subunit 8b
ENSDARG00000093864	-1.6	0.041	wee1	WEE1 G2 checkpoint kinase
ENSDARG00000078785	-1.6	0.002	tmem258	transmembrane protein 258
ENSDARG00000035631	-1.6	0.029	sdf2l1	stromal cell-derived factor 2-like 1
ENSDARG00000034643	-1.6	0.037	fhl3a	four and a half LIM domains 3a
ENSDARG00000089187	-1.6	0.045	wfdc2	WAP four-disulfide core domain 2
ENSDARG00000052734	-1.6	0.041	hmgcra	3-hydroxy-3-methylglutaryl-CoA reductase a
ENSDARG00000040401	-1.6	0.003	slc25a20	solute carrier family 25 (carnitine/acylcarnitine translocase), member 20
ENSDARG00000063438	-1.6	0.002	srebf2	sterol regulatory element binding transcription factor 2
ENSDARG00000042977	-1.6	0.005	nfil3	nuclear factor, interleukin 3 regulated
ENSDARG00000101877	-1.6	0.021	rbm34	RNA binding motif protein 34
ENSDARG00000011418	-1.6	0.017	sigmar1	sigma non-opioid intracellular receptor 1
ENSDARG00000011890	-1.6	0.006	cry4	cryptochrome circadian clock 4
ENSDARG00000016855	-1.6	0.029	sf3b5	splicing factor 3b, subunit 5
ENSDARG00000089362	-1.6	0.041	grn1	granulin 1
ENSDARG00000093957	-1.6	0.016	si:dkey-251i10.2	si:dkey-251i10.2
ENSDARG00000013763	-1.6	0.005	rrbp1a	ribosome binding protein 1a
ENSDARG00000018574	-1.6	0.015	sf3b4	splicing factor 3b, subunit 4
ENSDARG00000070846	-1.6	0.033	dazap1	DAZ associated protein 1
ENSDARG00000090847	-1.6	0.049	si:ch211-209l18.4	si:ch211-209l18.4
ENSDARG00000012422	-1.6	0.000	col11a2	collagen, type XI, alpha 2
ENSDARG00000018178	-1.6	0.011	pgm2	phosphoglucomutase 2
ENSDARG00000014793	-1.6	0.002	ehd1b	EH-domain containing 1b
ENSDARG00000093303	-1.6	0.045	ifitm1	interferon induced transmembrane protein 1
ENSDARG00000011958	-1.6	0.033	ctnnbl1	catenin, beta like 1
ENSDARG00000000068	-1.6	0.022	slc9a3r1	solute carrier family 9, subfamily A (NHE3, cation proton antiporter 3), member 3 regulator 1
ENSDARG00000040158	-1.6	0.000	cdc42l	cell division cycle 42, like
ENSDARG00000101214	-1.6	0.003	pkd1l2b	polycystic kidney disease 1 like 2b
ENSDARG00000063624	-1.6	0.017	gfm1	G elongation factor, mitochondrial 1
ENSDARG00000011459	-1.6	0.020	gsna	gelsolin a
ENSDARG00000079095	-1.6	0.041	rin1a	Ras and Rab interactor 1a
ENSDARG00000019096	-1.6	0.020	myl7	myosin, light chain 7, regulatory
ENSDARG00000008936	-1.6	0.016	sec11a	SEC11 homolog A, signal peptidase complex subunit

ENSDARG00000045814	-1.6	0.005	samm50	SAMM50 sorting and assembly machinery component
ENSDARG00000098853	-1.6	0.004	ehd1a	EH-domain containing 1a
ENSDARG00000056087	-1.6	0.033	ecrg4a	esophageal cancer related gene 4a
ENSDARG00000092155	-1.6	0.001	apoc2	apolipoprotein C-II
ENSDARG00000099664	-1.6	0.007	sep15	selenoprotein 15
ENSDARG00000044090	-1.6	0.017	zmpste24	zinc metallopeptidase, STE24 homolog
ENSDARG00000037307	-1.6	0.048	gnpda1	glucosamine-6-phosphate deaminase 1
ENSDARG00000073699	-1.6	0.000	col9a1a	collagen, type IX, alpha 1a
ENSDARG00000036625	-1.6	0.006	polr2f	polymerase (RNA) II (DNA directed) polypeptide F
ENSDARG00000054362	-1.6	0.001	ccdc47	coiled-coil domain containing 47
ENSDARG00000045199	-1.6	0.039	zgc:55262	zgc:55262
ENSDARG00000060282	-1.6	0.025	rnf121	ring finger protein 121
ENSDARG00000004687	-1.6	0.033	acaa1	acetyl-CoA acyltransferase 1
ENSDARG00000092976	-1.6	0.037	si:ch211-127i16.2	si:ch211-127i16.2
ENSDARG00000075178	-1.6	0.014	lpcat3	lysophosphatidylcholine acyltransferase 3
ENSDARG00000104138	-1.6	0.009	igfbp7	insulin-like growth factor binding protein 7
ENSDARG00000075570	-1.6	0.002	ide	insulin-degrading enzyme
ENSDARG00000057672	-1.6	0.024	plpp5	phospholipid phosphatase 5
ENSDARG00000036893	-1.6	0.041	f13a1b	coagulation factor XIII, A1 polypeptide b
ENSDARG00000012871	-1.6	0.001	npepl1	aminopeptidase-like 1
ENSDARG00000101485	-1.6	0.014	si:ch1073-469d17.2	si:ch1073-469d17.2
ENSDARG00000104818	-1.6	0.011	si:ch211-93f2.1	si:ch211-93f2.1
ENSDARG00000029230	-1.6	0.045	pnp4b	purine nucleoside phosphorylase 4b
ENSDARG00000023299	-1.6	0.033	snu13b	SNU13 homolog, small nuclear ribonucleoprotein b (U4/U6.U5)
ENSDARG00000005675	-1.6	0.021	sec61a1l	Sec61 translocon alpha 1 subunit, like
ENSDARG00000100510	-1.6	0.000	actr3	ARP3 actin-related protein 3 homolog (yeast)
ENSDARG00000078592	-1.6	0.010	nomo	nodal modulator
ENSDARG00000025826	-1.6	0.041	gna12a	guanine nucleotide binding protein (G protein) alpha 12a
ENSDARG00000089769	-1.6	0.013	hapln1a	hyaluronan and proteoglycan link protein 1a
ENSDARG00000018936	-1.6	0.021	zcchc17	zinc finger, CCHC domain containing 17
ENSDARG00000026369	-1.6	0.000	dbi	diazepam binding inhibitor (GABA receptor modulator, acyl-CoA binding protein)
ENSDARG00000071336	-1.6	0.032	mif	macrophage migration inhibitory factor
ENSDARG00000098746	-1.6	0.004	dhrs13l1	dehydrogenase/reductase (SDR family) member 13 like 1
ENSDARG00000020984	-1.6	0.005	slc16a10	solute carrier family 16 (aromatic amino acid transporter), member 10
ENSDARG00000035326	-1.6	0.009	nccrp1	non-specific cytotoxic cell receptor protein 1
ENSDARG00000043976	-1.6	0.000	etf1b	eukaryotic translation termination factor 1b
ENSDARG00000019260	-1.6	0.040	dhrs9	dehydrogenase/reductase (SDR family) member 9
ENSDARG00000043561	-1.6	0.002	psmc1b	proteasome 26S subunit, ATPase 1b
ENSDARG00000057714	-1.6	0.022	cmah	cytidine monophospho-N-acetylneuraminic acid hydroxylase
ENSDARG00000036422	-1.6	0.039	ntn5	netrin 5

ENSDARG00000035882	-1.6	0.016	hrsp12	heat-responsive protein 12
ENSDARG00000092112	-1.6	0.001	tomm40	translocase of outer mitochondrial membrane 40 homolog (yeast)
ENSDARG00000029931	-1.6	0.034	atp13a1	ATPase type 13A1
ENSDARG00000025094	-1.6	0.017	edem1	ER degradation enhancer, mannosidase alpha-like 1
ENSDARG00000052170	-1.6	0.030	uap1	UDP-N-acetylglucosamine pyrophosphorylase 1
ENSDARG00000090063	-1.6	0.044	fa2h	fatty acid 2-hydroxylase
ENSDARG00000068710	-1.6	0.001	nid1a	nidogen 1a
ENSDARG00000062756	-1.6	0.032	pllp	plasmolipin
ENSDARG00000100133	-1.6	0.001	lect1	leukocyte cell derived chemotaxin 1
ENSDARG00000097294	-1.6	0.011	si:ch211-247i17.6	si:ch211-247i17.6
ENSDARG00000035622	-1.6	0.000	xbp1	X-box binding protein 1
ENSDARG00000010010	-1.6	0.001	trim13	tripartite motif containing 13
ENSDARG00000029439	-1.6	0.009	atp2a2a	ATPase, Ca++ transporting, cardiac muscle, slow twitch 2a
ENSDARG00000054137	-1.6	0.043	adgrg6	adhesion G protein-coupled receptor G6
ENSDARG00000061301	-1.6	0.016	gmpr2	guanosine monophosphate reductase 2
ENSDARG00000013333	-1.6	0.000	ndufa10	NADH dehydrogenase (ubiquinone) 1 alpha subcomplex, 10
ENSDARG00000087402	-1.6	0.000	zgc:171719	tropomyosin 1 (alpha)
ENSDARG00000017004	-1.6	0.049	myo10	myosin X
ENSDARG00000045574	-1.6	0.050	lrmp	lymphoid-restricted membrane protein
ENSDARG00000101324	-1.6	0.003	apoa1b	apolipoprotein A-Ib
ENSDARG00000087779	-1.6	0.022	pum3	im:7148292
ENSDARG00000037845	-1.6	0.000	col9a3	collagen, type IX, alpha 3
ENSDARG00000021309	-1.6	0.011	rhoca	ras homolog family member Ca
ENSDARG00000016484	-1.6	0.017	dkc1	dyskeratosis congenita 1, dyskerin
ENSDARG00000053990	-1.6	0.000	hmgb2b	high mobility group box 2b
ENSDARG00000004261	-1.6	0.001	tmed9	transmembrane p24 trafficking protein 9
ENSDARG00000005085	-1.6	0.001	ggctb	gamma-glutamylcyclotransferase b
ENSDARG00000024209	-1.6	0.013	wasf2	WAS protein family, member 2
ENSDARG00000070358	-1.6	0.037	smim12	small integral membrane protein 12
ENSDARG00000070386	-1.6	0.049	krtcap2	keratinocyte associated protein 2
ENSDARG00000027582	-1.6	0.016	angptl7	angiopoietin-like 7
ENSDARG00000043128	-1.6	0.008	cldne	claudin e
ENSDARG00000079305	-1.6	0.000	hbae3	hemoglobin alpha embryonic-3
ENSDARG00000102417	-1.6	0.003	psmd7	proteasome 26S subunit, non-ATPase 7
ENSDARG00000057853	-1.6	0.000	atp6v0ca	ATPase, H+ transporting, lysosomal, V0 subunit ca
ENSDARG00000043511	-1.6	0.002	prdx6	peroxiredoxin 6
ENSDARG00000023963	-1.6	0.001	tpm4a	tropomyosin 4a
ENSDARG00000044914	-1.6	0.002	suclg2	succinate-CoA ligase, GDP-forming, beta subunit
ENSDARG00000015343	-1.6	0.003	pgd	phosphogluconate dehydrogenase
ENSDARG00000103428	-1.6	0.019	ogdhb	oxoglutarate (alpha-ketoglutarate) dehydrogenase b (lipoamide)
ENSDARG00000027424	-1.6	0.018	slc25a3a	solute carrier family 25 (mitochondrial carrier
ENSDARG00000058730	-1.6	0.004	rdh10a	retinol dehydrogenase 10a

ENSDARG00000020072	-1.6	0.000	thbs4b	thrombospondin 4b
ENSDARG00000035858	-1.6	0.003	cnn2	calponin 2
ENSDARG00000069375	-1.5	0.024	zgc:162608	zgc:162608
ENSDARG00000060041	-1.5	0.030	lig1	ligase I, DNA, ATP-dependent
ENSDARG00000031587	-1.5	0.038	flvcr1	feline leukemia virus subgroup C cellular receptor 1
ENSDARG00000092546	-1.5	0.001	pdap1a	pdgfa associated protein 1a
ENSDARG00000012729	-1.5	0.029	hcls1	hematopoietic cell-specific Lyn substrate 1
ENSDARG00000018124	-1.5	0.001	psmd3	proteasome 26S subunit, non-ATPase 3
ENSDARG00000061985	-1.5	0.005	rbm47	RNA binding motif protein 47
ENSDARG00000020702	-1.5	0.018	odf2b	outer dense fiber of sperm tails 2b
ENSDARG00000006588	-1.5	0.012	zgc:56382	zgc:111983
ENSDARG00000008388	-1.5	0.016	mmp14b	matrix metallopeptidase 14b (membrane-inserted)
ENSDARG00000086826	-1.5	0.008	sult6b1	sulfotransferase family, cytosolic, 6b, member 1
ENSDARG00000077982	-1.5	0.036	elf3	E74-like factor 3 (ets domain transcription factor, epithelial-specific)
ENSDARG00000038980	-1.5	0.031	txndc12	thioredoxin domain containing 12 (endoplasmic reticulum)
ENSDARG00000103979	-1.5	0.002	calr3a	calreticulin 3a
ENSDARG00000098591	-1.5	0.001	tubb2b	tubulin, beta 2b
ENSDARG00000086618	-1.5	0.003	psma3	proteasome subunit alpha 3
ENSDARG00000003429	-1.5	0.000	hnrpdl	heterogeneous nuclear ribonucleoprotein D-like
ENSDARG00000044132	-1.5	0.027	ogn	osteoglycin
ENSDARG00000104068	-1.5	0.000	gstp1	glutathione S-transferase pi 1
ENSDARG00000069302	-1.5	0.021	snx9b	sorting nexin 9b
ENSDARG00000043856	-1.5	0.001	amd1	adenosylmethionine decarboxylase 1
ENSDARG00000098935	-1.5	0.025	bxdc2	brix domain containing 2
ENSDARG00000002591	-1.5	0.014	ruvbl1	RuvB-like AAA ATPase 1
ENSDARG00000014591	-1.5	0.003	ilf2	interleukin enhancer binding factor 2
ENSDARG00000026183	-1.5	0.029	polr2eb	polymerase (RNA) II (DNA directed) polypeptide E, b
ENSDARG00000099371	-1.5	0.009	cygb1	cytoglobin 1
ENSDARG00000017741	-1.5	0.006	g3bp1	GTPase activating protein (SH3 domain) binding protein 1
ENSDARG00000031434	-1.5	0.024	rcor1	REST corepressor 1
ENSDARG00000020465	-1.5	0.000	ewsr1b	EWS RNA-binding protein 1b
ENSDARG00000104139	-1.5	0.001	atp1a3b	ATPase, Na+/K+ transporting, alpha 3b polypeptide
ENSDARG00000057414	-1.5	0.001	phb	prohibitin
ENSDARG00000026611	-1.5	0.030	socs3b	suppressor of cytokine signaling 3b
ENSDARG00000023472	-1.5	0.006	ctnnb2	catenin, beta 2
ENSDARG00000102414	-1.5	0.004	myhz1.1	myosin, heavy polypeptide 1.1, skeletal muscle
ENSDARG00000077736	-1.5	0.028	cplx1	complexin-1
ENSDARG00000100731	-1.5	0.011	slc27a2b	solute carrier family 27 (fatty acid transporter), member 2b
ENSDARG00000014517	-1.5	0.002	usp5	ubiquitin specific protease 5
ENSDARG00000036558	-1.5	0.000	col18a1	collagen type XVIII, alpha 1
ENSDARG00000046002	-1.5	0.018	necap2	NECAP endocytosis associated 2
ENSDARG00000009782	-1.5	0.017	myh11a	myosin, heavy chain 11a, smooth muscle

ENSDARG00000086272	-1.5	0.020	si:dkey-4p15.5	si:dkey-4p15.5
ENSDARG00000004034	-1.5	0.000	arhgdig	Rho GDP dissociation inhibitor (GDI) gamma
ENSDARG00000087937	-1.5	0.030	cdk4	cyclin-dependent kinase 4
ENSDARG00000014915	-1.5	0.002	ndufab1b	NADH dehydrogenase (ubiquinone) 1, alpha/beta subcomplex, 1b
ENSDARG00000102977	-1.5	0.031	cant1b	calcium activated nucleotidase 1b
ENSDARG00000037038	-1.5	0.005	psmc6	proteasome 26S subunit, ATPase 6
ENSDARG00000030177	-1.5	0.042	uchl3	ubiquitin carboxyl-terminal esterase L3 (ubiquitin thiolesterase)
ENSDARG00000040118	-1.5	0.001	zgc:113232	zgc:113232
ENSDARG00000076568	-1.5	0.002	sec61b	Sec61 translocon beta subunit
ENSDARG00000025269	-1.5	0.001	pdcd6ip	programmed cell death 6 interacting protein
ENSDARG00000070553	-1.5	0.039	rnmt	RNA (guanine-7-) methyltransferase
ENSDARG00000014165	-1.5	0.004	ssr3	signal sequence receptor, gamma
ENSDARG00000105116	-1.5	0.001	p4hb	prolyl 4-hydroxylase, beta polypeptide
ENSDARG00000103740	-1.5	0.017	fundc2	fun14 domain containing 2
ENSDARG00000052856	-1.5	0.001	khdrbs1a	KH domain containing, RNA binding, signal transduction associated 1a
ENSDARG00000103320	-1.5	0.027	lclat1	lysocardiolipin acyltransferase 1
ENSDARG00000002710	-1.5	0.000	ncl	nucleolin

Control: Incubation 24 ℃ × Challenge 32 ℃ vs Incubation 24 ℃ × Challenge 24 ℃

* Adjusted *p*-value (padj) < 0.05, Benjamin-Hochberg method
* Enrichment value = GeneRatio/BgRatio

Up-regulated

GO ID	Description	GeneRatio	BgRatio	Padj	Gene Name
GO:0010927	cellular component assembly involved in morphogenesis	15/321	204/14763	0.040	ttc9c/unc45b/dmd/smyd1a/hsp90aa1.1/zgc:56235/c10h21orf59/myoz1a/desma/myoz3a/wdr35/alms1/rp1l1a/smyd1b/ccdc40
GO:0061061	muscle structure development	17/321	279/14763	0.040	unc45b/dmd/smyd1a/hsp90aa1.1/tbx6/mapk14b/myf6/klhl31/fbxo32/myoz1a/desma/zgc:113423/myoz3a/mkl1b/si:dkeyp-69b9.3/smyd1b/gli1

Down-regulated

GO ID	Description	GeneRatio	BgRatio	Padj	Gene Name
GO:0044712	single-organism catabolic process	33/465	283/14763	4.27855	gda/acaa1/pgam1a/fbp2/eci1/fbp1b/apoa4b.2/psmc6/gnpda1/gh1/cpt2/hkdc1/acaa2/si:dkey-4e7.3/mmp9/psmc1b/ppp1r3b/enpp7.1/oxct1b/si:ch211-201h21.5/hadhaa/rnf121/hadhab/gk5/gck/cyp7a1/zgc:162608/tdo2a/afp4/aglb/apoa1b/hk2/gcshb
GO:0006082	organic acid metabolic process	42/465	433/14763	4.27855	dhfr/acaa1/pgam1a/lta4h/ugt1ab/odc1/ndufab1b/eci1/fads2/apoa4b.2/idh1/fam213b/hadh/rcor1/hsd17b12b/gnpda1/cpt2/hkdc1/acaa2/padi2/elovl2/pcxb/aldh1a2/msmo1/hadhaa/cmah/rdh10a/hadhab/sgpl1/gck/cyp7a1/zgc:162608/tdo2a/eevs/fa2h/suox/afp4/elovl1a/apoa1b/hk2/ogdhb/gcshb
GO:1901615	organic hydroxy compound metabolic process	19/465	115/14763	4.99136	dhrs9/apoa4b.2/mao/adh8b/sult1st1/degs2/ebp/cyb5r2/hmgcra/aldh1a2/msmo1/rdh10a/gk5/srebf2/cyp7a1/zgc:162608/afp4/apoa1b/dhcr7
GO:0044255	cellular lipid metabolic process	32/465	411/14763	0.00017	acaa1/lta4h/ndufab1b/eci1/dhrs9/fads2/idi1/apoa4b.2/fam213b/hadh/rcor1/hsd17b12b/cpt2/acaa2/degs2/apoba/bpnt1/elovl2/hmgcra/aldh1a2/enpp7.1/msmo1/hadhaa/rdh10a/hadhab/zgc:162608/fa2h/afp4/elovl1a/apoa1b/hmgcs1/lclat1
GO:0098856	intestinal lipid absorption	5/465	10/14763	0.00039	apoa4b.2/anxa2b/zgc:162608/afp4/apoa1b

GO:0044262	cellular carbohydrate metabolic process	10/465	73/14763	0.004	pgam1a/fbp2/fbp1b/hkdc1/ppp1r3b/gk5/gck/chs1/aglb/hk2
GO:0045834	positive regulation of lipid metabolic process	5/465	16/14763	0.004	apoa4b.2/gh1/zgc:162608/afp4/apoa1b
GO:0097006	regulation of plasma lipoprotein particle levels	4/465	10/14763	0.005	apoa4b.2/zgc:162608/afp4/apoa1b
GO:0007586	digestion	6/465	28/14763	0.005	mtp/apoa4b.2/anxa2b/zgc:162608/afp4/apoa1b
GO:0019725	cellular homeostasis	22/465	310/14763	0.008	slc26a3.2/atp2a2b/txndc5/slc34a2a/pdia4/slc30a7/glrx/atp2a2a/atp13a1/hkdc1/txndc12/prdx6/atp6v0ca/aifm1/abcb7/gck/cyp7a1/aqp8a.2/zgc:198419/hk2/atp1a3b/p4hb
GO:0005975	carbohydrate metabolic process	22/465	313/14763	0.009	gale/pgam1a/fbp2/pgm2/ugdh/fbp1b/pck2/gnpda1/hkdc1/ppp1r3b/pcxb/pfkfb4a/si:dkey-202g17.3/gk5/gck/chs1/slc37a4b/chia.2/aglb/chia.1/hk2/taldo1
GO:0009894	regulation of catabolic process	9/465	72/14763	0.009	pgam1a/psmd3/apoa4b.2/gh1/ppp1r3b/rnf121/zgc:162608/afp4/apoa1b
GO:0097164	ammonium ion metabolic process	5/465	22/14763	0.011	apoa4b.2/enpp7.1/zgc:162608/afp4/apoa1b
GO:0022600	digestive system process	5/465	24/14763	0.013	apoa4b.2/anxa2b/zgc:162608/afp4/apoa1b
GO:0009410	response to xenobiotic stimulus	7/465	49/14763	0.014	ugt1ab/sult1st1/sult6b1/si:ch211-117m20.5/cyp1a/cyb5a/gstp1
GO:0048565	digestive tract development	9/465	86/14763	0.022	hand2/myh11a/dhrs9/tbx1/cldn15a/aldh1a2/pllp/agr2/ap1m2
GO:0045454	cell redox homeostasis	7/465	56/14763	0.026	txndc5/pdia4/glrx/txndc12/prdx6/aifm1/p4hb
GO:0006081	cellular aldehyde metabolic process	5/465	29/14763	0.027	pgam1a/pgd/dhrs9/adh8b/taldo1
GO:0010817	regulation of hormone levels	8/465	75/14763	0.032	dhrs9/rcor1/hsd17b12b/aldh1a2/enpep/rdh10a/gck/vipb
GO:0010876	lipid localization	11/465	129/14763	0.033	mtp/sigmar1/apoa4b.2/stard14/apoba/anxa2b/zgc:162608/apoc2/afp4/apoa1b/abcd3a
GO:0044723	single-organism carbohydrate metabolic process	14/465	191/14763	0.037	gale/pgam1a/fbp2/fbp1b/pck2/hkdc1/ppp1r3b/pcxb/pfkfb4a/gk5/gck/chs1/aglb/hk2
GO:0055086	nucleobase-containing small molecule metabolic process	20/465	321/14763	0.037	adss/gda/dhfr/pgam1a/adssl/pgd/atp5g3a/pnp4b/hkdc1/si:dkey-4e7.3/amd1/hmgcra/atp5g1/si:ch211-201h21.5/gmpr2/gck/papss2a/rrm2/hk2/taldo1
GO:0042180	cellular ketone metabolic process	6/465	46/14763	0.037	apoa4b.2/rcor1/zgc:162608/tdo2a/afp4/apoa1b

GO:1901657	glycosyl compound metabolic process	14/465	200/14763	0.049	adss/pgam1a/adssl/atp5g3a/pnp4b/hkdc1/si:dkey-4e7.3/amd1/hmgcra/atp5g1/si:ch211-201h21.5/gck/papss2a/hk2
GO:0034637	cellular carbohydrate biosynthetic process	4/465	22/14763	0.049	fbp2/fbp1b/chs1/aglb

Supplementary Figure S1. Early development of zebrafish at different incubation temperatures. Embryo survival (a), egg hatching rate (b), and time until 75% larvae reaching first-feeding stage (c) at different incubation temperatures are shown. The first-feeding stage is reached by 129 ± 1, 74 ± 1 and 54 ± 1 hours post-fertilization at 24 °C, 28 °C and 32 °C, respectively (mean ± S.E., n=129, 138 and 152, respectively). Superscript letters indicate significant (p-value < 0.05) differences determined by two-way ANOVA.

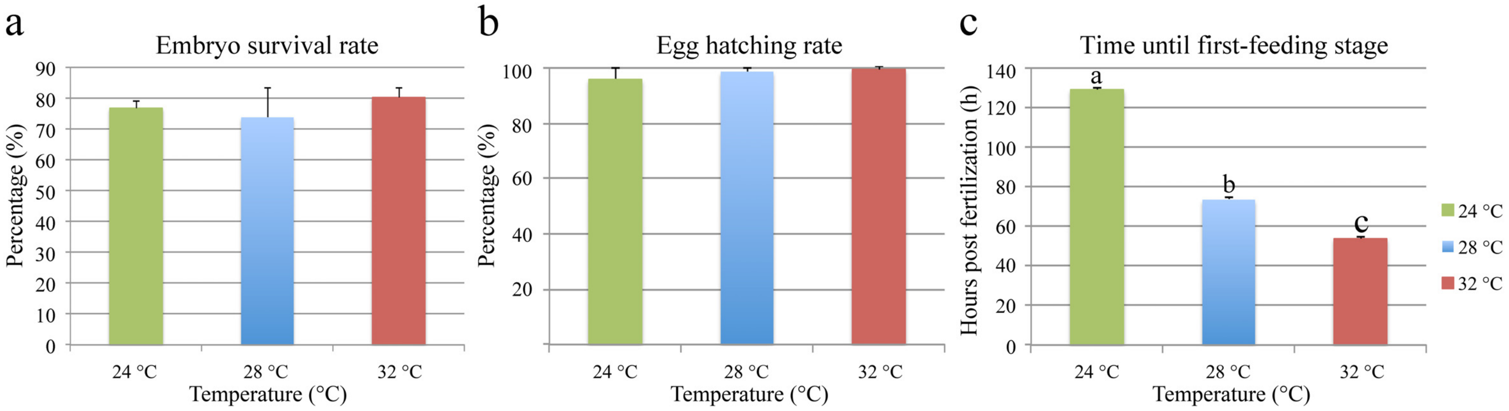

Supplementary Figure S2. Morphology of first feeding larvae at different incubation temperatures. (a) Morphology of first feeding larvae incubated at 24 °C (129 hpf), 28 °C (74 hpf) or 32 °C (54 hpf), and (b) respective body length (4.1 ± 0.2 mm at 24 °C, 4.0 ± 0.1 mm at 28 °C, and 4.1 ± 0.2 mm at 32 °C; mean ± s.d., n=10) are shown.

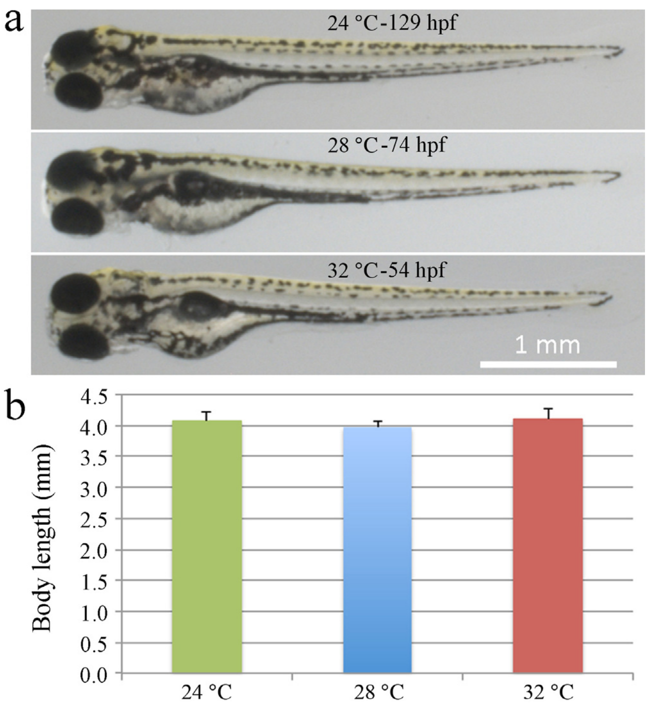

Supplement: Supplementary file 1 — Supplementary Tables S1-S7 and Figures S1-S2 [file 41598_2018_22288_MOESM1_ESM.pdf]
